# Supplementary material for: Beyond snapshots: A dynamic framework for continuous motor unit tracking to enhance neurophysiological assessments
Source: bioRxiv. 2026 Oct 1:2026.09.25.754489. Preprint. [Version 1] doi: 10.64898/2026.09.25.754489 (PMC13648476; doi:10.64898/2026.09.25.754489)
Supplement: Supplementary file 1 [file media-1.pdf]

Supplementary Information

Supplementary Note 1. Motor unit decomposition and firing pattern extraction 3

Supplementary Note 2. Calculations of pulse to noise ratio, sensitivity, and precision 6

Supplementary Note 3. Implementation 7

Supplementary Note 4. Model convergence and selection 14

Supplementary Note 5. Model implementation, likelihood, priors used in Bayesian analysis on performance comparison between proposed algorithm and SoA 15

Supplementary Note 6. Comparison of firing identification PNR for tracked TA MUs across synthetic grid displacement. 24

Supplementary Note 7. Comparison of firing identification sensitivity in tracked TA MUs across synthetic grid displacement. 32

Supplementary Note 8. Comparison of firing identification precision in tracked TA MUs across synthetic grid displacement. 42

Supplementary Note 9. Comparison of firing identification PNR for tracked MG MUs across synthetic grid displacement. 52

Supplementary Note 10. Comparison of firing identification sensitivity in tracked MG MUs across synthetic grid displacement. 60

Supplementary Note 11. Comparison of firing identification precision in tracked MG MUs across synthetic grid displacement. 70

Supplementary Note 12. Comparison of firing identification PNR for tracked TA MUs across testing visits. 80

Supplementary Note 13. Comparison of firing identification sensitivity in tracked TA MUs across testing visits. 88

Supplementary Note 14. Comparison of firing identification precision in tracked TA MUs across testing visits. 98

Supplementary Note 15. Comparison of firing identification PNR for tracked MG MUs across testing visits. 108
Supplementary Note 16. Comparison of firing identification sensitivity in tracked MG MUs across testing visits. 116
Supplementary Note 17. Comparison of firing identification precision in tracked MG MUs across testing visits. 126
Supplementary Note 18. Bayesian analysis R session info 136
Supplementary Note 19. Linear mixed effect model for the rate and recruitment threshold of tracked MUs across follicular phase 138
Supplementary Note 20. Linear mixed effect model of firing rate and recruitment threshold of all identified motor units across follicular phase 143
Supplementary References 148

Supplementary Note 1. Motor unit decomposition and firing pattern extraction

Decomposition of high-density electromyography

Surface EMG signals at each channel in the High-Density surface EMG (HDsEMG) grid ($y_i(n)$) can be modeled as the summation of all motor unit (MU) activities detected by the corresponding electrode, which can be written as

$$y_i(n) = \sum_{j=1}^{J} \sum_{l=0}^{L-1} a_{i,j}(l) t_j(n-l) \tag{S1}$$

where $a_{i,j}$ is the j-th MU action potential (MUAP) with duration of L samples recorded by the i-th electrode of the HDsEMG grid, n is time sample, and pulse train $t_j(n)$ indicates the time instances of the MUAP occurrences. The MU pulse train is defined as

$$t_j(n) = \sum_{k} \delta\left(n - \tau_j(k)\right) \tag{S2}$$

where δ denotes the unit-sample pulse and $\tau_j(k)$ denotes the time instances of the k-th MU firing.

The CKC algorithm estimates MU firing instances by inverting the EMG mixing model. It generates MU pulse train for each MU. The MUAP spatiotemporal shapes ($a_{i,j}(l)$) are calculated using the spike-triggered averaging method, which utilizes the information of the identified MU firings.[1]

Assume that the electrodes on the HDsEMG grid are arranged in P rows and Q columns. For each motor unit j, the MUAP spatiotemporal shape can then be written in matrix form as follows:

$$\mathrm{A}_j(l) = \begin{bmatrix} [a_{j,1,1}(l)] & \cdots & [a_{j,1,Q}(l)] \\ \vdots & \ddots & \vdots \\ [a_{j,P,1}(l)] & \cdots & [a_{j,P,Q}(l)] \end{bmatrix} \quad l = 1, \ldots, L \tag{S3}$$

where $a_{j,p,q}(l)$ is the MUAP of the j-th MU as recorded by an electrode located at p-th row and q-th column at the time sample l. The matrix $\mathrm{A}_j(l)$ will be defined herein as the MUAP matrix.

Extracting MU firing pattern using MUAP filters

Once the MUAP spatiotemporal shapes are identified ($\mathrm{A}_j(l)$), the firing pattern of each MU in the subsequent recording can be extracted by applying the corresponding MU filter (G_j) on the HDsEMG signals. To improve the filter efficiency, both the set of HDsEMG channels and the MUAP matrix $\mathrm{A}_j(l)$ are extended by adding delayed repetitions of recorded HDsEMG channels and MUAP estimates.[2] In this study, we added 14 delayed repetitions of each HDsEMG channel and MUAP. This resulted in extended HDsEMG channels Y and extended MUAP matrix B_j. The MU filter is constructed by multiplying the transpose of B_j matrix by the inverse of the correlation matrix of the extended HDsEMG signals (${\mathrm{C}_Y}^{-1}$).

$$\mathrm{G}_j = {\mathrm{B}_j}^T {\mathrm{C}_Y}^{-1} \tag{S4}$$

The pulse train of each MU is then calculated by multiplying the MU filter by the extended HDsEMG signals (Y).

$$t'_j(n) = \mathrm{G}_j \mathrm{Y}(n) \tag{S5}$$

Automatic MU filter reinforcement

The MUAP spatiotemporal shapes $a_{i,j}(l)$ and consequently matrix $\mathrm{A}_j(l)$ are calculated by spike-triggered averaging of HDsEMG signals. Thus, they depend on the segmentation of the MU pulses in $t_j(n)$ into MU firings. Improving the segmentation of MU firings enhances the quality of $a_{i,j}(l)$ and $\mathrm{A}_j(l)$ estimation and, thus, the quality of MU filter G_j. In this study, we

used an automatic threshold-based MU pulse segmentation technique. First, pulses of $t_j(n)$ in equation (S2) were normalized by their maximal value. Afterward, the segmentation threshold was decremented from 0.9 to 0.05 in steps of 0.05. After each decrement, MU pulses exciding the threshold were segmented into MU firings. The coefficient of variation (CoV) of the interspike interval of segmented MU firings was calculated for each segmentation threshold, along with the median MU firing rate (MU_FR). The cost of MU firing segmentation included the CoV, and the MU segmentations with MU_FR < 5 Hz or MU_FR > 35 Hz were penalized by adding the cost of 100. The segmentation threshold with the minimal cost value was then selected and used for the final segmentation of MU firings. Afterward, the MUAP spatiotemporal shapes $a_{i,j}(l)$, the matrix $\mathrm{A}_j(l)$ and the MU filter G_j were recalculated and reapplied to the extended HDsEMG signals Y (equation (S5)).

Manual inspection and editing of decomposition results

The obtained MU pulse trains were visually inspected and segmented by an expert operator. Specifically, for each MU, the operator selected the portions of the pulse train that were distinguishable from the background pulse train noise.[3] Once all pulse trains were selected, the spatiotemporal shape of the MUAP (and thus the MU filter) was recalculated using the segmented MU firing instances. Afterwards, the pulse train of the MU was recalculated. The segmentation process was repeated until the optimum decomposition quality of the identified MU was reached.

Supplementary Note 2. Calculations of pulse to noise ratio, sensitivity, and precision

The identified motor unit's decomposition quality was quantified using the pulse-to-noise ratio (PNR). PNR indicates the level of noise in the MU pulse train[4] and is defined as follows:

$$\mathrm{PNR}\left(t_j(n)\right) = 20 \times \log\left(\frac{E\left(t_j^2(n)\big|_{t_j(n)=1}\right)}{E\left(t_j^2(n)\big|_{t_j(n)=0}\right)}\right) \tag{S6}$$

where E represents the mathematical expectation, $t_j^2(n)\big|_{t_j(n)=1}$ denotes the segmented MU pulses denoting MU firing and $t_j^2(t)\big|_{t_j(t)=0}$ denotes the baseline noise at the n-th time sample. Subsequently, MUs with a PNR < 28 dB were excluded from further analysis.[4]

Assume two HDsEMG signals were decomposed independently. MU filter transfer from the first to the second HDsEMG signals was defined as a process of applying MU filters calculated from the identified MU in the first HDsEMG signals to the second HDsEMG signals (Supplementary equations (S4) and (S5)), and segmentation of the MU firings was subsequently repeated. For MUs that were identified in both HDsEMG signals, the similarity between the firing patterns of MUs identified from the independent decomposition of the second HDsEMG signals and MUs filter transfer from the first HDsEMG signals was assessed by calculating the precision and sensitivity of MU firing identification (precision and sensitivity, respectively)[5]

$$\mathrm{precision}_j = \frac{\mathrm{TP}}{\mathrm{TP+FP}} \quad , \quad \mathrm{sensitivity}_j = \frac{\mathrm{TP}}{\mathrm{TP+FN}} \tag{S7}$$

where TP, FP, and FN denote the number of true positive, false positive, and false negative firings, respectively. The tolerance limit of the MU firing match was set to 1 ms, and the results of the subsequent HDsEMG signals independent decompositions were used as ground truth reference.

Supplementary Note 3. Implementation

This Supplementary Note provides additional details for the algorithm depicted in Fig. 1 and Fig. 2. Vectors are denoted by bold letters.

The HDsEMG signals recorded at two testing visits (Visit 1 and Visit 2) are denoted as $y1(n)$ and $y2(n)$, respectively. Each HDsEMG signal was independently decomposed in a monopolar configuration into its constituent MUs pulse trains using the CKC algorithm (Fig.1b).[2] The spatiotemporal shape of the MUAP was extracted from the single differential HDsEMG signal computed along the longitudinal axis of the muscle fibers. In this study, the single differential HDsEMG signal was computed along the column of electrode grid. MUAP matrices for datasets $y1(n)$ and $y2(n)$ are denoted as $\mathrm{A1}_r(l)$ and $\mathrm{A2}_s(l)$ where $r = 1, \dots, R$ and $s = 1, \dots, S$ denotes the identified MUs in each dataset, respectively.

The first step of the proposed algorithm was to calculate an initial estimate of the electrode grid displacement between visits (Fig. 1d). To begin this process, the maximum temporal peak-to-peak value across channels for each MUAP matrix was calculated. The MUs in each testing visit were sorted in descending order based on their maximum temporal peak-to-peak value across channels, resulting in new MUs orders r_{srt} and s_{srt}. The initial electrode grid displacement was then estimated starting with MUs with the largest temporal peak-to-peak MUAPs across channels in datasets $y1(n)$ and $y2(n)$.

Each MUAP matrix was considered as a series of images. Specifically, each electrode of the grid may be conceived as a pixel in an image with coordinates (p, q) given by the rows and columns in the HDsEMG grid of electrodes with a magnitude that evolves across time sample l (Fig. 2b). The temporal root mean square (RMS) envelope of each MUAP image in the datasets was calculated and denoted as $U1$ and $U2$, respectively.

$$U1_{r_{\text{srt}}}(l) = \sqrt{\frac{1}{PQ}\sum_{p=1}^{P}\sum_{q=1}^{Q}\text{A1}_{r_{\text{srt}},p,q}{}^{2}(l)} \quad \text{and} \quad U2_{s_{\text{srt}}}(l) = \sqrt{\frac{1}{PQ}\sum_{p=1}^{P}\sum_{q=1}^{Q}\text{A2}_{s_{\text{srt}},p,q}{}^{2}(l)} \tag{S8}$$

The temporal profiles of the MUAP images' RMS envelopes ($U1_{r_{\text{srt}}}(l)$ and $U2_{s_{\text{srt}}}(l)$) were observed to vary in time. Therefore, to align them, dynamic time warping (DTW)[6] was employed and DTW-aligned time samples are denoted as l_{DTW}. DTW aligns the time series between two out-of-phase datasets by calculating their minimum cost path. In this study, DTW-aligned time samples (l_{DTW}) for which the RMS of the MUAP images in both datasets ($U1_{r_{\text{srt}}}(l)$ and $U2_{s_{\text{srt}}}(l)$) reached at least 30% of their respective maximum value ($l_{\text{DTW},\geq 30\text{p}}$) were used in further processing and were also used to temporarily align MUAP images from both visits.

The inter-electrode distance of the HDsEMG grid was assumed to satisfy the Nyquist criterion for HDsEMG spatial sampling;[7] therefore, the continuous spatial distribution of the MUAP can be obtained by spatially interpolating the DTW-aligned MUAP images using a two-dimensional sinc function.[8] In this study, the MUAP images were interpolated to obtain a new spatial resolution of 0.8 mm (denoted as $\text{A1}_{\text{HiRes},r_{\text{srt}}}\left(l_{\text{DTW},\geq 30\text{p}}\right)$ and $\text{A2}_{\text{HiRes},s_{\text{srt}}}\left(l_{\text{DTW},\geq 30\text{p}}\right)$ for the first and the second testing visit, respectively). Subsequently, intensity-based image registration with rigid transformation supplied within MATLAB Image Processing Toolbox was performed on the interpolated MUAP images ($\text{A1}_{\text{HiRes},r_{\text{srt}}}\left(l_{\text{DTW},\geq 30\text{p}}\right)$ and $\text{A2}_{\text{HiRes},s_{\text{srt}}}\left(l_{\text{DTW},\geq 30\text{p}}\right)$) to estimate the affine transformation (vertical and horizontal translations and rotation) with interpolated MUAP image $\text{A2}_{\text{HiRes},s_{\text{srt}}}\left(l_{\text{DTW},\geq 30\text{p}}\right)$ as reference. The optimizing method of the image registration was set to regular step gradient descent, and the quantitative measure of similarity was set to mean square error. The spatially interpolated MUAP images $\text{A1}_{\text{HiRes},r_{\text{srt}}}\left(l_{\text{DTW},\geq 30\text{p}}\right)$ were then transformed using the estimated affine

transformation and resampled to restore the spatial resolution to 8 mm ($\mathrm{A1}_{\mathrm{resampled},r_{\mathrm{srt}}}(l_{\mathrm{DTW},\geq 30\mathrm{p}})$).

The normalized root mean square error (NRMSE) between MUAP images $\mathrm{A1}_{\mathrm{resampled.},r_{\mathrm{srt}}}(l_{\mathrm{DTW},\geq 30\mathrm{p}})$ and $\mathrm{A2}_{s_{\mathrm{srt}}}(l_{\mathrm{DTW},\geq 30\mathrm{p}})$ was calculated as follows.

$$X_{r_{\mathrm{srt}},s_{\mathrm{srt}}}(l_{\mathrm{DTW},\geq 30\mathrm{p}}) = \frac{\left\|\mathrm{A2}_{s_{\mathrm{srt}}}(l_{\mathrm{DTW},\geq 30\mathrm{p}}) - \mathrm{A1}_{\mathrm{resampled},r_{\mathrm{srt}}}(l_{\mathrm{DTW},\geq 30\mathrm{p}})\right\|}{\left\|\mathrm{A2}_{s_{\mathrm{srt}}}(l_{\mathrm{DTW},\geq 30\mathrm{p}}) - \mathrm{mean}\left(\mathrm{A2}_{s_{\mathrm{srt}}}(l_{\mathrm{DTW},\geq 30\mathrm{p}})\right)\right\|} \quad \text{(S9)}$$

where ‖ indicates the 2-norm. If all the NRMSE values across the DTW-aligned time samples $(l_{\mathrm{DTW},\geq 30\mathrm{p}})$ were greater than 30%, the initial electrode grid displacement estimation was repeated using the subsequent MU pair. The affine transformations that yielded an NRSME of less than 30% were then averaged across the time sample l and were considered as the initial estimate of the electrode grid displacement ($\mathrm{GD_{init}}$, Fig. 1d and Fig. 2c).

The second step was to find matched MUs by evaluating the MUAP shape and amplitude (Fig. 1e). All MUAPs in Visit 1 were forward transformed using $\mathrm{GD_{init}}$, yielding a transformed MUAP spatiotemporal shape $\mathrm{A1}_{\mathrm{GD_{init}},r}(l)$. The MUAP spatiotemporal shapes ($\mathrm{A1}_{\mathrm{GD_{init}},r}(l)$ and $\mathrm{A2}_s(l)$) were vectorized by row-wise concatenating the MUAP profile of each non-empty HDsEMG channel (Fig. 2d), yielding $\mathbf{A1}_{\mathrm{GD_{init}},r}$ and $\mathbf{A2}_s$. To evaluate the similarity of the MUAP shape, the cross-correlation coefficient ($\rho_{r,s}$) between $\mathbf{A1}_{\mathrm{GD_{init}},r}$ and $\mathbf{A2}_s$ were evaluated for all possible MU pairs.

$$\rho_{r,s} = \frac{E\{\mathbf{A1}_{\mathrm{GD_{init}},r}\ \mathbf{A2}_s\} - m_{\mathbf{A1}_{\mathrm{D_{init}},r}}\, m_{\mathbf{A2}_s}}{\sigma_{\mathbf{A1}_{\mathrm{GD_{init}},r}}\sigma_{\mathbf{A2}_\mathrm{s}}} \quad \text{(S10)}$$

where E denotes the mathematical expectation, $m_{\mathbf{A1}_{\mathrm{D_{init}},r}}$ and $m_{\mathbf{A2}_s}$ are the average values of $\mathbf{A1}_{\mathrm{GD_{init}},r}$ and $\mathbf{A2}_s$ respectively, $\sigma_{\mathbf{A1}_{\mathrm{GD_{init}},r}}$ and $\sigma_{\mathbf{A2}_\mathrm{s}}$ are the standard deviations. MU pairs with $\rho_{r,s}$ greater than 0.70 were selected as the initial matched MU pairs.

The MUAP amplitude detected by each electrode depends on the distance between the MU fibers and the electrode.[9] Hence, a pair of MUs with similar shapes but significantly different amplitudes are unlikely to be generated by the same source. To evaluate the differences in the amplitude, the time-wise RMS envelopes were recalculated excluding the missing and discarded electrodes data resulting from the initial affine transformation.

$$W1_r(l) = \sqrt{\frac{1}{P_{\mathrm{adj}}Q_{\mathrm{adj}}}\sum_{p=1}^{P_{\mathrm{adj}}}\sum_{q=1}^{Q_{\mathrm{adj}}}\mathrm{A1}_{\mathrm{GD_{init}},r,p,q}{}^2(l)} \quad \text{and} \quad W2_s(l) = \sqrt{\frac{1}{P_{\mathrm{adj}}Q_{\mathrm{adj}}}\sum_{p=1}^{P_{\mathrm{adj}}}\sum_{q=1}^{Q_{\mathrm{adj}}}\mathrm{A2}_{s,p,q}{}^2(l)} \qquad \text{(S11)}$$

where P_{adj} and Q_{adj} were the total number of rows and columns of the MUAP spatiotemporal shape with missing and discarded electrodes excluded. Afterward, following DTW time sample alignment, the normalized difference of the RMSE values were calculated.

$$Z_{r,s} = \frac{\|W2_s(l_{\mathrm{DTW}}) - W1_r(l_{\mathrm{DTW}})\|}{\left\|W2_s(l_{\mathrm{DTW}}) - \mathrm{mean}\big(W2_s(l_{\mathrm{DTW}})\big)\right\|} \qquad \text{(S12)}$$

where $\|$ indicates the 2-norm and l_{DTW} is DTW aligned time sample. MU pairs with Z values greater than 0.3 were considered false positive pairs and were discarded. Subsequently, MUs in Visit 1 with multiple possible matches with MUs in Visit 2 were identified. For each MU with multiple possible matches, MU pair with highest $\rho_{r,s}$ was considered as a true pair. If there were more than one MU pair with equaly high $\rho_{r,s}$, the $Z_{r,s}$ values of these MU pairs were compared and MU pair with lowest $Z_{r,s}$ was used in further analysis.

The third step was to refine the candidates of the matched MU by evaluating the affine transformation of each MU pair. For each MU pair, the transformed MUAP images of Visit 1 ($\mathrm{A1}_{\mathrm{GD_{init}},r}(l)$) and Visit 2 ($\mathrm{A2}_s(l)$) were aligned in time using DTW algorithms using the temporal RMS envelope (equation 11). The MUAP images were then interpolated using two-

dimensional sinc function ($A1_{\mathrm{GDHiRes}.r}(l_{\mathrm{DTW}})$ and $A2_{\mathrm{HiRes}.s}(l_{\mathrm{DTW}})$). Similar to the first step of the proposed algorithm, the affine transformation between $A1_{\mathrm{GDHiRes}.r}(l_{\mathrm{DTW}})$ and $A2_{\mathrm{HiRes}.s}(l_{\mathrm{DTW}})$ was estimated using intensity-based registration with $A2_{\mathrm{HiRes}.s}(l_{\mathrm{DTW}})$ used as reference (Fig. 1c). The estimated translation and rotation values were averaged across all the MUs in Visit 1 and defined as the refined compensation of the HDsEMG grid displacement (Fig. 1f, GD_{refined}). Finally, GD_{refined} was applied to all the MU filters in Visit 1, MU filters were calculated using Supplementary equation (S4), and the algorithm's performance was evaluated. The overall algorithm is presented in pseudocode below.

Algorithm 1. Motor unit tracking across testing visits

---

1. Calculate the maximum MUAP peak-to-peak value across the electrodes of all the identified MUs in Visit 1 and Visit 2
2. Sort in descending order all the MUs in Visit 1 and Visit 2 based on the maximum MUAP temporal peak-to-peak value across channels, $A1_{r_{\mathrm{srt}}}$ and $A2_{s_{\mathrm{srt}}}$
3. found initial estimation of the grid displacement (GD_{init}) ← FALSE
4. while found GD_{init} = FALSE do
5.   for all possible pairs between $A1_{r_{\mathrm{srt}}}$ and $A2_{s_{\mathrm{srt}}}$
6.   Calculate the temporal RMS of the MUAP images (Supplementary equation (S8)), $U1_{r_{\mathrm{srt}}}(l)$ and $U2_{s_{\mathrm{srt}}}(l)$
7.   Align time sample using DTW algorithm, $U1_{r_{\mathrm{srt}}}(l_{\mathrm{DTW}})$ and $U2_{s_{\mathrm{srt}}}(l_{\mathrm{DTW}})$
8.   Find DTW time sample where $U1_{r_{\mathrm{srt}}}(l_{\mathrm{DTW}})/\max(U1_{r_{\mathrm{srt}}})$ and $U2_{s_{\mathrm{srt}}}(l_{\mathrm{DTW}})/\max(U2_{s_{\mathrm{srt}}})$ are greater than 30%, $l_{\mathrm{DTW},\geq 30\mathrm{p}}$
9.   for all DTW time samples $l_{\mathrm{DTW},\geq 30\mathrm{p}}$

10. Spatially up-sample of MUAP images, $\mathrm{A1}_{\mathrm{HiRes},r_{\mathrm{srt}}}(l_{\mathrm{DTW},\geq 30\mathrm{p}})$ and $\mathrm{A2}_{\mathrm{HiRes},s_{\mathrm{srt}}}(l_{\mathrm{DTW},\geq 30\mathrm{p}})$

11. Estimate translation and rotation using intensity-based registration

12. Apply translation and rotation to $\mathrm{A1}_{\mathrm{HiRes},r_{\mathrm{srt}}}(l_{\mathrm{DTW},\geq 30\mathrm{p}})$

13. Resample $\mathrm{A1}_{\mathrm{HiRes},r_{\mathrm{srt}}}(l_{\mathrm{DTW},\geq 30\mathrm{p}})$ to original spatial resolution, $\mathrm{A1}_{\mathrm{resampled},r_{\mathrm{srt}}}(l_{\mathrm{DTW},\geq 30\mathrm{p}})$

14. Calculate the NRMSE between $\mathrm{A1}_{\mathrm{resamp.},r_{\mathrm{srt}}}(l_{\mathrm{DTW},\geq 30\mathrm{p}})$ and $\mathrm{A2}_{s_{\mathrm{srt}}}(l_{\mathrm{DTW},\geq 30\mathrm{p}})$ (Supplementary equation (S9)), $X_{r_{\mathrm{srt}},s_{\mathrm{srt}}}(l_{\mathrm{DTW},\geq 30\mathrm{p}})$

15. if $X_{r_{\mathrm{srt}},s_{\mathrm{srt}}}(l_{\mathrm{DTW},\geq 30\mathrm{p}}) > 0.3$ then

16. Discard the estimated translation and rotation (Fig. 2c)

17. if $\mathrm{length}(X_{r_{\mathrm{srt}},s_{\mathrm{srt}}}(l_{\mathrm{DTW},\geq 30\mathrm{p}}) \leq 0.3) > 0$ then

18. $\mathrm{GD}_{\mathrm{init}} \leftarrow$ mean translations and rotation across $l_{\mathrm{DTW},\geq 30\mathrm{p}}$

19. found $\mathrm{GD}_{\mathrm{init}}$ = TRUE

20. Apply $\mathrm{GD}_{\mathrm{init}}$ to all the MUAP matrices in Visit 1, $\mathrm{A1}_{\mathrm{GD}_{\mathrm{init}},r}(l)$

21. for all possible pairs of MUs between Visit 1 and Visit 2

22. Vectorize each MUAP matrix without missing electrodes (Fig. 2d), $\mathrm{A1}_{\mathrm{GD}_{\mathrm{init}},r}$ and $\mathrm{A2}_s$

23. Calculate the correlation coefficient of the vectorized MUAPs (Supplementary equation (S10)), $\rho_{r,s}$

24. if $\rho_{r,s} \geq 0.70$ then

25. MU list ← MU pair

26. for all MU pairs in MU list

27.   Calculate the time-wise RMS envelope of the MUAP images without missing/discarded electrode (Supplementary equation (S11)), $W1_r(l)$ and $W2_s(l)$

28.   Align time sample using DTW algorithm, $W1_r(l_{\mathrm{DTW}})$ and $W2_s(l_{\mathrm{DTW}})$

29.   Calculate the normalized difference of the RMS values between $W1_r(l_{\mathrm{DTW}})$ and $W2_s(l_{\mathrm{DTW}})$ (Supplementary equation (S12)), $Z_{r,s}$

30.   if $Z_{r,s} > 0.3$ then

31.   remove MU pair from MU list

32. for all MUs in MU list with multiple matches

33.   Find the highest correlation coefficient, ρ_{max}

34.   if $\rho_{r,s} < \rho_{max}$ then

35.   remove MU pair from MU list

36.   if number of matched MU >1 then

37.   Find lowest normalized difference of the RMSE values, Z_{min}

38.   if $Z_{r,s} > Z_{min}$ then

39.   remove MU pair from MU list

40. for all MU pairs in MU list

41.   Repeat steps 6 to 18 to obtain an estimation on electrode grid translation and rotation

42. $\mathrm{GD}_{\mathrm{refined}}$ ← average translation and rotation across MU pairs in MU list

43. Apply $\mathrm{GD}_{\mathrm{refined}}$ to all MUAPs in Visit 1

44. Calculate MU filters (Supplementary equation (S4)) and reassess the MU filter for transfer to HDsEMG signals from Visit 2

Supplementary Note 4. Model convergence and selection

The convergence of each model's Markov Chain Monte Carlo (MCMC) chains was evaluated using the potential scale reduction factor (PSRF) and effective sample size (ESS) calculated using Gelman and Rubin's convergence diagnostic and *effectiveSize* function supplied in the *coda* package, respectively.[10,11] Convergence of the MCMC chains is achieved if the PSRF <1.05 and ESS >400.[12,13] In the case where only PSRF satisfies the criteria, the model was not included in further analysis.

The leave-one-out cross-validation (LOO-CV) method[14] was then used to evaluate the goodness of fit of each model. The model with the best goodness of fit was defined as one with the highest Bayesian leave-one-out estimate of the expected log pointwise predictive density. If $0.7 < \text{Pareto } k < 1.0$ of any observation and the posterior predictive check was consistent with the observed data, the *reloo* function supplied by the *loo* package was used to compute exact cross-validation for these problematic observations. If Pareto k estimated after refit was >1 for at least one observation, the model was not included in further analysis.

Supplementary Note 5. Model implementation, likelihood, priors used in Bayesian analysis on performance comparison between proposed algorithm and SoA

Performance on pulse-to-noise ratio (PNR)

The state-of-the-art (SoA) method calculated MU filters from MUs identified in HDsEMG signal recorded at the first testing visit and applied it directly (without MUAP shapes compensation) to subsequent HDsEMG signals to extract MU characteristics.[5] We compared the performance of our proposed algorithm to SoA approach on the PNR of the motor unit firing identification. The predicted variable was PNR (continuous values) and the predictor variable was Method (SoA or Proposed, categorical variable). Three models were fitted and compared to obtain model that best reflects the data. In all models, for performance comparison of tracked MUs across testing visits, Subject as participant identification was used instead of MUset.

Model 1

The conditional mean of the log-transformed response was modeled as a function of Method with MUset-level random intercept. To allow residual variation to differ between Method, the lognormal-scale parameter was modeled as a function of Method. The model was implemented using the formula bf(PNR ~ Method + (1 | MUset), sigma ~ Method). The complete specification was given by the likelihood and prior distributions below.

Based on visual inspection of the data distribution, PNR was strictly positive and right skewed. Therefore, the PNR observations were assumed to be independently sampled from a lognormal distribution.

$$y_i|\mu_i,\sigma_i \sim \text{LogNormal}(\mu_i,\sigma_i) \quad \text{(S13)}$$

with

$$\mu_i = \alpha_\mu + \mathrm{x}_i{}^T\beta_\mu + b_{s_i} \quad \text{(S14)}$$

and

$$\log \sigma_i = \alpha_\sigma + \mathrm{z}_i{}^T \beta_\sigma \quad \text{(S15)}$$

Here y_i is PNR for motor unit i, s_i denotes the MU set for motor unit i, μ_i is conditional mean of the log-transformed response ($\log(y_i)$), σ_i is conditional residual standard deviation of $\log(y_i)$, α_μ denotes population-level intercept for the mean of the $\log(y_i)$, x_i encodes the method of calculating MU filter (SoA or proposed algorithm), β_μ are method effects on the mean of $\log(y_i)$, and b_{s_i} is MU set specific random intercept. Additionally, α_σ is the population-level intercept for the log residual standard deviation, z_i denotes method in the sigma model, and β_σ are method effects on $\log \sigma_i$.

Weakly informative priors were used in the model and described as follows.

$$\alpha_\mu \sim \mathrm{Normal}(\log(30), 0.25^2) \quad \text{(S16)}$$

$$\beta_{\mu,k} \sim \mathrm{Normal}(0, 0.20^2),\ k = 1, \dots, K_\mu \quad \text{(S17)}$$

$$b_j | \tau_b \sim \mathrm{Normal}(0, \tau_b{}^2),\ j = 1, \dots, J \quad \text{(S18)}$$

$$\tau_b \sim \mathrm{Exponential}(2) \quad \text{(S19)}$$

$$\alpha_\sigma \sim \mathrm{Normal}(\log(0.15), 0.35^2) \quad \text{(S20)}$$

$$\beta_{\sigma,k} \sim \mathrm{Normal}(0, 0.25^2),\ k = 1, \dots, K_\sigma \quad \text{(S21)}$$

Here, K_μ is number of non-intercept method coefficients in the model for μ_i, b_j is MU set-specific random intercept for MU set j, τ_b is between-MU set standard deviation of the random intercepts b_j, J is number of MU set, K_σ is number of non-intercept method coefficients in the sigma model.

Priors were specified to be weakly informative on the scale of the observed PNR values. For each analysis, these priors were evaluated using prior predictive simulation before posterior inference to confirm that the priors generated PNR in a plausible range (Supplementary Fig. 1a).

Model 2

In this model we additionally allowed the Method effect to vary by MUset by adding MUset-specific Method slopes. The model was implemented using the formula bf(PNR ~ Method + (1 + Method | MUset), sigma ~ Method). The model specification was similar to Model 1, except

$$\mu_i = \alpha_\mu + \mathbf{x}_i{}^T \beta_\mu + \mathbf{r}_i{}^T \mathbf{b}_{s_i} \tag{S22}$$

where $\mathbf{r}_i$ is group-level design vector for observation i.

The likelihood and priors were set according to Model 1, except for $b_j | \tau_b \sim \text{Normal}(0, {\tau_b}^2)$ which was replaced with random intercept and random method effects defined as

$$\mathbf{b}_j \big| \boldsymbol{\tau}_b, \mathbf{R} \sim \text{MultivariateNormal}\big(0, \text{diag}(\boldsymbol{\tau}_b)\ \mathbf{R}\ \text{diag}(\boldsymbol{\tau}_b)\big) \tag{S23}$$

with

$$\mathbf{b}_j = \left(b_{0j}, b_{1j}, \dots, b_{K_\mu j}\right)^{\mathrm{T}} \tag{S24}$$

where $\mathbf{R}$ contains the correlation between the random intercept and the random method effects.

We additionally assigned an LKJ prior to the correlation matrix among the MUset-specific intercept and Method effects, defined as

$$\mathbf{R} \sim \text{LKJ}(2) \tag{S25}$$

For each analysis, prior predictive simulations confirmed that the priors generated plausible values of PNR (Supplementary Fig. 1b).

Model 3

Compared to Model 2, this model additionally allowed observations from each MUset-MUID combination to have their own random intercept. The model was implemented using the formula bf(PNR ~ Method + (1 + Method | MUset) + (1 | MUset:MUID), sigma ~ Method). Therefore,

$$\mu_i = \alpha_\mu + \mathbf{x}_i{}^T \beta_\mu + \mathbf{r}_i{}^T \mathbf{b}_{s_i} + c_{s_i l_i} \tag{S26}$$

Here $c_{s_i l_i}$ denotes random intercept for the MUset-MUID combination associated with observation i.

The likelihood and priors from Model 2 were used in this model because Model 2 included a broad prior for the class-level random-effect standard deviation (class = “sd”). Then, we performed prior predictive simulations for each analysis to confirm that the priors generated PNR in a plausible range (Supplementary Fig. 1c).

Performance on sensitivity and precision of motor unit firing identification

We compared the performance of our proposed algorithm to SoA separately for each predicted continuous variable Y: sensitivity and precision. The predictor variables were Method (SoA or Proposed, categorical variable). Three models were fitted and compared to obtain model that best reflects the data. In all models, for performance comparison of tracked MUs across testing visits, Subject as participant identification was used instead of MUset.

<u>Model 1</u>

The model used zero-one-inflated beta response distribution that includes Method as a population-level predictor and MUset-level random intercept. To capture Method specific effects, the mean, precision parameter *phi*, zero-one inflation probability *zoi*, and the conditional one-inflation probability *coi* were each modeled as functions of method. The model was implemented using the formula bf(Y ~ Method + (1 | MUset), phi ~ Method, zoi ~ Method, coi ~ Method).

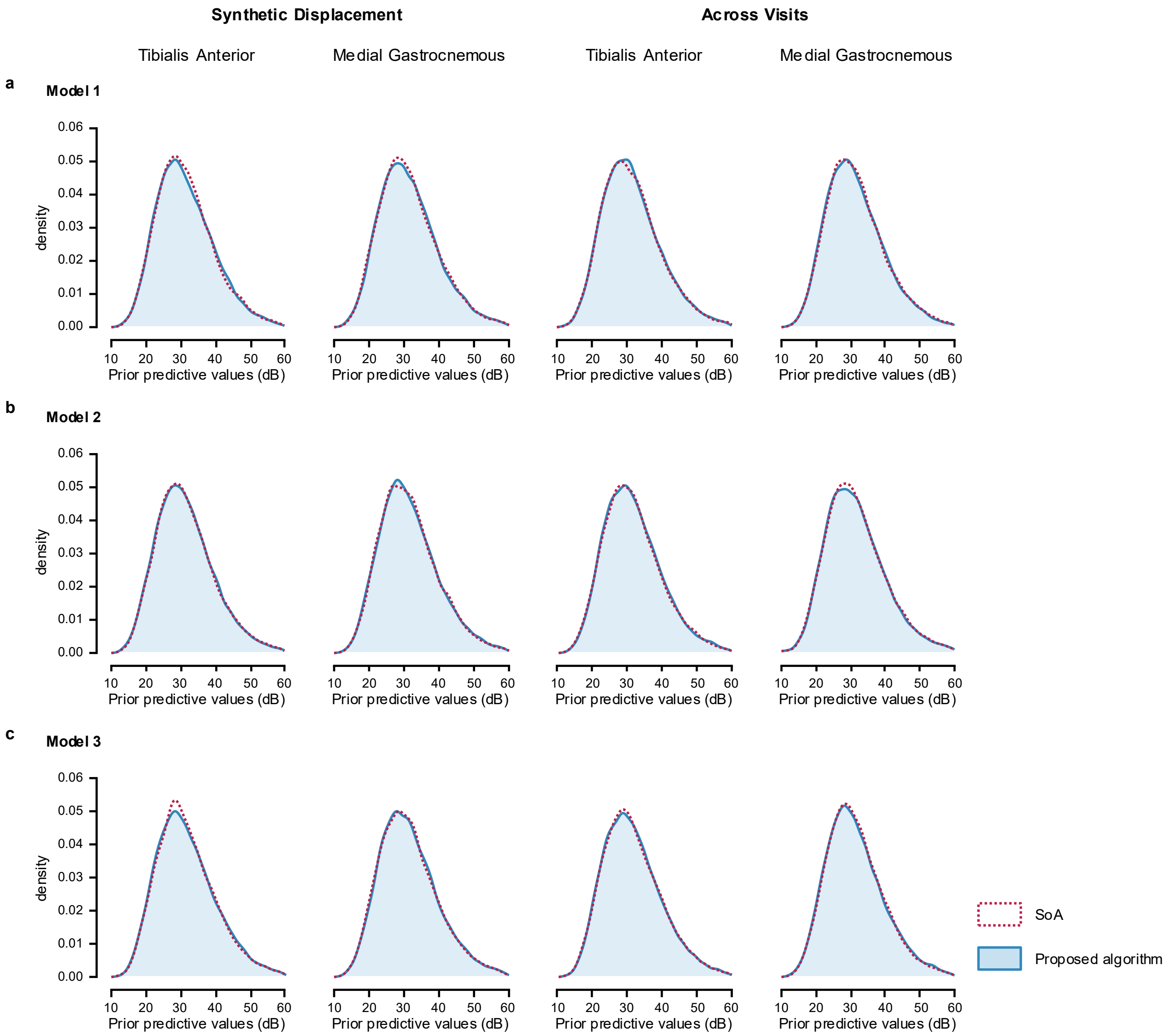

Supplementary Fig. 1: Prior predictive check for Bayesian comparison on PNR

Prior predictive checks for the Bayesian comparison of SoA and the proposed algorithm based on PNR of MU firing in tibialis anterior and medial gastrocnemius muscles, obtained by tracking MUs across synthetic displacement of electrode grid and two testing visits. Simulations were performed for **a**, Model 1; **b**, Model 2; and **c**, Model 3.

Based on visual inspection of data distribution, the sensitivity values were assumed to be independently sampled from a zero-one-inflated beta distribution.

$$y_i|\mu_i, \phi_i, \zeta_i, \kappa_i \sim \text{ZOIB}(\mu_i, \phi_i, \zeta_i, \kappa_i) \quad \text{(S27)}$$

with

$$\mu_i, \zeta_i, \kappa_i \in (0,1) \quad \text{(S28)}$$

and

$$\phi_i > 0 \quad \text{(S29)}$$

where

$$\text{ZOIB}(\mu_i, \phi_i, \zeta_i, \kappa_i) = \begin{cases} \zeta_i(1-\kappa_i) & , y_i = 0 \\ \zeta_i\kappa_i & , y_i = 1 \\ (1-\zeta_i)\ \text{Beta}(\mu_i\phi_i, (1-\mu_i)\phi_i) & , 0 < y_i < 1 \end{cases} \quad \text{(S30)}$$

and the parameter models

$$\text{logit}(\mu_i) = \alpha_\mu + \mathrm{x}_i{}^T\beta_\mu + b_{s_i} \quad \text{(S31)}$$

$$\log(\phi_i) = \alpha_\phi + \mathrm{x}_i{}^T\beta_\phi \quad \text{(S32)}$$

$$\text{logit}(\zeta_i) = \alpha_\zeta + \mathrm{x}_i{}^T\beta_\zeta \quad \text{(S33)}$$

$$\text{logit}(\kappa_i) = \alpha_\kappa + \mathrm{x}_i{}^T\beta_\kappa \quad \text{(S34)}$$

The sensitivity of MU i is denoted by y_i, μ_i denotes the beta component mean, ϕ_i is the beta precision parameter, with beta shape parameter $\mu_i\phi_i$ and $(1-\mu_i)\phi_i$, the zero-one inflation probability denotes by ζ_i, and κ_i is the conditional-one probability.

Priors for the mean submodel were

$$\alpha_\mu \sim \text{Normal}(0,1) \quad \text{(S35)}$$

$$\beta_{\mu,k} \sim \text{Normal}(0, 3^2),\ k = 1, \dots, K_\mu \quad \text{(S36)}$$

$$b_j|\tau_b \sim \text{Normal}(0, \tau_b{}^2),\ j = 1, \dots, J \quad \text{(S37)}$$

$$\tau_b \sim \text{Exponential}(1),\ \tau_b > 0 \quad \text{(S38)}$$

Here α_μ denotes population-level intercept for the mean submodel, $\beta_{\mu,k}$ denotes population-level method effects for coefficient k, $b_j|\tau_b$ denotes MUset-specific random intercepts conditional on the between-MUset standard deviation, and τ_b denotes between-MUset standard deviation.

Priors for the precision submodel were

$$\alpha_{\phi} \sim \text{Normal}(\log(20), 1) \quad \text{(S39)}$$

$$\beta_{\phi,k} \sim \text{Normal}(0, 3^2),\ k = 1, \ldots, K_{\phi} \quad \text{(S40)}$$

where α_{ϕ} denotes precision-model intercept and $\beta_{\phi,k}$ denotes the method effects in the precision submodel.

Priors for the zero-one inflation submodel were

$$\alpha_{\zeta} \sim \text{Normal}(0,1) \quad \text{(S41)}$$

$$\beta_{\zeta,k} \sim \text{Normal}(0, 3^2),\ k = 1, \ldots, K_{\zeta} \quad \text{(S42)}$$

where α_{ζ} denotes zero-one inflation intercept and $\beta_{\zeta,k}$ denotes the method effects in the zero-one inflation submodel.

Finally, priors for the conditional one-inflation submodel were

$$\alpha_{\kappa} \sim \text{Normal}(0,1) \quad \text{(S43)}$$

$$\beta_{\kappa,k} \sim \text{Normal}(0, 3^2),\ k = 1, \ldots, K_{\kappa} \quad \text{(S44)}$$

where α_{κ} denotes conditional one-inflation intercept and $\beta_{\kappa,k}$ denotes the method effects in the conditional one-inflation submodel.

Priors were specified to be weakly informative on the scale of the observed sensitivity and precision of the MU firing identification. These priors were evaluated using prior predictive simulations to confirm that the priors generated values within a plausible range (Supplementary Fig. 2a).

Model 2

In this model we additionally allowed the Method effect to vary by MUset by adding MUset-specific Method slopes. The model was implemented using the formula bf(Y ~ Method + (1 + Method | MUset), phi ~ Method, zoi ~ Method, coi ~ Method). Therefore

$$\text{logit}(\mu_i) = \alpha_{\mu} + \mathbf{x}_i{}^T \beta_{\mu} + \mathbf{r}_i{}^T \mathbf{b}_{s_i} \quad \text{(S45)}$$

where $\mathbf{r}_i$ is group-level design vector for observation i.

The likelihood and priors of Model 1 were used, except for $b_j|\tau_b \sim \text{Normal}(0, \tau_b{}^2)$ which was replaced with random intercept and random method effects defined as

$$\mathbf{b}_j \big| \tau_b, \mathbf{R} \sim \text{MultivariateNormal}\big(0, \text{diag}(\tau_b)\ \mathbf{R}\ \text{diag}(\tau_b)\big) \qquad \text{(S46)}$$

with

$$\mathbf{b}_j = \left(b_{0j}, b_{1j}, \ldots, b_{K_\mu j}\right)^{\mathrm{T}} \qquad \text{(S47)}$$

where $\mathbf{R}$ contains the correlation between the random intercept and the random method effects. Furthermore, we additionally assigned an LKJ prior to the correlation matrix among the MUset-specific intercept and Method effects, defined as

$$\mathbf{R} \sim \text{LKJ}(2) \qquad \text{(S48)}$$

Prior predictive simulations confirmed that the priors generated sensitivity values within a plausible range (Supplementary Fig. 2b).

<u>Model 3</u>

Compared to Model 2, this model additionally allowed observations from each MUset-MUID combination to have their own random intercept. The model was implemented using the formula bf(Y ~ Method + (1 + Method | MUset) + (1 | MUset:MUID), phi ~ Method, zoi ~ Method, coi ~ Method). Therefore,

$$\text{logit}(\mu_i) = \alpha_\mu + \mathbf{x}_i{}^T \beta_\mu + \mathbf{r}_i{}^T \mathbf{b}_{s_i} + c_{s_i l_i} \qquad \text{(S49)}$$

Here $c_{s_i l_i}$ denotes random intercept for the MUset-MUID combination associated with observation i.

The likelihood and priors of Model 2 were used in this model because Model 2 included a broad prior for the class-level random-effect standard deviation (class = “sd”). The prior predictive check is shown in (Supplementary Fig. 2c).

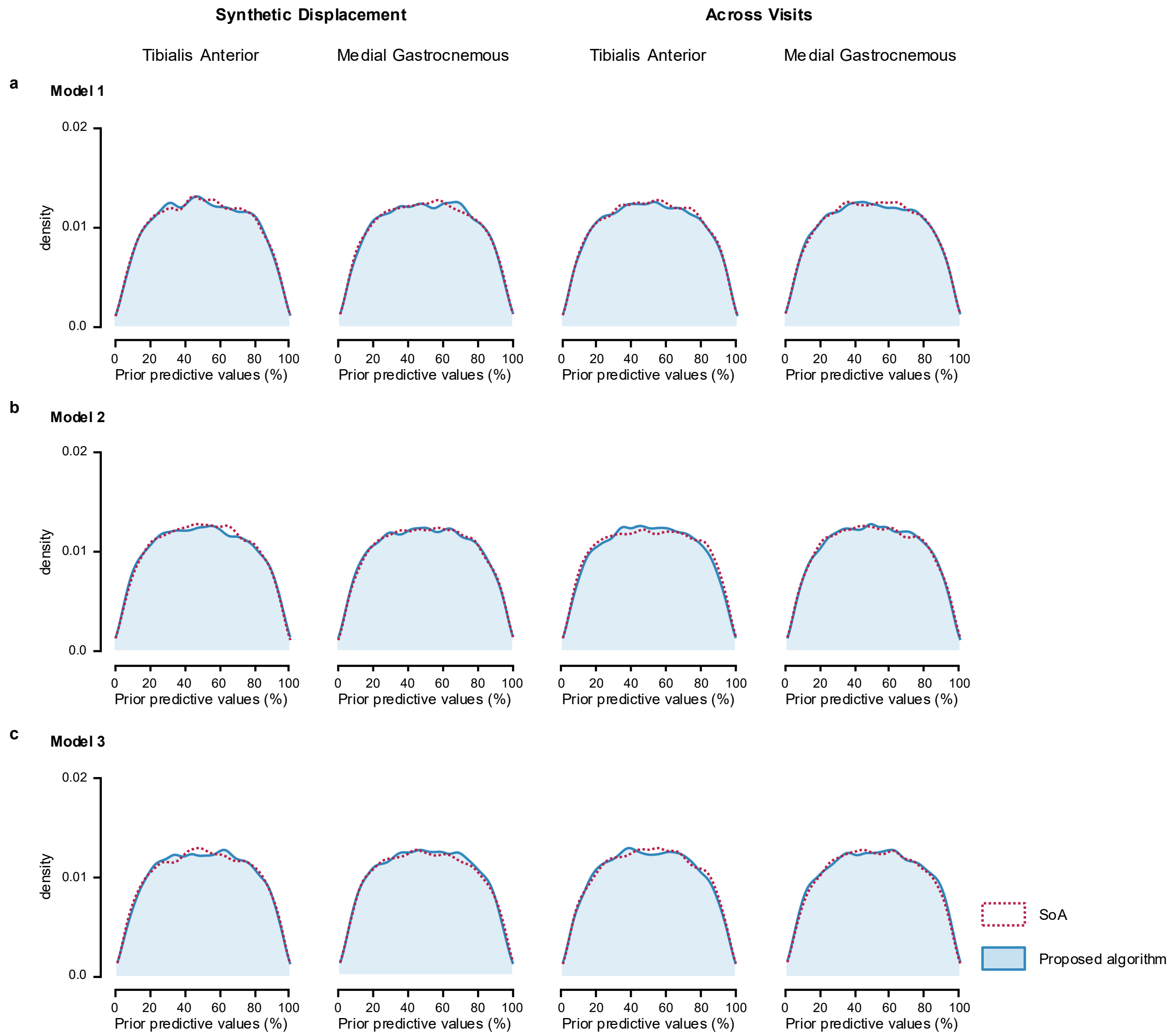

Supplementary Fig. 2: Prior predictive check for Bayesian comparison on sensitivity and precision of the MU firing identification

Prior predictive checks for the Bayesian comparison of SoA and the proposed algorithms on sensitivity and precision of the MU firing identification in tibialis anterior and medial gastrocnemius muscles, obtained by tracking MUs across synthetic displacement of electrode grid and two testing visits. Simulations were performed for **a**, Model 1; **b**, Model 2; and **c**, Model 3.

Supplementary Note 6. Comparison of firing identification PNR for tracked TA MUs across synthetic grid displacement.

Model implementation, likelihood function, and priors are detailed in Supplementary Note 5. The full dataset contains firing identification PNR values for 368 tracked MUs in the tibialis anterior (TA) muscle (Supplementary Fig. 3). The MUs firings were obtained by applying the MU filters calculated using the SoA algorithm[5] and the proposed algorithm to the HDsEMG signals generated with synthetic grid displacement.

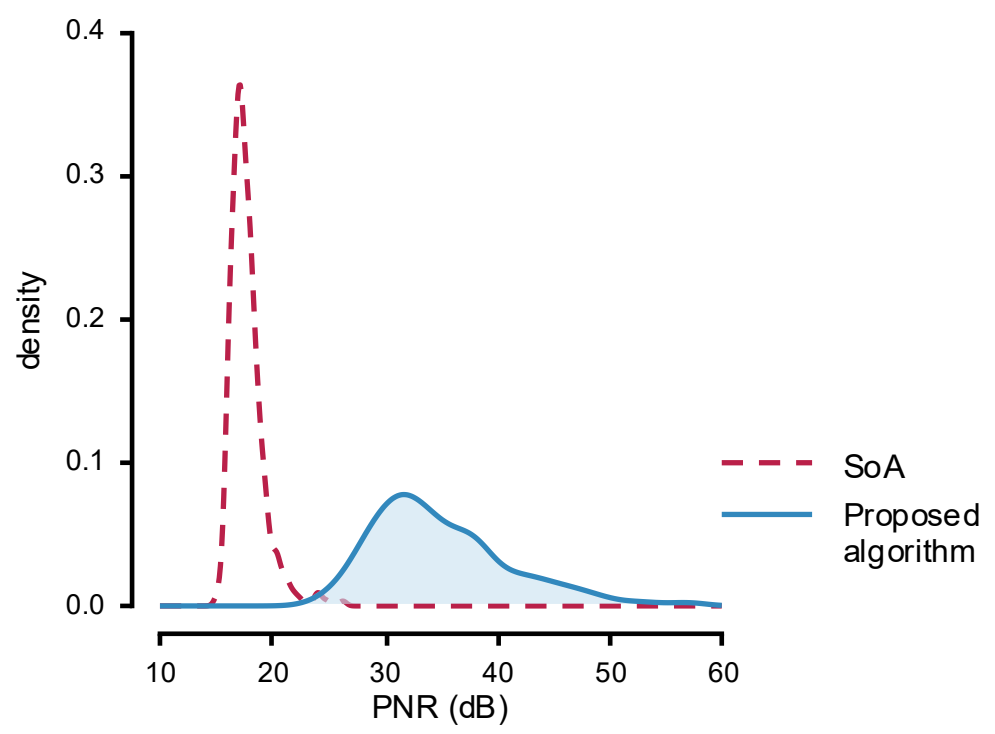

Supplementary Fig. 3: Distribution of firing identification PNR in tracked TA MUs across synthetic grid displacement.

PNR values were calculated from firing identification obtained by applying MU filters calculated using SoA (dashed line) and the proposed algorithms (solid line) to the HDsEMG signals generated with synthetic grid displacement.

In all models, the lognormal mean and sigma effects were exponentiated before the posterior summaries were calculated. Group-level standard deviations (SDs) are reported on their native model or link scales. Posterior diagnostics include the point estimate and upper confidence limit of the potential scale reduction factor (PSRF Pt and PSRF UpCI, respectively), as well as the effective sample size (ESS). Model selection was based on the difference in expected log pointwise predictive density (ELPD diff) and its standard error (SE diff).

Model 1

The maximum PSRF across all parameters was 1.001, indicating good convergence, while the minimum ESS was 6,327.809, indicating stable estimates of the bounds of the credible intervals (Supplementary Table 1). The medians, 95% equal-tailed interval (ETI) and 95% highest-density interval (HDI) are reported in Supplementary Table 2.

Supplementary Table 1: Posterior MCMC diagnostics for Model 1 of firing identification PNR in tracked TA MUs across synthetic grid displacement.

	PSRF Pt	PSRF UpCI	ESS
α_{μ}	1.000	1.001	6,598.152
α_{σ}	1.000	1.000	2,8463.848
$\beta_{\mu,\text{Proposed}}$	1.000	1.000	3,6178.124
$\beta_{\sigma,\text{Proposed}}$	1.000	1.000	2,9771.300
τ_b	1.000	1.001	6,327.809

Supplementary Table 2: Posterior summaries for Model 1 of firing identification PNR in tracked TA MUs across synthetic grid displacement.

	50%	95% ETI		Mode	95% HDI	
		2.5%	97.5%		low	high
α_{μ}	17.709	17.396	18.024	17.698	17.382	18.009
α_{σ}	1.952	1.918	1.987	1.953	1.918	1.987
$\beta_{\mu,\text{Proposed}}$	0.073	0.067	0.079	0.073	0.067	0.079
$\beta_{\sigma,\text{Proposed}}$	2.172	1.940	2.426	2.162	1.927	2.410
τ_b	0.041	0.029	0.058	0.041	0.028	0.057

Posterior predictive checks were performed by overlaying model-generated predictions on the observed data (Supplementary Fig. 4a). The posterior distributions (Supplementary Fig. 4b) indicated that the proposed algorithm achieved a higher PNR (35.0 dB, [34.2-35.8 dB]) than SoA algorithm (17.8 dB, [17.4-18.1 dB]). The posterior distribution of the difference excluded zero, and less than 2.5% of its distribution fell within the region of practical equivalence (ROPE).

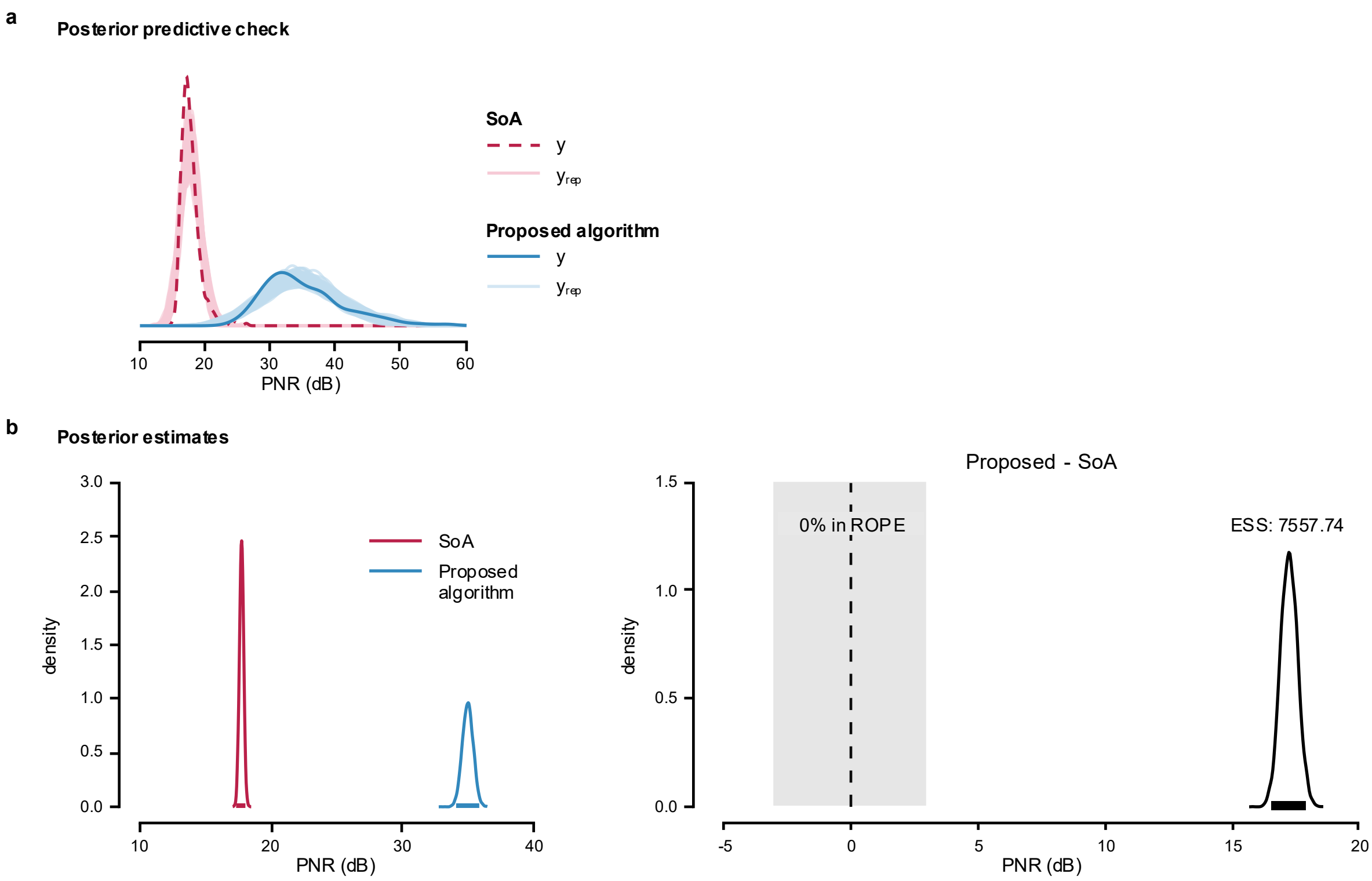

Supplementary Fig. 4: Posterior predictive check and posterior estimates for Model 1 of firing identification PNR in tracked TA MUs across synthetic grid displacement. **a**, Posterior predictive check of the model; **b**, Posterior distributions and the difference between means estimated using Bayesian Model 1 to compare the performance of the proposed algorithm and SoA method.

Model 2

The maximum PSRF across all parameters was 1.005, indicating good convergence, while the minimum ESS was 3,858.575, indicating stable estimates of the bounds of the credible intervals (Supplementary Table 3). The medians, 95% ETIs, and 95% HDIs are reported in Supplementary Table 4. Group-level correlations were retained on their native scales.

Posterior predictive checks were performed by overlaying model-generated predictions on the observed data (Supplementary Fig. 5a). The posterior distributions (Supplementary Fig. 5b) indicated that the proposed algorithm achieved a higher PNR (34.1 dB, [32.6-35.6 dB]) than the SoA algorithm (17.8 dB, [17.5-18.2 dB]). The posterior distribution of the difference excluded zero, and less than 2.5% of its distribution fell within ROPE.

Model 3

The maximum PSRF across all parameters was 1.001, indicating good convergence, while the minimum ESS was 2,771.133, indicating stable estimates of the bounds of the credible intervals (Supplementary Table 5). Posterior summaries are presented in Supplementary Table 6 as medians with 95% ETIs and 95% HDIs. Correlations were summarized on their native scales.

Posterior predictive checks were performed by overlaying model-generated predictions on the observed data (Supplementary Fig. 6a). The posterior distributions (Supplementary Fig. 6b) indicated that the proposed algorithm achieved a higher PNR (34.1 dB, [32.6-35.7 dB]) than the SoA algorithm (17.8 dB, [17.5-18.2 dB]). The posterior distribution of the difference excluded zero, and less than 2.5% of its distribution fell within ROPE.

Supplementary Table 3: Posterior MCMC diagnostics for Model 2 of firing identification PNR in tracked TA MUs across synthetic grid displacement.

	PSRF Pt	PSRF UpCI	ESS
α_μ	1.001	1.002	5,728.089
α_σ	1.000	1.000	2,0759.144
$\beta_{\mu,\text{Proposed}}$	1.000	1.001	4,421.218
$\beta_{\sigma,\text{Proposed}}$	1.000	1.001	2,1989.828
τ_{b0}	1.000	1.000	5,716.491
$\tau_{b\text{Proposed}}$	1.000	1.000	5,140.098
R_{12}	1.002	1.005	3,858.575

Supplementary Table 4: Posterior summaries for Model 2 of firing identification PNR in tracked TA MUs across synthetic grid displacement.

	50%	95% ETI		Mode	95% HDI	
		2.5%	97.5%		low	high
α_μ	17.776	17.455	18.112	17.783	17.451	18.107
α_σ	0.071	0.066	0.076	0.070	0.065	0.076
$\beta_{\mu,\text{Proposed}}$	1.903	1.813	1.989	1.907	1.814	1.989
$\beta_{\sigma,\text{Proposed}}$	1.908	1.719	2.123	1.896	1.707	2.110
τ_{b0}	0.043	0.030	0.063	0.041	0.029	0.061
$\tau_{b\text{Proposed}}$	0.112	0.081	0.158	0.109	0.079	0.154
R_{12}	-0.251	-0.606	0.177	-0.305	-0.621	0.158

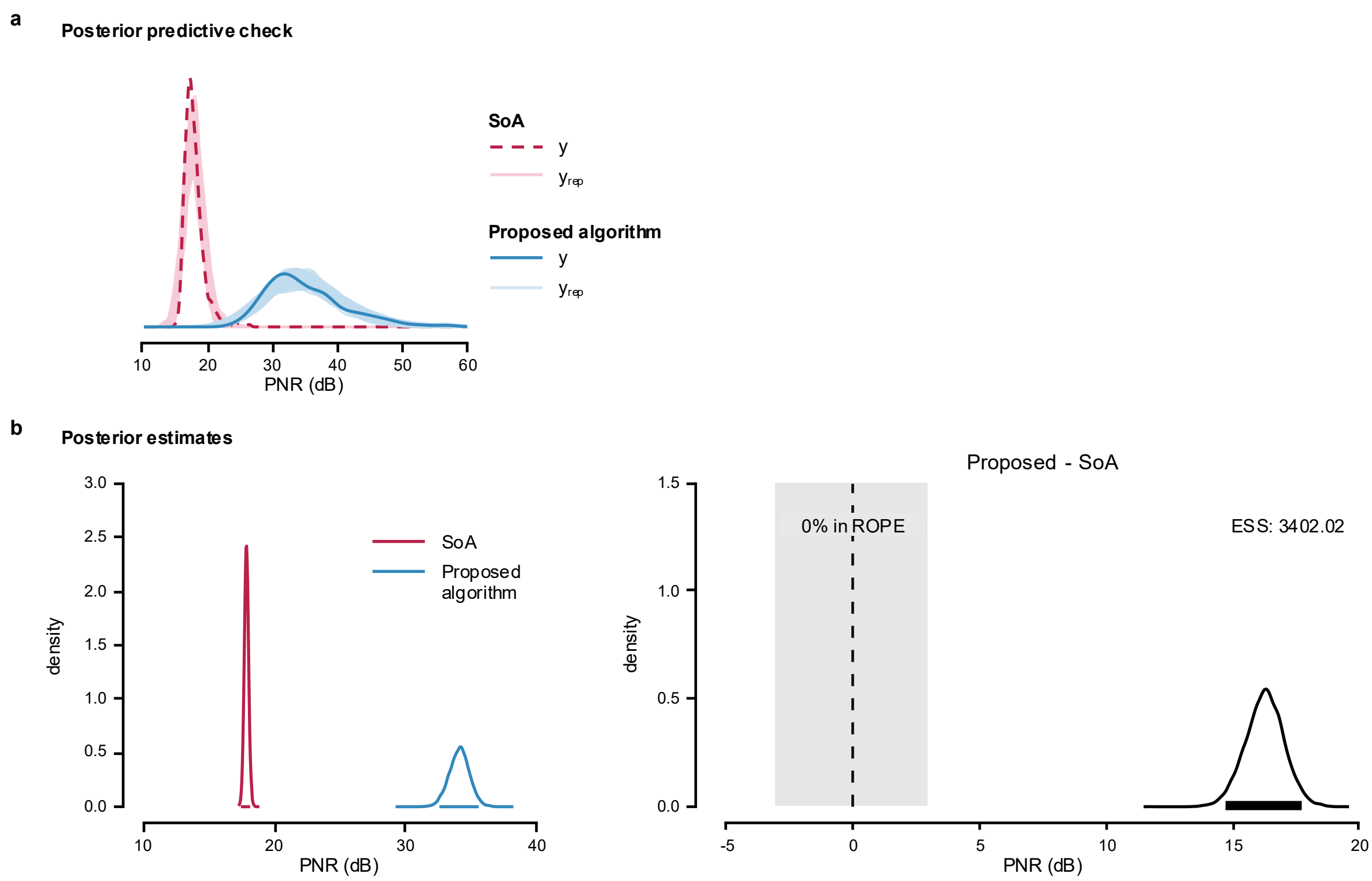

Supplementary Fig. 5: Posterior predictive check and posterior estimates for Model 2 of firing identification PNR in tracked TA MUs across synthetic grid displacement.

a, Posterior predictive check of the model; **b**, Posterior distributions and the difference in means from Bayesian Model 2, used to compare the performance of the proposed algorithm and SoA method.

Supplementary Table 5: Posterior MCMC diagnostics for Model 3 of firing identification PNR in tracked TA MUs across synthetic grid displacement.

	PSRF Pt	PSRF UpCI	ESS
α_{μ}	1.000	1.000	8,436.545
α_{σ}	1.000	1.001	6,749.176
$\beta_{\mu,\text{Proposed}}$	1.000	1.000	5,977.018
$\beta_{\sigma,\text{Proposed}}$	1.000	1.001	1,3898.05
τ_{b0}	1.000	1.000	8,343.782
$\tau_{b\text{Proposed}}$	1.000	1.000	6,636.922
τ_{c}	1.000	1.001	2,771.133
R_{12}	1.000	1.001	5,044.315

Supplementary Table 6: Posterior summaries for Model 3 of firing identification PNR in tracked TA MUs across synthetic grid displacement.

	50%	95% ETI		Mode	95% HDI	
		2.5%	97.5%		low	high
α_{μ}	17.771	17.446	18.112	17.765	17.447	18.113
α_{σ}	0.069	0.063	0.076	0.069	0.063	0.076
$\beta_{\mu,\text{Proposed}}$	1.903	1.816	1.989	1.904	1.815	1.989
$\beta_{\sigma,\text{Proposed}}$	1.938	1.739	2.182	1.915	1.733	2.174
τ_{b0}	0.043	0.030	0.062	0.042	0.029	0.060
$\tau_{b\text{Proposed}}$	0.112	0.082	0.156	0.109	0.077	0.151
τ_{c}	0.012	0.001	0.031	0.005	0.000	0.028
R_{12}	-0.259	-0.607	0.183	-0.303	-0.628	0.154

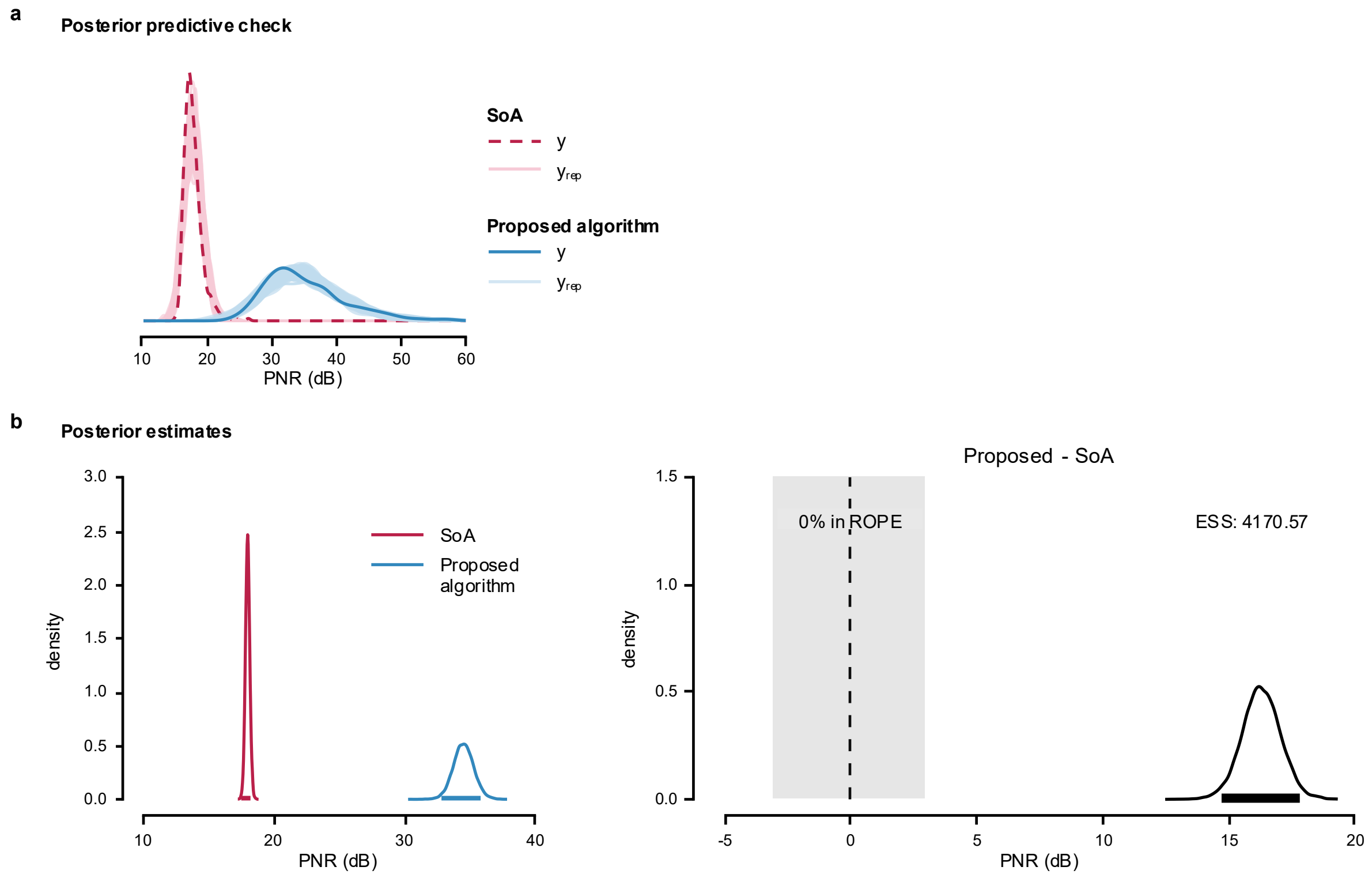

Supplementary Fig. 6: Posterior predictive check and posterior estimates for Model 3 of firing identification PNR in tracked TA MUs across synthetic grid displacement.

a, Posterior predictive check of the model; **b**, Posterior distributions and the difference in means from Bayesian Model 3, used to compare the performance of the proposed algorithm and SoA method.

MODEL COMPARISON

Model 2 yielded the highest expected predictive accuracy. Relative to Model 2, the ELPD diff was -0.3 (SE = 0.7) for Model 3 and -57.1 (SE = 12.5) for Model 1, indicating comparable performance for Models 2 and 3 but poorer performance for Model 1.

Supplementary Note 7. Comparison of firing identification sensitivity in tracked TA MUs across synthetic grid displacement.

Model implementation, likelihood function, and priors are detailed in Supplementary Note 5. The full dataset contains firing identification sensitivity values for 368 tracked TA MUs (hereafter referred to as 'sensitivity'). The MUs firings were obtained by applying the MU filters calculated using the SoA algorithm[5] and the proposed algorithm to the HDsEMG signals generated with synthetic grid displacement. Distribution of the data is shown in Supplementary Fig. 7.

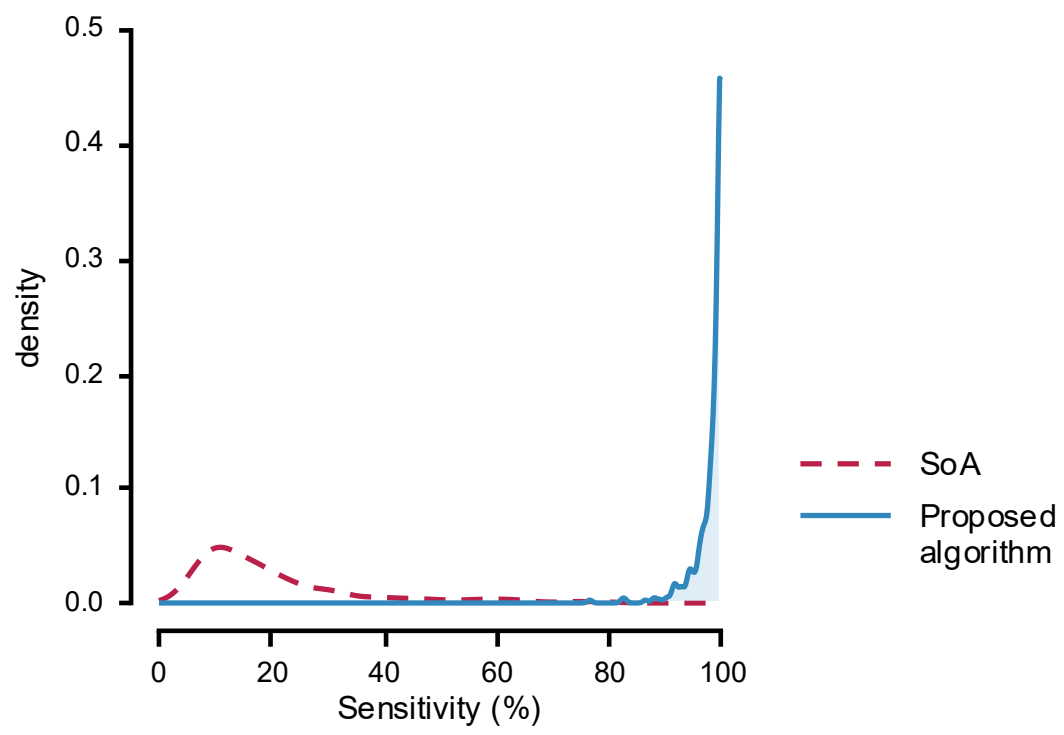

Supplementary Fig. 7: Distribution of firing identification sensitivity in tracked TA MUs across synthetic grid displacement.

Sensitivity values were calculated from firing identification obtained by applying MU filters calculated using SoA (dashed line) and the proposed algorithms (solid line) to the HDsEMG signals generated with synthetic grid displacement.

In all models, ZOIB parameters were back transformed into their interpretable component scales: *mu*, *zoi*, and *coi* to probabilities, and *phi* to the positive precision scale. Group-level SDs are reported on their native model/link scale.

Model 1

The maximum PSRF across all parameters was 1.001, indicating good convergence, while the minimum ESS was 5,390.882, indicating stable estimates of the bounds of the credible intervals (Supplementary Table 7). Posterior summaries are reported in Supplementary Table 8 as medians with 95% ETIs and 95% HDIs.

Posterior predictive checks were performed by overlaying model-generated predictions on the observed data (Supplementary Fig. 8a). The posterior distributions (Supplementary Fig. 8b) indicated that the proposed algorithm achieved a higher sensitivity (98.0%, [97.2-98.5 %]) than the SoA algorithm (20.9%, [18.6-23.3%]). The posterior distribution of the difference excluded zero, and less than 2.5% of its distribution fell within ROPE.

Supplementary Table 7: Posterior MCMC diagnostics for Model 1 of firing identification sensitivity in tracked TA MUs across synthetic grid displacement.

	PSRF Pt	PSRF UpCI	ESS
α_{μ}	1.000	1.000	5,390.882
α_{ϕ}	1.000	1.000	1,9961.068
α_{ζ}	1.000	1.001	9,074.187
α_{κ}	1.000	1.000	1,4038.931
$\beta_{\mu,\text{Proposed}}$	1.000	1.000	1,5246.186
$\beta_{\phi,\text{Proposed}}$	1.000	1.001	1,4830.966
$\beta_{\zeta,\text{Proposed}}$	1.000	1.001	9,149.810
$\beta_{\kappa,\text{Proposed}}$	1.000	1.000	1,4782.282
τ_{b}	1.000	1.001	5,796.351

Supplementary Table 8: Posterior summaries for Model 1 of firing identification sensitivity in tracked TA MUs across synthetic grid displacement.

	50%	95% ETI		Mode	95% HDI	
		2.5%	97.5%		low	high
α_μ	0.209	0.186	0.233	0.209	0.186	0.233
α_ϕ	10.480	8.892	12.226	10.297	8.812	12.137
α_ζ	0.005	0.001	0.014	0.003	0.000	0.012
α_κ	0.136	0.006	0.816	0.035	0.001	0.715
$\beta_{\mu,\text{Proposed}}$	136.590	117.520	158.554	135.365	117.269	158.172
$\beta_{\phi,\text{Proposed}}$	3.903	2.956	5.145	3.882	2.867	5.034
$\beta_{\zeta,\text{Proposed}}$	154.881	49.605	808.805	91.756	26.332	592.249
$\beta_{\kappa,\text{Proposed}}$	819.936	17.966	4,7147.870	3,725.074	0.763	2,4486.400
τ_b	0.303	0.189	0.453	0.306	0.180	0.441

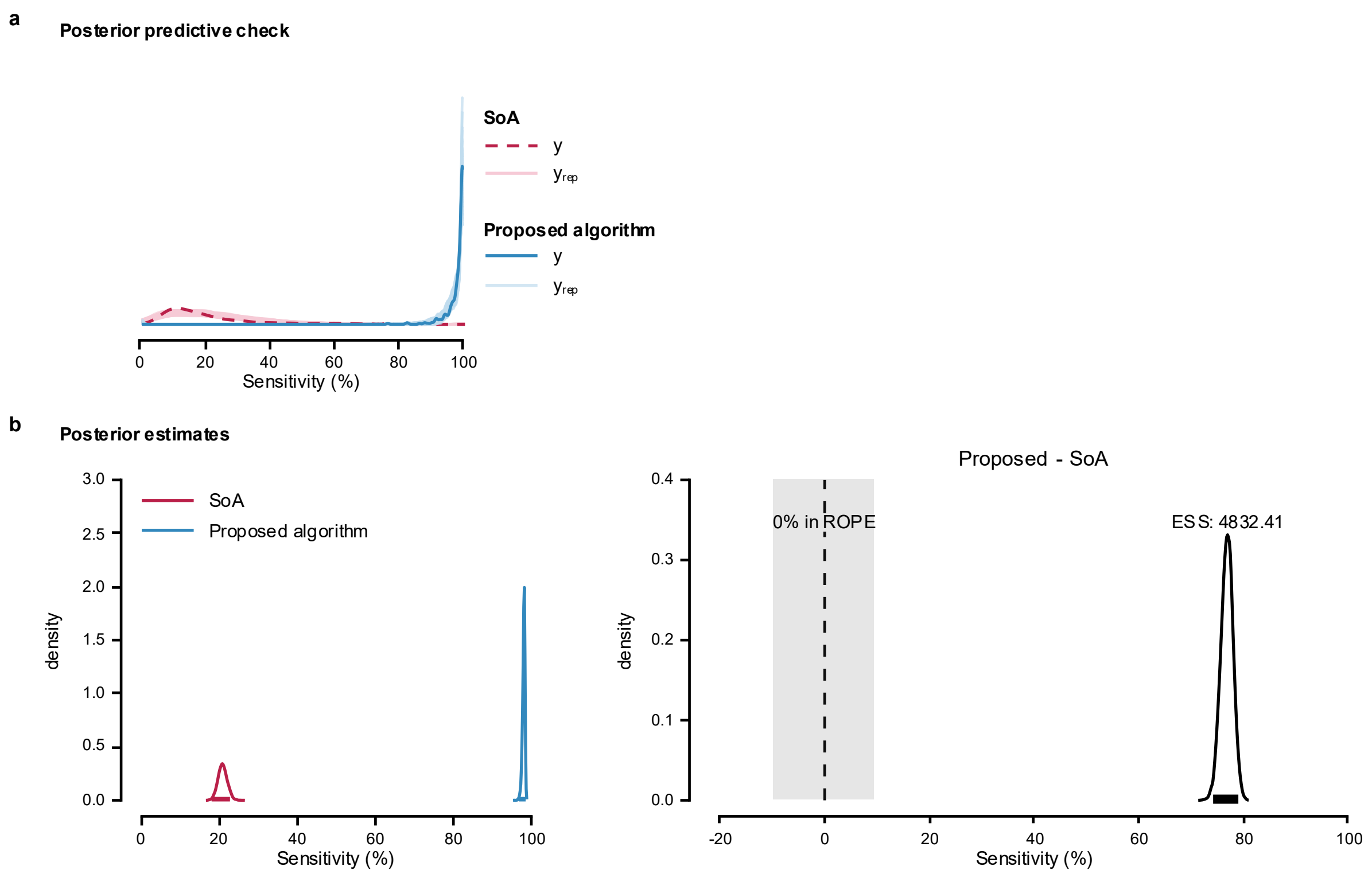

Supplementary Fig. 8: Posterior predictive check and posterior estimates for Model 1 of firing identification sensitivity in tracked TA MUs across synthetic grid displacement. **a**, Posterior predictive check of the model; **b**, Posterior distributions and the difference between means estimated using Bayesian Model 1 to compare the performance of the proposed algorithm and SoA method.

<u>Model 2</u>

The maximum PSRF across all parameters was 1.002, indicating good convergence, while the minimum ESS was 5,153.011, indicating stable estimates of the bounds of the credible intervals (Supplementary Table 9). Medians with 95% ETIs and 95% HDIs are presented in Supplementary Table 10. Group-level correlations are reported on the correlation scale.

Posterior predictive checks were performed by overlaying model-generated predictions on the observed data (Supplementary Fig. 9a). The posterior distributions (Supplementary Fig. 9b) indicated that the proposed algorithm achieved a higher sensitivity (97.9%, [97.1-98.4%])

than the SoA algorithm (21.2%, [18.5-24.2%]). The posterior distribution of the difference excluded zero, and less than 2.5% of its distribution fell within ROPE.

Supplementary Table 9: Posterior MCMC diagnostics for Model 2 of firing identification sensitivity in tracked TA MUs across synthetic grid displacement.

	PSRF Pt	PSRF UpCI	ESS
α_{μ}	1.000	1.001	5,153.011
α_{ϕ}	1.000	1.000	2,4832.207
α_{ζ}	1.001	1.002	1,1001.276
α_{κ}	1.000	1.001	1,4437.182
$\beta_{\mu,\text{Proposed}}$	1.001	1.002	6,605.306
$\beta_{\phi,\text{Proposed}}$	1.000	1.001	1,7148.260
$\beta_{\zeta,\text{Proposed}}$	1.001	1.002	1,1093.090
$\beta_{\kappa,\text{Proposed}}$	1.000	1.001	1,5091.433
τ_{b0}	1.000	1.001	5,283.492
$\tau_{b\text{Proposed}}$	1.000	1.000	6,925.981
R_{12}	1.000	1.001	9,115.252

Supplementary Table 10: Posterior summaries for Model 2 of firing identification sensitivity in tracked TA MUs across synthetic grid displacement.

	50%	95% ETI		Mode	95% HDI	
		2.5%	97.5%		low	high
α_{μ}	0.212	0.184	0.242	0.212	0.183	0.241
α_{ϕ}	11.389	9.768	13.208	11.162	9.671	13.106
α_{ζ}	0.005	0.001	0.014	0.003	0.000	0.012
α_{κ}	0.132	0.005	0.808	0.035	0.000	0.713
$\beta_{\mu,\text{Proposed}}$	125.267	97.696	157.976	124.716	96.548	156.372
$\beta_{\phi,\text{Proposed}}$	3.870	2.965	4.989	3.860	2.909	4.918
$\beta_{\zeta,\text{Proposed}}$	155.744	48.843	823.357	106.188	26.608	608.646
$\beta_{\kappa,\text{Proposed}}$	849.763	18.440	5,1723.880	-859.945	0.506	2,6485.510
τ_{b0}	0.404	0.288	0.567	0.392	0.277	0.551
$\tau_{b\text{Proposed}}$	0.486	0.301	0.720	0.463	0.285	0.700
R_{12}	-0.910	-0.986	-0.665	-0.944	-0.997	-0.720

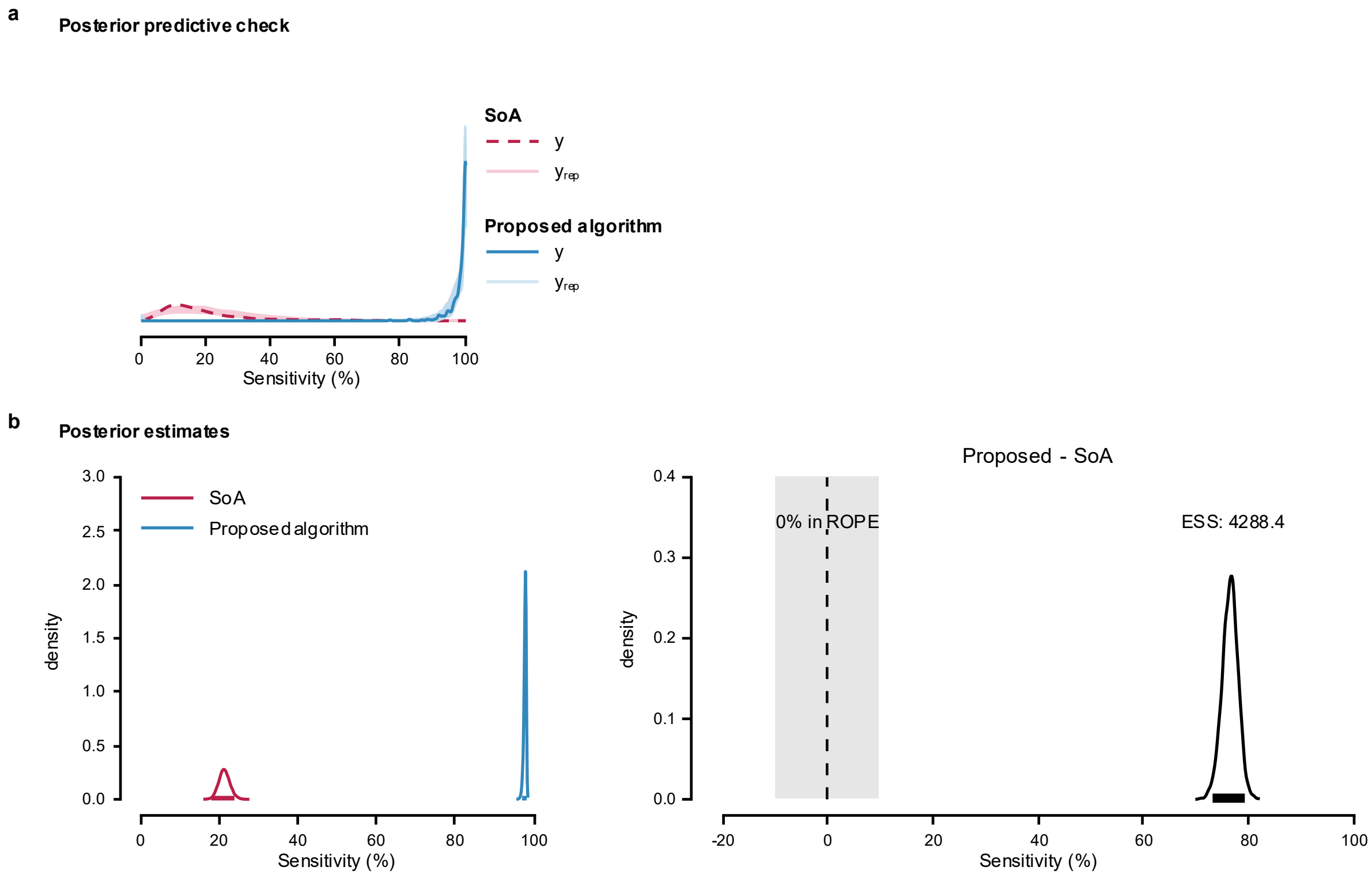

Supplementary Fig. 9: Posterior predictive check and posterior estimates for Model 2 of firing identification sensitivity in tracked TA MUs across synthetic grid displacement. **a**, Posterior predictive check of the model; **b**, Posterior distributions and the difference in means from Bayesian Model 2, used to compare the performance of the proposed algorithm and SoA method.

<u>Model 3</u>

The maximum PSRF across all parameters was 1.030, indicating good convergence, while the minimum ESS was 2,051.832, indicating stable estimates of the bounds of the credible intervals (Supplementary Table 11). Posterior estimates are reported in Supplementary Table 12 as medians with 95% ETIs and 95% HDIs. Correlations were summarized on their native scales.

Posterior predictive checks were performed by overlaying model-generated predictions on the observed data (Supplementary Fig. 10a). The posterior distributions (Supplementary Fig.

10b) indicated that the proposed algorithm achieved a higher sensitivity (97.9%, [97.1-98.4%]) than the SoA algorithm (21.2%, [18.2-24.3%]). The posterior distribution of the difference excluded zero, and less than 2.5% of its distribution fell within ROPE.

Supplementary Table 11: Posterior MCMC diagnostics for Model 3 of firing identification sensitivity in tracked TA MUs across synthetic grid displacement.

	PSRF Pt	PSRF UpCI	ESS
α_{μ}	1.000	1.001	4,911.977
α_{ϕ}	1.012	1.021	4,141.927
α_{ζ}	1.000	1.000	1,2040.026
α_{κ}	1.000	1.000	2,0798.665
$\beta_{\mu,\text{Proposed}}$	1.000	1.001	6,849.684
$\beta_{\phi,\text{Proposed}}$	1.002	1.003	1,4848.922
$\beta_{\zeta,\text{Proposed}}$	1.000	1.000	1,2402.239
$\beta_{\kappa,\text{Proposed}}$	1.000	1.000	2,1512.980
τ_{b0}	1.001	1.003	6,115.368
$\tau_{b\text{Proposed}}$	1.001	1.002	7,745.286
τ_{c}	1.014	1.030	2,051.832
R_{12}	1.000	1.000	9,786.335

Supplementary Table 12: Posterior summaries for Model 3 of firing identification sensitivity in tracked TA MUs across synthetic grid displacement.

	50%	95% ETI		Mode	95% HDI	
		2.5%	97.5%		low	high
α_{μ}	0.211	0.182	0.243	0.211	0.181	0.241
α_{ϕ}	11.694	9.924	14.578	11.584	9.667	14.102
α_{ζ}	0.005	0.001	0.014	0.003	0.000	0.012
α_{κ}	0.129	0.006	0.799	0.035	0.000	0.705
$\beta_{\mu,\text{Proposed}}$	126.414	98.098	161.830	125.433	97.551	161.046
$\beta_{\phi,\text{Proposed}}$	3.821	2.876	4.959	3.833	2.830	4.886
$\beta_{\zeta,\text{Proposed}}$	154.619	49.252	781.366	96.331	32.067	583.902
$\beta_{\kappa,\text{Proposed}}$	872.646	19.123	5,0303.580	2,539.043	0.507	2,5354.570
τ_{b0}	0.410	0.292	0.577	0.406	0.281	0.560
$\tau_{b\text{Proposed}}$	0.497	0.314	0.733	0.476	0.314	0.731
τ_{c}	0.074	0.003	0.275	0.019	0.000	0.234
R_{12}	-0.910	-0.987	-0.664	-0.944	-0.997	-0.723

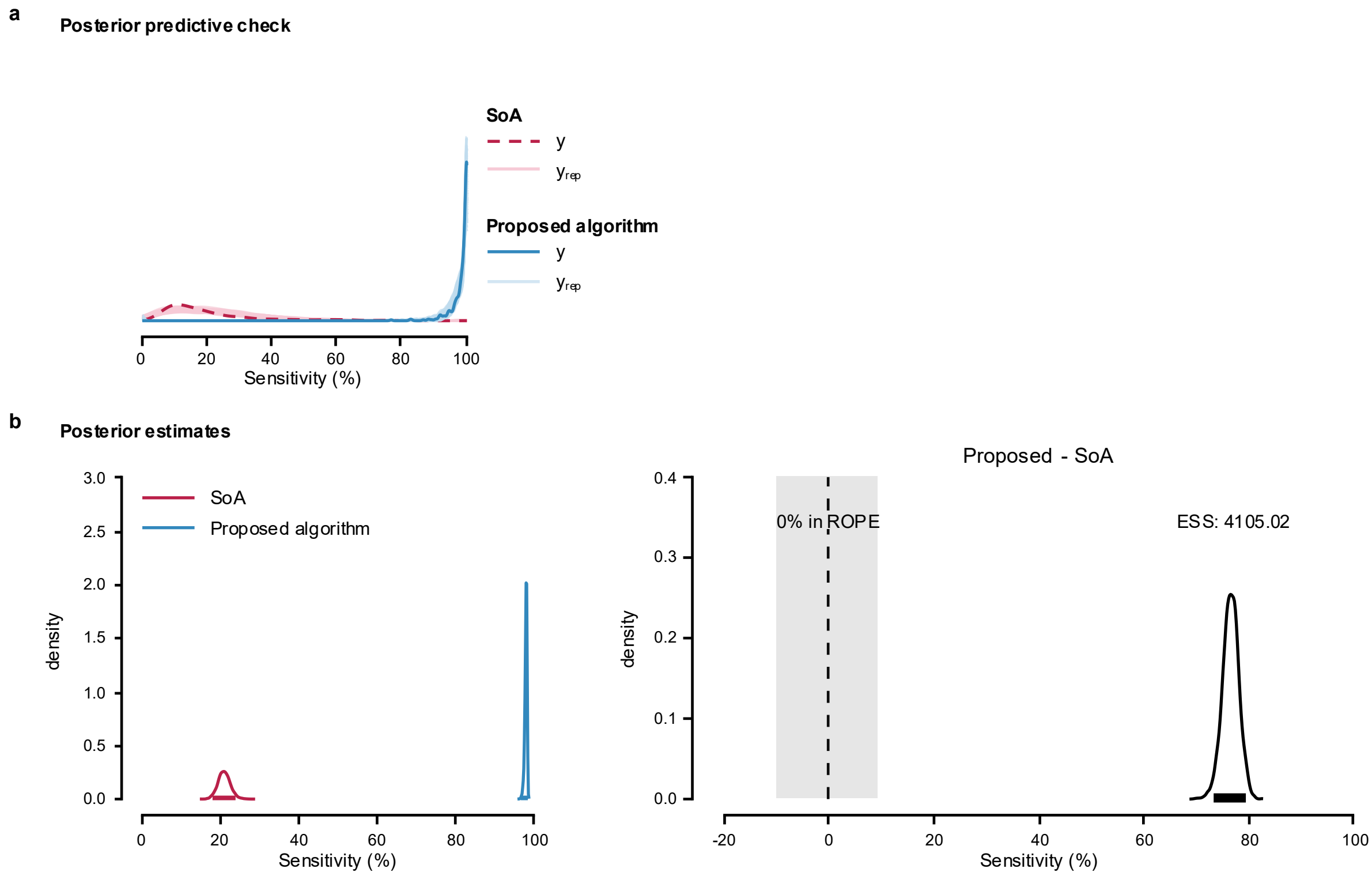

Supplementary Fig. 10: Posterior predictive check and posterior estimates for Model 3 of firing identification sensitivity in tracked TA MUs across synthetic grid displacement.

a, Posterior predictive check of the model; **b**, Posterior distributions and the difference in means from Bayesian Model 3, used to compare the performance of the proposed algorithm and SoA method.

MODEL COMPARISON

Model 2 showed the greatest expected predictive accuracy. ELPD diff relative to Model 2 were −0.5 (SE = 0.5) for Model 3 and −18.1 (SE = 5.5) for Model 1, suggesting similar predictive performance for Models 2 and 3 and reduced performance for Model 1.

Supplementary Note 8. Comparison of firing identification precision in tracked TA MUs across synthetic grid displacement.

Model implementation, likelihood function, and priors are detailed in Supplementary Note 5. The full dataset contains firing identification precision values for 368 tracked TA MUs (hereafter referred to as 'precision'). The MUs firings were obtained by applying the MU filters calculated using the SoA algorithm[5] and the proposed algorithm to the HDsEMG signals generated with synthetic grid displacement. Distribution of the data is shown in Supplementary Fig. 11.

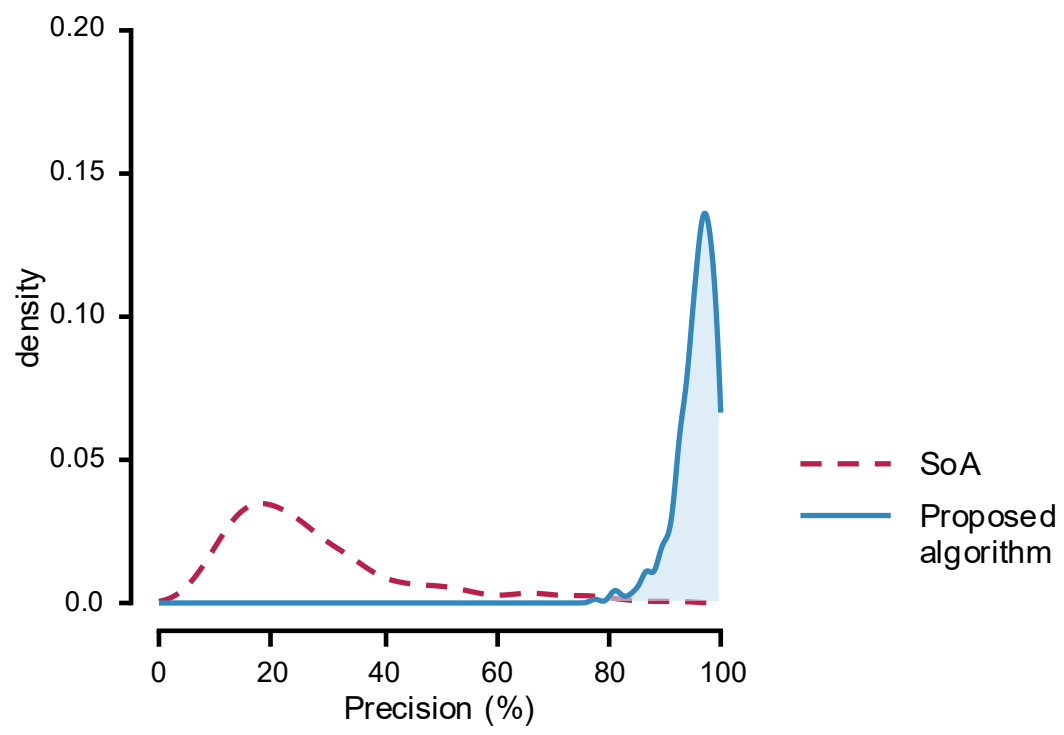

Supplementary Fig. 11: Distribution of firing identification precision in tracked TA MUs across synthetic grid displacement.

Precision values were calculated from firing identification obtained by applying MU filters calculated using SoA (dashed line) and the proposed algorithms (solid line) to the HDsEMG signals generated with synthetic grid displacement.

In all models, ZOIB parameters were back transformed into their interpretable component scales: *mu*, *zoi*, and *coi* to probabilities, and *phi* to the positive precision scale. Group-level SDs are reported on their native model/link scale.

Model 1

The maximum PSRF across all parameters was 1.002, indicating good convergence, while the minimum ESS was 6,125.286, indicating stable estimates of the bounds of the credible intervals (Supplementary Table 13). Supplementary Table 14 summarizes posterior estimates as medians with 95% ETIs and 95% HDIs.

Posterior predictive checks were performed by overlaying model-generated predictions on the observed data (Supplementary Fig. 12a). The posterior distributions (Supplementary Fig. 12b) indicated that the proposed algorithm achieved a higher precision (95.3%, [94.5-96.0 %]) than the SoA algorithm (29.0%, [26.5-31.6%]). The posterior distribution of the difference excluded zero, and less than 2.5% of its distribution fell within ROPE.

Supplementary Table 13: Posterior MCMC diagnostics for Model 1 of firing identification precision in tracked TA MUs across synthetic grid displacement.

	PSRF Pt	PSRF UpCI	ESS
α_{μ}	1.000	1.000	1,0695.240
α_{ϕ}	1.000	1.000	2,1607.562
α_{ζ}	1.000	1.001	2,0201.825
α_{κ}	1.000	1.000	2,6771.201
$\beta_{\mu,\text{Proposed}}$	1.000	1.000	2,8566.824
$\beta_{\phi,\text{Proposed}}$	1.000	1.000	2,4113.835
$\beta_{\zeta,\text{Proposed}}$	1.000	1.000	2,0145.884
$\beta_{\kappa,\text{Proposed}}$	1.000	1.000	2,5032.027
τ_{b}	1.001	1.002	6,125.286

Supplementary Table 14: Posterior summaries for Model 1 of firing identification precision in tracked TA MUs across synthetic grid displacement.

	50%	95% ETI		Mode	95% HDI	
		2.5%	97.5%		low	high
α_{μ}	0.290	0.266	0.316	0.291	0.265	0.315
α_{ϕ}	8.211	7.012	9.557	8.121	7.004	9.543
α_{ζ}	0.005	0.001	0.016	0.004	0.001	0.014
α_{κ}	0.214	0.010	0.880	0.049	0.000	0.813
$\beta_{\mu,\text{Proposed}}$	49.916	44.774	55.675	49.997	44.801	55.701
$\beta_{\phi,\text{Proposed}}$	4.902	3.896	6.152	4.891	3.799	6.030
$\beta_{\zeta,\text{Proposed}}$	13.889	4.277	66.407	8.683	2.290	50.842
$\beta_{\kappa,\text{Proposed}}$	109.270	2.046	7,927.556	-122.207	0.030	3,782.908
τ_{b}	0.240	0.143	0.371	0.230	0.136	0.360

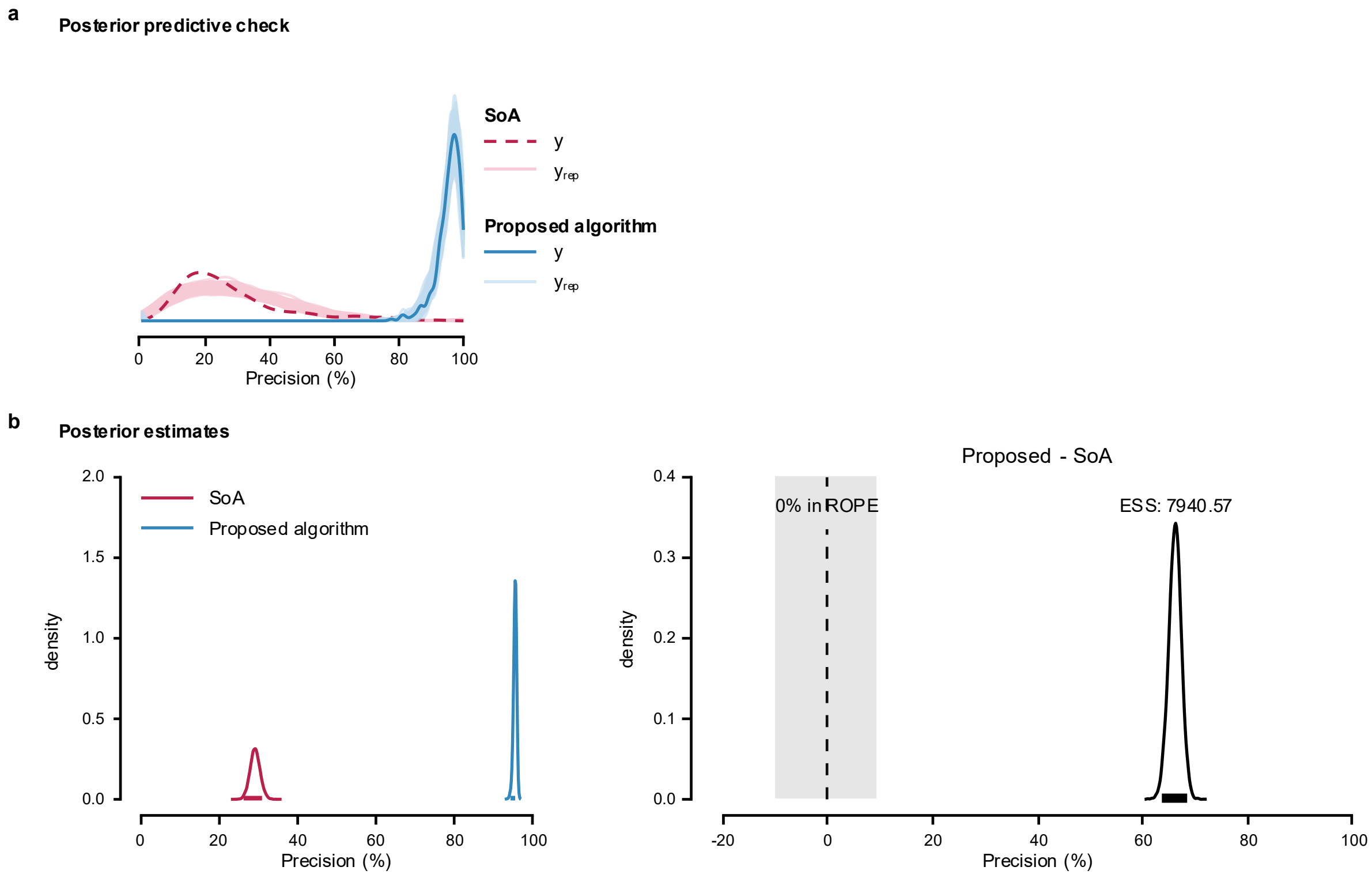

Supplementary Fig. 12: Posterior predictive check and posterior estimates for Model 1 of firing identification precision in tracked TA MUs across synthetic grid displacement. **a**, Posterior predictive check of the model; **b**, Posterior distributions and the difference between means estimated using Bayesian Model 1 to compare the performance of the proposed algorithm and SoA method.

<u>Model 2</u>

The maximum PSRF across all parameters was 1.003, indicating good convergence, while the minimum ESS was 4,034.166, indicating stable estimates of the bounds of the credible intervals (Supplementary Table 15). Posterior summaries are reported as medians with 95% ETIs and 95% HDIs on the original or component-specific interpretable scale (Supplementary Table 16). Group-level correlations are reported on the correlation scale.

Posterior predictive checks were performed by overlaying model-generated predictions on the observed data (Supplementary Fig. 13a). The posterior distributions (Supplementary Fig.

13b) indicated that the proposed algorithm achieved a higher precision (95.2%, [94.4-95.8%]) than the SoA algorithm (29.6%, [26.1-33.2%]). The posterior distribution of the difference excluded zero, and less than 2.5% of its distribution fell within ROPE.

Supplementary Table 15: Posterior MCMC diagnostics for Model 2 of firing identification precision in tracked TA MUs across synthetic grid displacement.

	PSRF Pt	PSRF UpCI	ESS
α_{μ}	1.001	1.003	4,034.166
α_{ϕ}	1.000	1.000	2,0916.869
α_{ζ}	1.000	1.001	1,1142.008
α_{κ}	1.000	1.000	1,5214.434
$\beta_{\mu,\text{Proposed}}$	1.000	1.001	5,560.573
$\beta_{\phi,\text{Proposed}}$	1.000	1.000	2,1036.767
$\beta_{\zeta,\text{Proposed}}$	1.000	1.001	1,1210.422
$\beta_{\kappa,\text{Proposed}}$	1.000	1.000	1,7114.483
τ_{b0}	1.001	1.003	5,221.211
$\tau_{b\text{Proposed}}$	1.000	1.001	6,010.290
R_{12}	1.001	1.002	7,980.684

Supplementary Table 16: Posterior summaries for Model 2 of firing identification precision in tracked TA MUs across synthetic grid displacement.

	50%	95% ETI		Mode	95% HDI	
		2.5%	97.5%		low	high
α_{μ}	0.295	0.262	0.332	0.294	0.262	0.332
α_{ϕ}	9.262	7.962	10.712	9.213	7.938	10.679
α_{ζ}	0.005	0.001	0.016	0.004	0.001	0.014
α_{κ}	0.208	0.010	0.876	0.049	0.001	0.810
$\beta_{\mu,\text{Proposed}}$	47.337	39.257	56.773	47.143	39.068	56.558
$\beta_{\phi,\text{Proposed}}$	4.468	3.588	5.557	4.439	3.505	5.446
$\beta_{\zeta,\text{Proposed}}$	13.819	4.239	68.057	10.173	2.332	51.009
$\beta_{\kappa,\text{Proposed}}$	110.880	2.127	8,030.003	-118.164	0.081	3,843.218
τ_{b0}	0.400	0.278	0.565	0.394	0.267	0.549
$\tau_{b\text{Proposed}}$	0.385	0.235	0.575	0.375	0.232	0.569
R_{12}	-0.877	-0.974	-0.621	-0.916	-0.987	-0.668

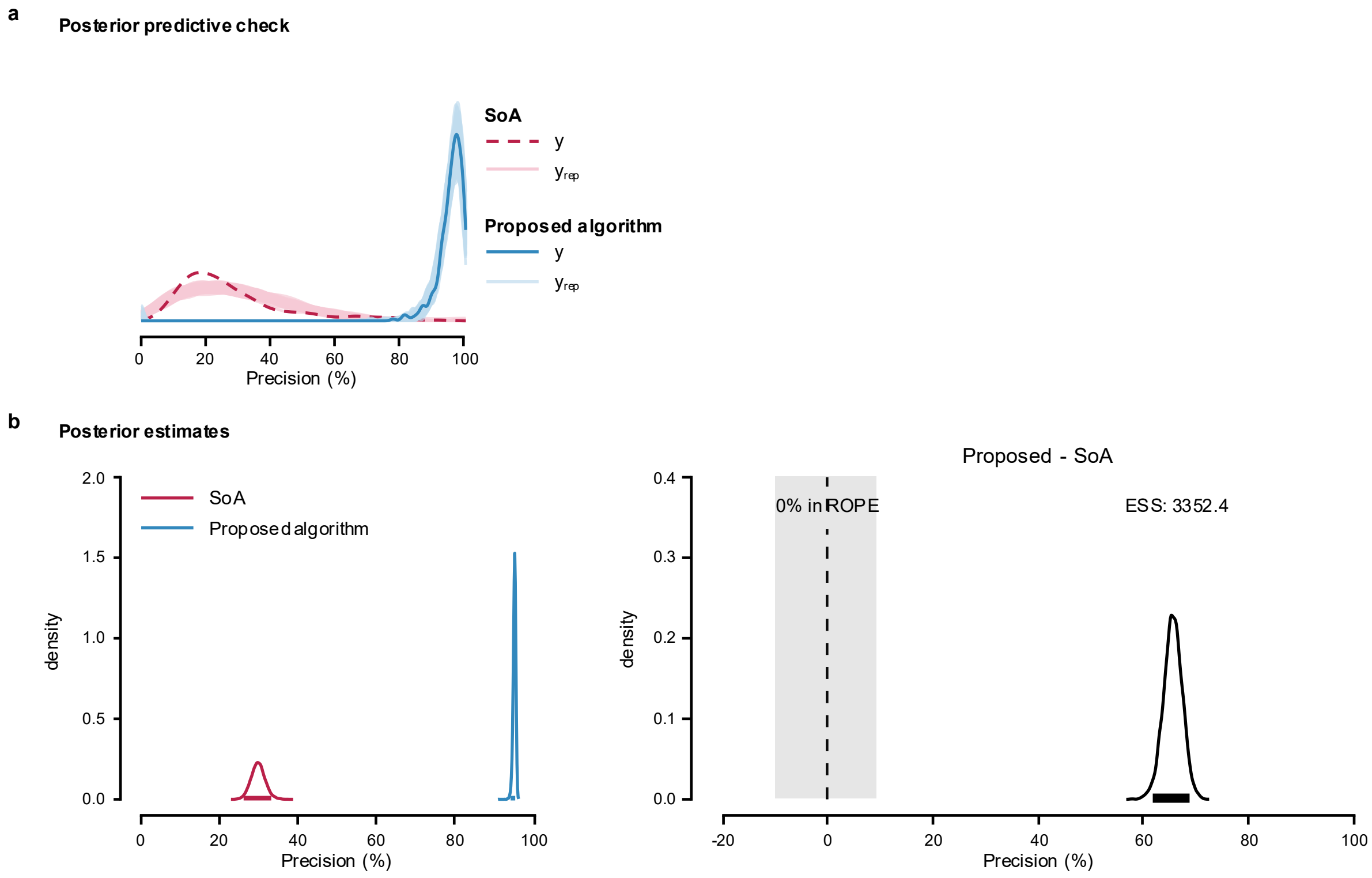

Supplementary Fig. 13: Posterior predictive check and posterior estimates for Model 2 of firing identification precision in tracked TA MUs across synthetic grid displacement. **a**, Posterior predictive check of the model; **b**, Posterior distributions and the difference between means estimated using Bayesian Model 2 to compare the performance of the proposed algorithm and SoA method.

<u>Model 3</u>

The maximum PSRF across all parameters was 1.001, indicating good convergence, while the minimum ESS was 3,553.260, indicating stable estimates of the bounds of the credible intervals (Supplementary Table 17). Posterior summaries are presented as medians with 95% ETIs and 95% HDIs (Supplementary Table 18). Group-level correlations were retained on their native scales.

Posterior predictive checks were performed by overlaying model-generated predictions on the observed data (Supplementary Fig. 14a). The posterior distributions (Supplementary Fig.

14b) indicated that the proposed algorithm achieved a higher precision (95.2%, [94.4-95.8%]) than the SoA algorithm (29.5%, [26.0-33.1%]). The posterior distribution of the difference excluded zero, and less than 2.5% of its distribution fell within ROPE.

Supplementary Table 17: Posterior MCMC diagnostics for Model 3 of firing identification precision in tracked TA MUs across synthetic grid displacement.

	PSRF Pt	PSRF UpCI	ESS
α_{μ}	1.000	1.001	6,795.168
α_{ϕ}	1.000	1.000	1,5773.480
α_{ζ}	1.000	1.000	2,2353.260
α_{κ}	1.000	1.001	3,2691.363
$\beta_{\mu,\text{Proposed}}$	1.000	1.001	8,952.588
$\beta_{\phi,\text{Proposed}}$	1.000	1.000	3,3791.316
$\beta_{\zeta,\text{Proposed}}$	1.000	1.000	2,1912.922
$\beta_{\kappa,\text{Proposed}}$	1.000	1.001	2,9337.459
τ_{b0}	1.000	1.001	7,083.752
$\tau_{b\text{Proposed}}$	1.000	1.001	9,589.969
τ_{c}	1.000	1.000	3,553.260
R_{12}	1.000	1.001	1,1441.921

Supplementary Table 18: Posterior summaries for Model 3 of firing identification precision in tracked TA MUs across synthetic grid displacement.

	50%	95% ETI		Mode	95% HDI	
		2.5%	97.5%		low	high
α_μ	0.294	0.260	0.331	0.292	0.259	0.330
α_ϕ	9.450	8.091	11.097	9.290	8.057	11.039
α_ζ	0.005	0.001	0.016	0.004	0.001	0.014
α_κ	0.212	0.009	0.881	0.049	0.000	0.817
$\beta_{\mu,\text{Proposed}}$	47.747	39.405	57.501	47.403	38.789	56.728
$\beta_{\phi,\text{Proposed}}$	4.475	3.587	5.563	4.502	3.545	5.509
$\beta_{\zeta,\text{Proposed}}$	13.829	4.306	64.855	9.154	2.304	49.753
$\beta_{\kappa,\text{Proposed}}$	110.214	2.057	8,939.829	-123.690	0.030	4,369.792
τ_{b0}	0.403	0.286	0.572	0.394	0.281	0.562
$\tau_{b\text{Proposed}}$	0.390	0.244	0.580	0.374	0.235	0.567
τ_c	0.069	0.003	0.208	0.019	0.000	0.185
R_{12}	-0.878	-0.973	-0.623	-0.913	-0.989	-0.677

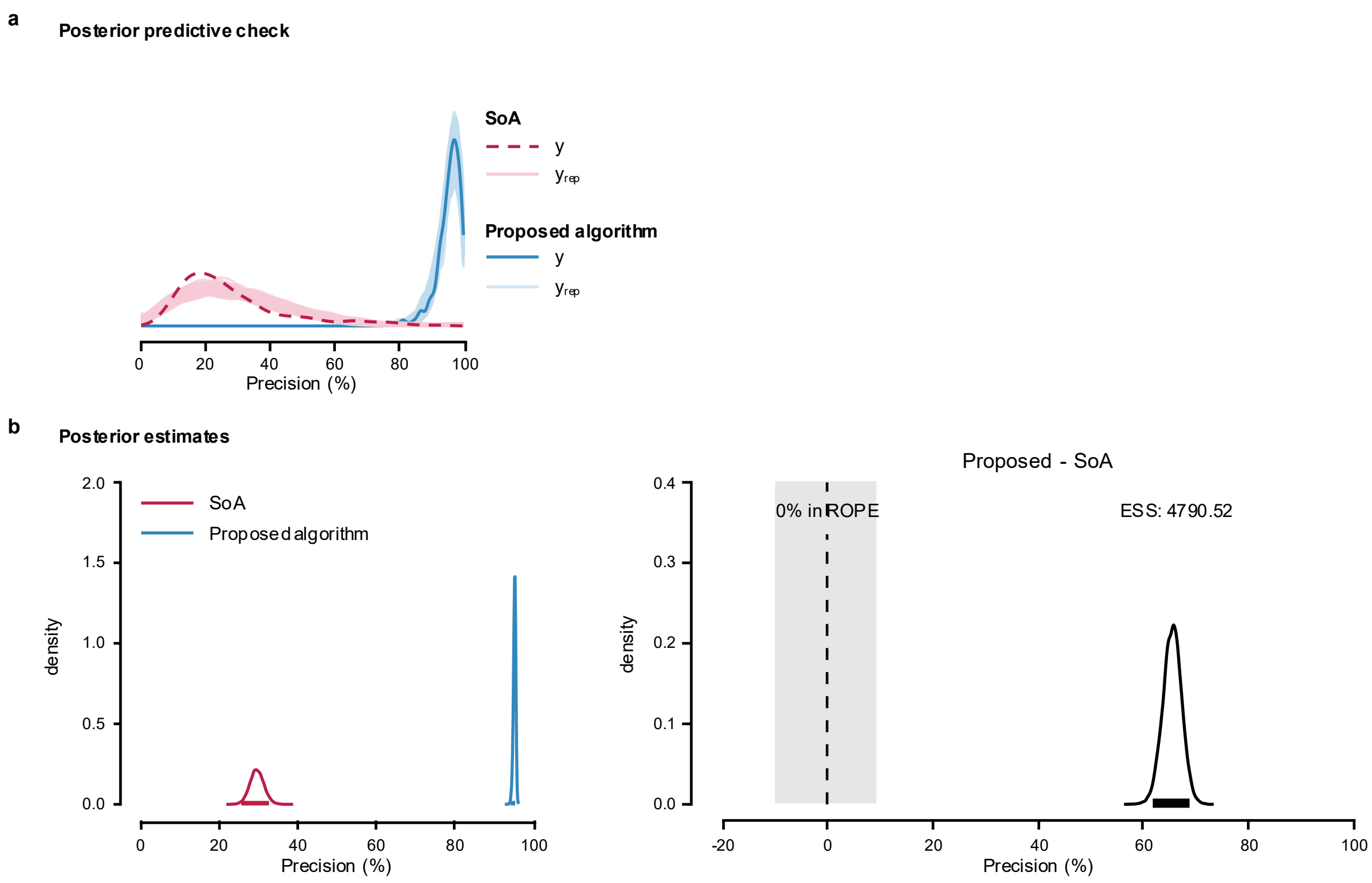

Supplementary Fig. 14: Posterior predictive check and posterior estimates for Model 3 of firing identification precision in tracked TA MUs across synthetic grid displacement. **a**, Posterior predictive check of the model; **b**, Posterior distributions and the difference between means estimated using Bayesian Model 1 to compare the performance of the proposed algorithm and SoA method.

MODEL COMPARISON

Model 2 showed the greatest expected predictive accuracy. ELPD diff relative to Model 2 were −0.7 (SE = 0.5) for Model 3 and −18.5 (SE = 5.8) for Model 1, suggesting similar predictive performance for Models 2 and 3 and reduced performance for Model 1.

Supplementary Note 9. Comparison of firing identification PNR for tracked MG MUs across synthetic grid displacement.

Model implementation, likelihood function, and priors are detailed in Supplementary Note 5. The full dataset contains firing identification PNR values for 300 tracked MUs in the medial gastrocnemius (MG) muscle (Supplementary Fig. 15). The MUs firings were obtained by applying the MU filters calculated using the SoA algorithm[5] and the proposed algorithm to the HDsEMG signals generated with synthetic grid displacement.

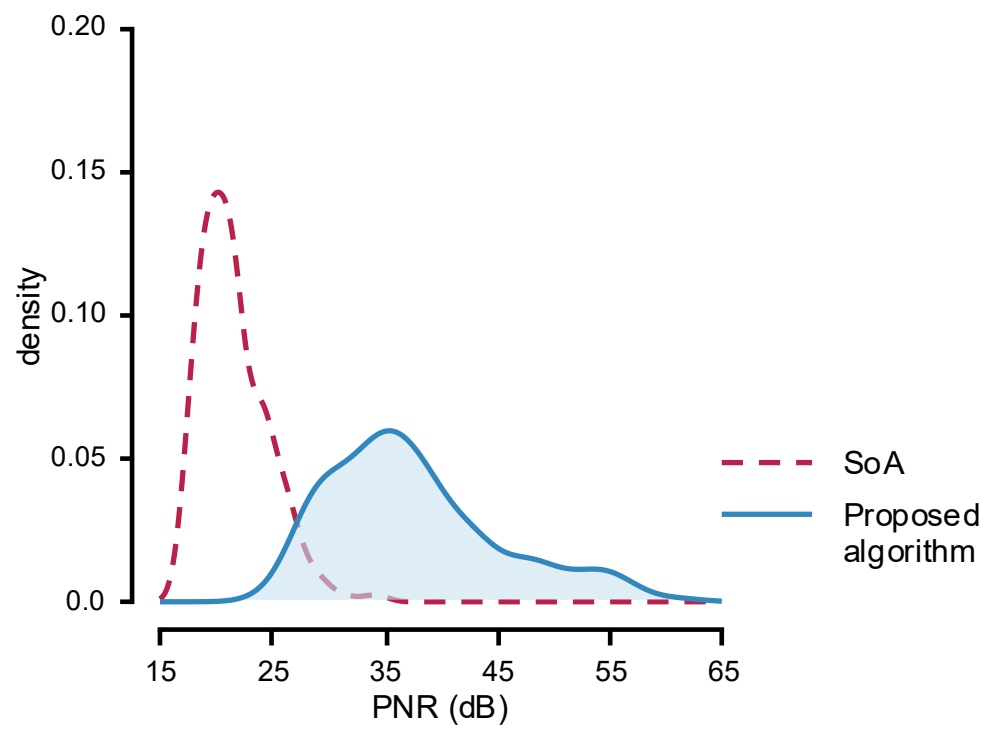

Supplementary Fig. 15: Distribution of firing identification PNR in tracked MG MUs across synthetic grid displacement.

PNR values were calculated from firing identification obtained by applying MU filters calculated using SoA (dashed line) and the proposed algorithms (solid line) to the HDsEMG signals generated with synthetic grid displacement.

In all models, the lognormal mean and sigma effects were exponentiated before the posterior summaries were calculated. Group-level standard deviations (SDs) are reported on their native model or link scales. Posterior diagnostics include the point estimate and upper confidence limit of the potential scale reduction factor (PSRF Pt and PSRF UpCI, respectively), as well as the effective sample size (ESS). Model selection was based on the difference in expected log pointwise predictive density (ELPD diff) and its standard error (SE diff).

Model 1

The maximum PSRF across all parameters was 1.001, indicating good convergence, while the minimum ESS was 6,091.227, indicating stable estimates of the bounds of the credible intervals (Supplementary Table 19). Posterior summaries are presented as medians with 95% ETI and the 95% HDI (Supplementary Table 20).

Supplementary Table 19: Posterior MCMC diagnostics for Model 1 of firing identification PNR in tracked MG MUs across synthetic grid displacement.

	PSRF Pt	PSRF UpCI	ESS
α_μ	1.000	1.000	7,750.348
α_σ	1.000	1.000	2,0,672.677
$\beta_{\mu,\text{Proposed}}$	1.000	1.000	3,2,281.335
$\beta_{\sigma,\text{Proposed}}$	1.000	1.000	2,0,251.462
τ_μ	1.000	1.001	6,091.227

Supplementary Table 20: Posterior summaries for Model 1 of firing identification PNR in tracked MG MUs across synthetic grid displacement.

	50%	95% ETI		Mode	95% HDI	
		2.5%	97.5%		low	high
α_μ	21.721	21.085	22.387	21.703	21.070	22.370
α_σ	1.718	1.673	1.765	1.718	1.673	1.764
$\beta_{\mu,\text{Proposed}}$	0.117	0.106	0.129	0.116	0.105	0.129
$\beta_{\sigma,\text{Proposed}}$	1.726	1.481	1.996	1.711	1.483	1.998
τ_μ	0.059	0.039	0.090	0.058	0.038	0.088

Posterior predictive checks were performed by overlaying model-generated predictions on the observed data (Supplementary Fig. 16a). The posterior distributions (Supplementary Fig. 16b) indicated that the proposed algorithm achieved a higher PNR (38.1 dB, [36.8-39.5 dB]) than the SoA algorithm (21.9 dB, [21.2-22.5 dB]). The posterior distribution of the difference excluded zero, and less than 2.5% of its distribution fell within ROPE.

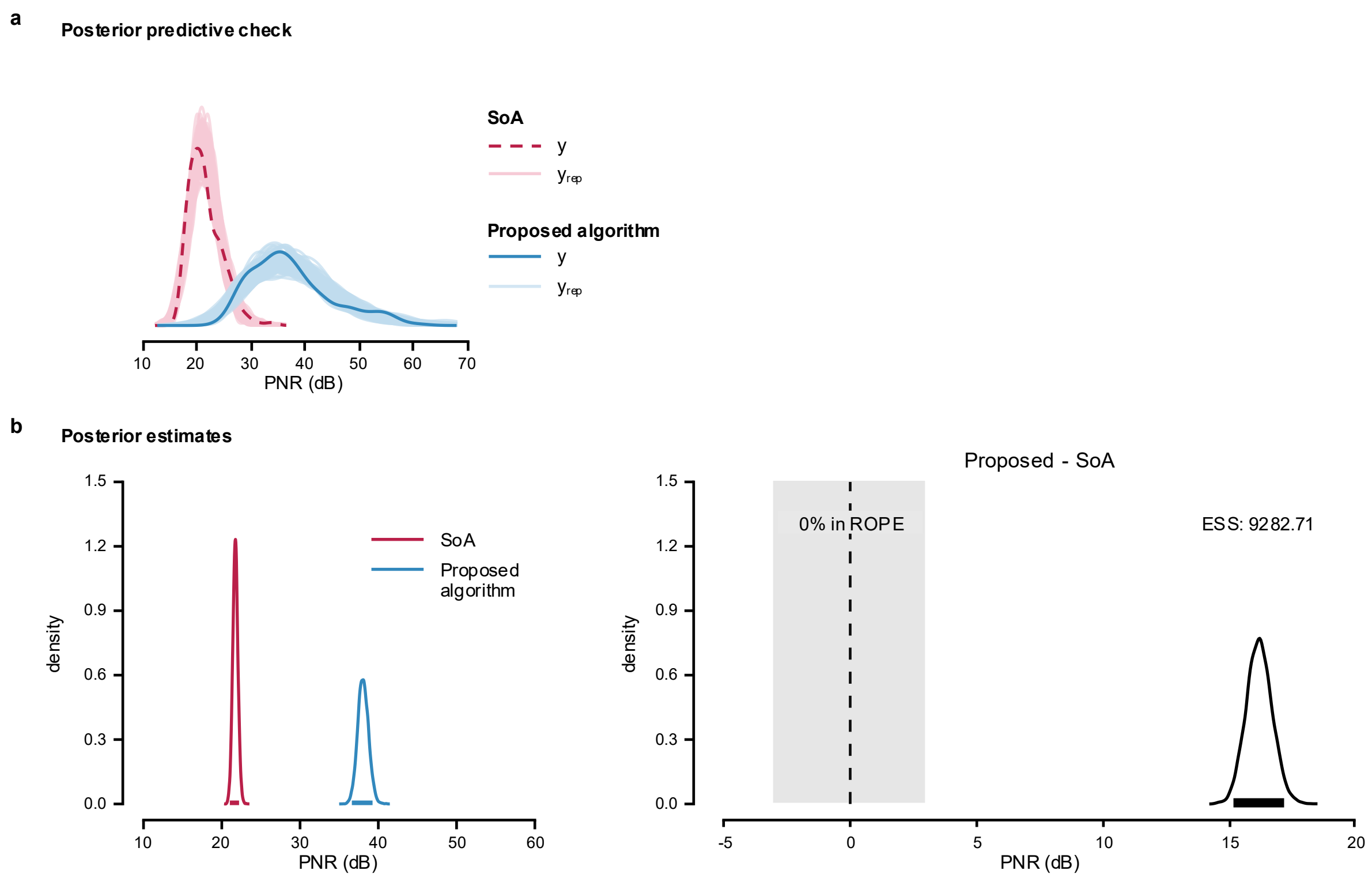

Supplementary Fig. 16: Posterior predictive check and posterior estimates for Model 1 of firing identification PNR in tracked MG MUs across synthetic grid displacement. **a**, Posterior predictive check of the model; **b**, Posterior distributions and the difference between means estimated using Bayesian Model 1 to compare the performance of the proposed algorithm and SoA method.

Model 2

The maximum PSRF across all parameters was 1.004, indicating good convergence, while the minimum ESS was 5,179.098, indicating stable estimates of the bounds of the credible intervals (Supplementary Table 21). Posterior summaries are reported as medians with 95% ETIs and HDIs. Group-level correlations were retained on their native scales (Supplementary Table 22).

Posterior predictive checks were performed by overlaying model-generated predictions on the observed data (Supplementary Fig. 17a). The posterior distributions (Supplementary Fig. 17b) indicated that the proposed algorithm achieved a higher PNR (35.9 dB, [33.6-38.2 dB]) than the SoA algorithm (22.3 dB, [21.4-23.2 dB]). The posterior distribution of the difference excluded zero, and less than 2.5% of its distribution fell within ROPE.

Model 3

The maximum PSRF across all parameters was 1.011, indicating good convergence, while the minimum ESS was 1,576.913, indicating stable estimates of the bounds of the credible intervals (Supplementary Table 23). Posterior estimates are summarized in Supplementary Table 24 by the median, 95% ETI and 95% HDI. Correlations were summarized on their native scales.

Posterior predictive checks were performed by overlaying model-generated predictions on the observed data (Supplementary Fig. 18a). The posterior distributions (Supplementary Fig. 18b) indicated that the proposed algorithm achieved a higher PNR (35.9 dB, [33.6-38.2 dB]) than the SoA algorithm (22.3 dB, [21.4-23.2 dB]). The posterior distribution of the difference excluded zero, and less than 2.5% of its distribution fell within ROPE.

Supplementary Table 21: Posterior MCMC diagnostics for Model 2 of firing identification PNR in tracked MG MUs across synthetic grid displacement.

	PSRF Pt	PSRF UpCI	ESS
α_{μ}	1.001	1.001	5,308.435
α_{σ}	1.000	1.000	2,4,854.807
$\beta_{\mu,\text{Proposed}}$	1.001	1.001	5,386.448
$\beta_{\sigma,\text{Proposed}}$	1.000	1.001	2,5,237.093
τ_{b0}	1.001	1.002	5,263.395
$\tau_{b\text{Proposed}}$	1.000	1.001	5,111.800
R_{12}	1.001	1.004	5,179.098

Supplementary Table 22: Posterior summaries for Model 2 of firing identification PNR in tracked MG MUs across synthetic grid displacement.

	50%	95% ETI		Mode	95% HDI	
		2.5%	97.5%		low	high
α_{μ}	22.163	21.303	23.098	22.149	21.278	23.062
α_{σ}	0.104	0.096	0.114	0.104	0.096	0.113
$\beta_{\mu,\text{Proposed}}$	1.603	1.470	1.734	1.600	1.468	1.731
$\beta_{\sigma,\text{Proposed}}$	1.456	1.296	1.631	1.464	1.292	1.624
τ_{b0}	0.087	0.064	0.125	0.084	0.061	0.121
$\tau_{b\text{Proposed}}$	0.184	0.136	0.260	0.178	0.131	0.253
R_{12}	-0.689	-0.872	-0.337	-0.738	-0.896	-0.395

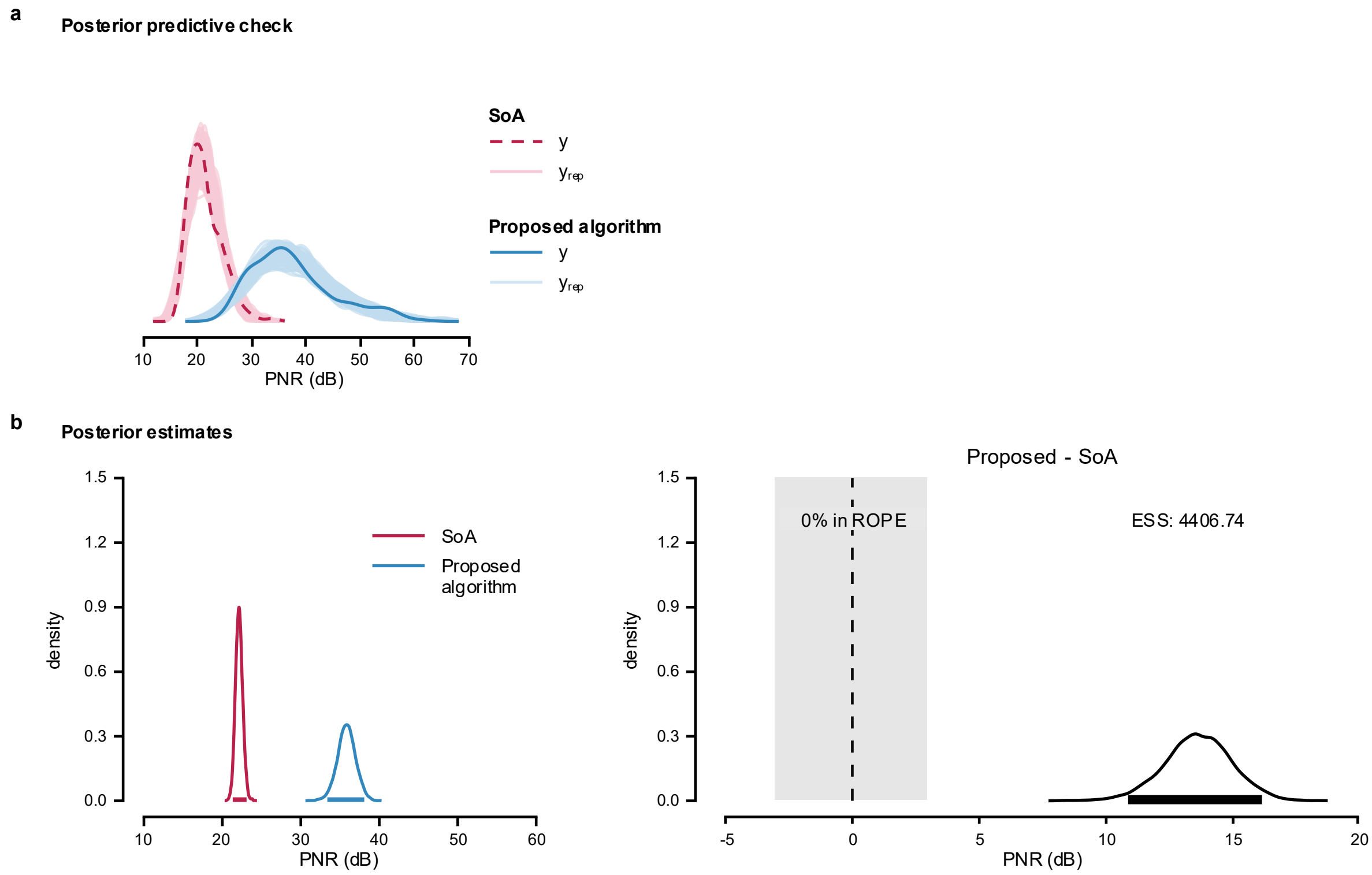

Supplementary Fig. 17: Posterior predictive check and posterior estimates for Model 2 of firing identification PNR in tracked MG muscle MUs across synthetic grid displacement.

a, Posterior predictive check of the model; **b**, Posterior distributions and the difference in means from Bayesian Model 2, used to compare the performance of the proposed algorithm and SoA method.

Supplementary Table 23: Posterior MCMC diagnostics for Model 3 of firing identification PNR in tracked MG MUs across synthetic grid displacement.

	PSRF Pt	PSRF UpCI	ESS
α_{μ}	1.001	1.002	3,967.724
α_{σ}	1.001	1.004	3,130.600
$\beta_{\mu,\text{Proposed}}$	1.000	1.001	4,121.262
$\beta_{\sigma,\text{Proposed}}$	1.001	1.001	9,478.626
τ_{b0}	1.000	1.000	4,946.821
$\tau_{b\text{Proposed}}$	1.000	1.000	4,814.260
τ_{c}	1.004	1.011	1,576.913
R_{12}	1.002	1.004	4,792.227

Supplementary Table 24: Posterior summaries for Model 3 of firing identification PNR in tracked MG MUs across synthetic grid displacement.

	50%	95% ETI		Mode	95% HDI	
		2.5%	97.5%		low	high
α_{μ}	22.160	21.307	23.111	22.169	21.318	23.121
α_{σ}	0.099	0.087	0.110	0.099	0.087	0.110
$\beta_{\mu,\text{Proposed}}$	1.603	1.471	1.733	1.608	1.476	1.736
$\beta_{\sigma,\text{Proposed}}$	1.495	1.318	1.718	1.478	1.313	1.710
τ_{b0}	0.088	0.064	0.127	0.085	0.060	0.122
$\tau_{b\text{Proposed}}$	0.184	0.136	0.265	0.175	0.131	0.255
τ_{c}	0.033	0.003	0.056	0.038	0.000	0.053
R_{12}	-0.697	-0.876	-0.340	-0.735	-0.900	-0.391

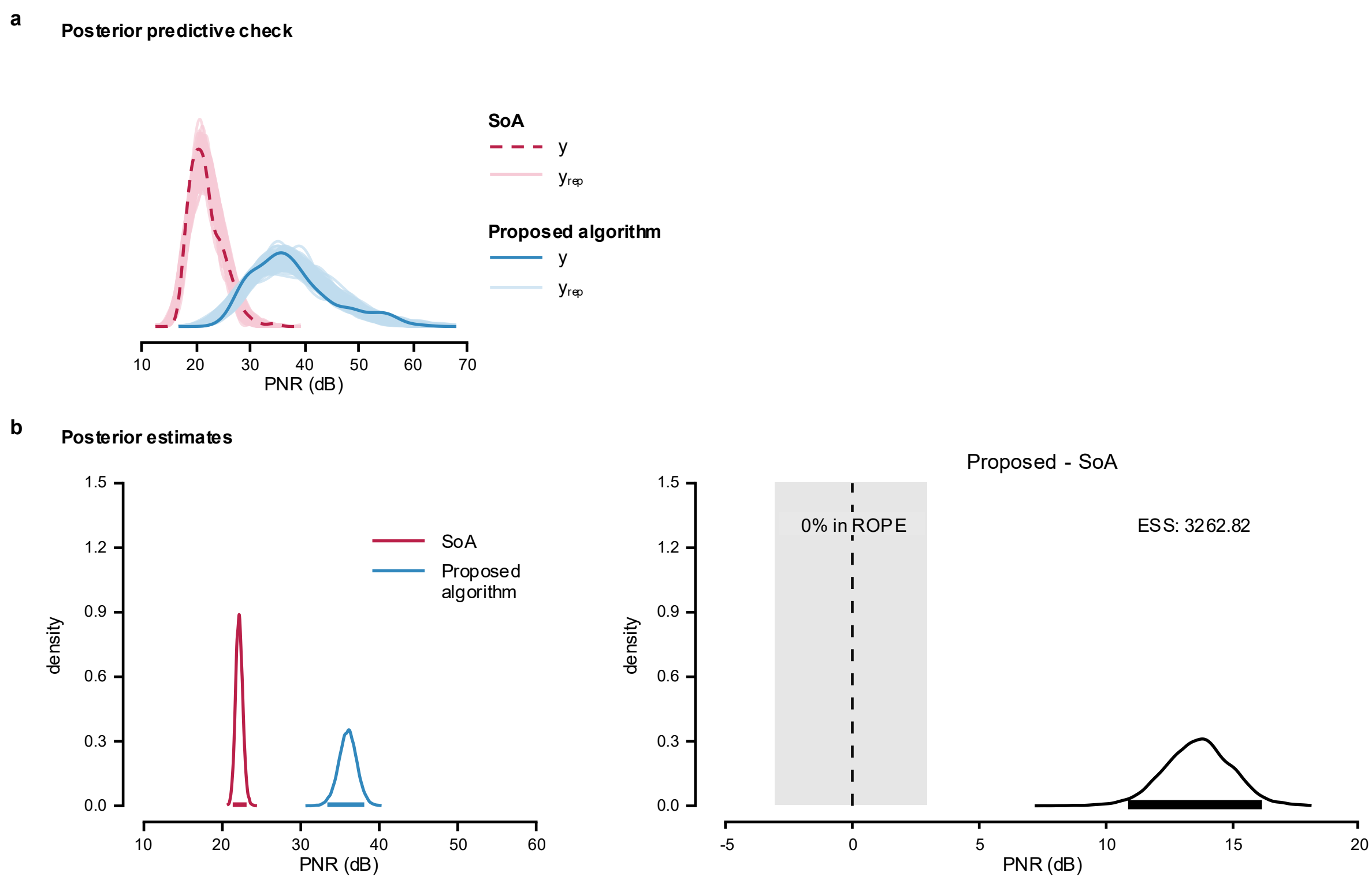

Supplementary Fig. 18: Posterior predictive check and posterior estimates for Model 3 of firing identification PNR in tracked MG MUs across synthetic grid displacement. **a**, Posterior predictive check of the model; **b**, Posterior distributions and the difference in means from Bayesian Model 3, used to compare the performance of the proposed algorithm and SoA method.

MODEL COMPARISON

Model 3 showed the greatest expected predictive accuracy. ELPD diff relative to Model 3 were −1.6 (SE = 1.8) for Model 2 and −110.6 (SE = 13.5) for Model 1, suggesting similar predictive performance for Models 2 and 3 and reduced performance for Model 1.

Supplementary Note 10. Comparison of firing identification sensitivity in tracked MG MUs across synthetic grid displacement.

Model implementation, likelihood function, and priors are detailed in Supplementary Note 5. The full dataset contains firing identification sensitivity values for 300 tracked MG MUs (hereafter referred to as 'sensitivity'). The MUs firings were obtained by applying the MU filters calculated using the SoA algorithm[5] and the proposed algorithm to the HDsEMG signals generated with synthetic grid displacement. Distribution of the data is shown in Supplementary Fig. 19.

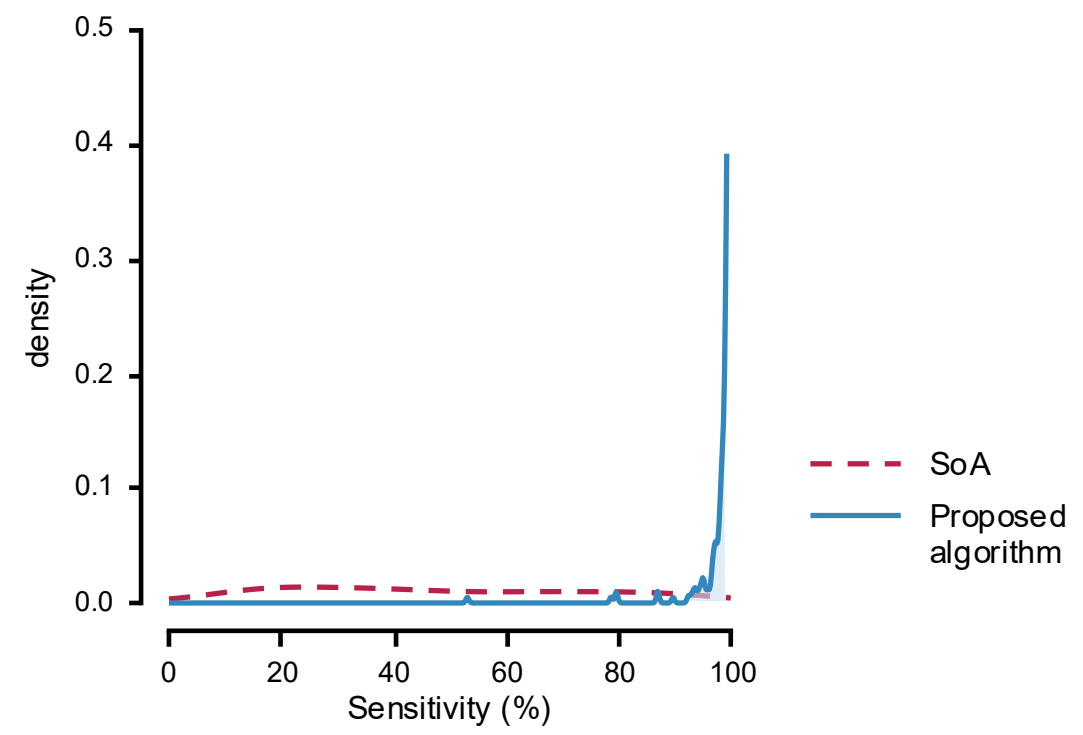

Supplementary Fig. 19: Distribution of firing identification sensitivity in tracked MG MUs across synthetic grid displacement.

Sensitivity values were calculated from firing identification obtained by applying MU filters calculated using SoA (dashed line) and the proposed algorithms (solid line) to the HDsEMG signals generated with synthetic grid displacement.

In all models, ZOIB parameters were back transformed into their interpretable component scales: *mu*, *zoi*, and *coi* to probabilities, and *phi* to the positive precision scale. Group-level SDs are reported on their native model/link scale.

Model 1

The maximum PSRF across all parameters was 1.004, indicating good convergence, while the minimum ESS was 5,659.884, indicating stable estimates of the bounds of the credible intervals (Supplementary Table 25). Posterior summaries are reported in Supplementary Table 26 as medians with 95% ETIs and 95% HDIs.

Posterior predictive checks were performed by overlaying model-generated predictions on the observed data (Supplementary Fig. 20a). The posterior distributions (Supplementary Fig. 20b) indicated that the proposed algorithm achieved a higher sensitivity (98.3%, [97.3-98.9 %]) than the SoA algorithm (51.0%, [46.0-56.3%]). The posterior distribution of the difference excluded zero, and less than 2.5% of its distribution fell within ROPE.

Supplementary Table 25: Posterior MCMC diagnostics for Model 1 of firing identification sensitivity in tracked MG MUs across synthetic grid displacement.

	PSRF Pt	PSRF UpCI	ESS
α_{μ}	1.001	1.003	5,659.884
α_{ϕ}	1.000	1.000	2,7610.853
α_{ζ}	1.000	1.001	1,0711.309
α_{κ}	1.000	1.001	1,4355.252
$\beta_{\mu,\text{Proposed}}$	1.000	1.000	1,4347.463
$\beta_{\phi,\text{Proposed}}$	1.000	1.000	1,4874.786
$\beta_{\zeta,\text{Proposed}}$	1.000	1.001	1,1259.577
$\beta_{\kappa,\text{Proposed}}$	1.000	1.001	1,5329.729
τ_b	1.001	1.004	6,270.831

Supplementary Table 26: Posterior summaries for Model 1 of firing identification sensitivity in tracked MG MUs across synthetic grid displacement.

	50%	95% ETI		Mode	95% HDI	
		2.5%	97.5%		low	high
α_{μ}	0.512	0.461	0.564	0.511	0.461	0.564
α_{ϕ}	3.672	3.110	4.309	3.641	3.091	4.286
α_{ζ}	0.005	0.001	0.018	0.004	0.000	0.015
α_{κ}	0.131	0.005	0.818	0.035	0.000	0.721
$\beta_{\mu,\text{Proposed}}$	41.295	33.249	50.844	41.114	32.674	50.109
$\beta_{\phi,\text{Proposed}}$	7.995	5.802	10.869	7.864	5.691	10.672
$\beta_{\zeta,\text{Proposed}}$	167.521	49.887	872.358	101.611	29.368	654.505
$\beta_{\kappa,\text{Proposed}}$	804.378	17.382	4,7843.100	-828.998	0.506	2,4758.800
τ_{b}	0.391	0.251	0.604	0.374	0.237	0.580

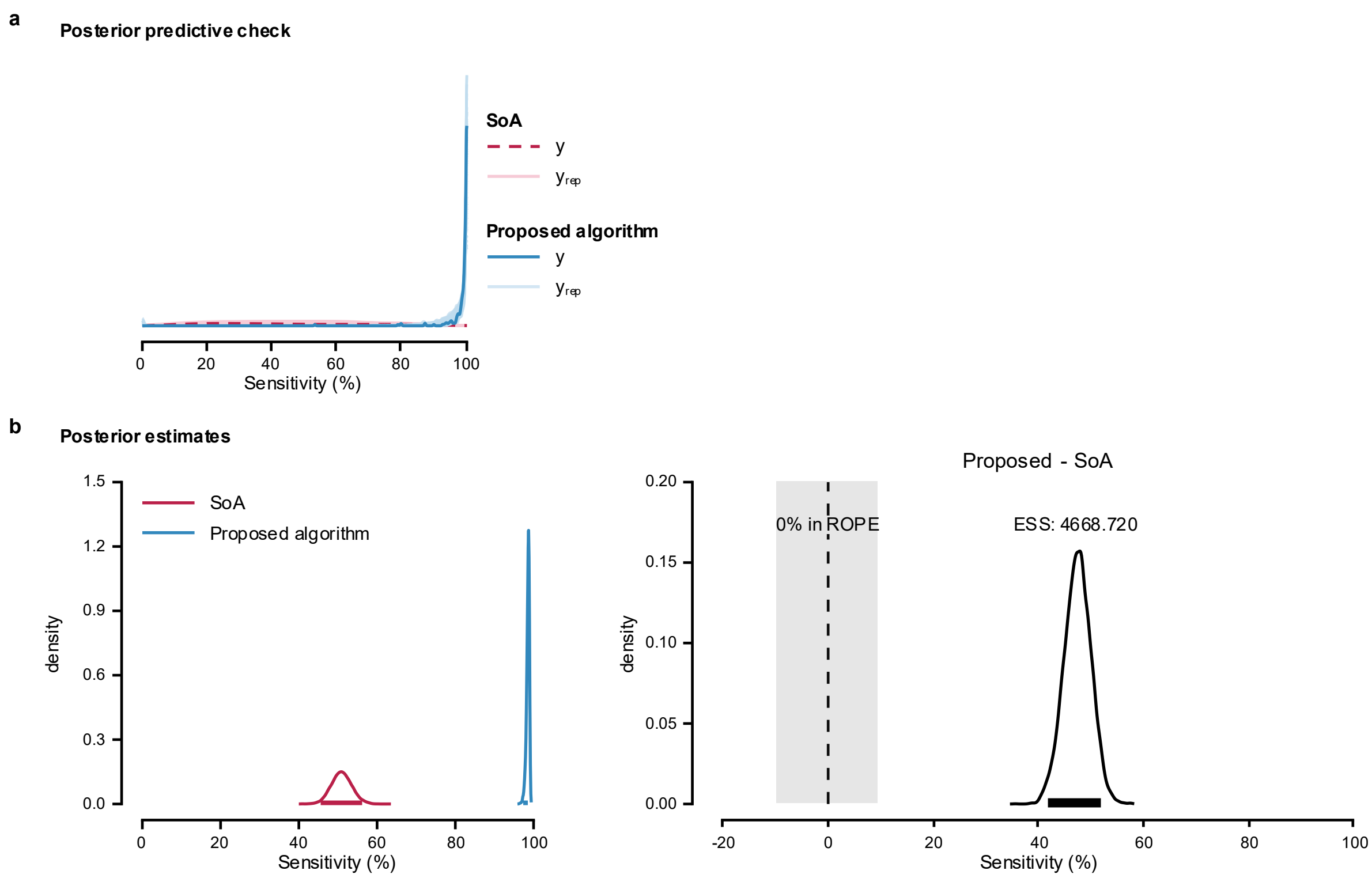

Supplementary Fig. 20: Posterior predictive check and posterior estimates for Model 1 of firing identification sensitivity in tracked MG MUs across synthetic grid displacement.

a, Posterior predictive check of the model; **b**, Posterior distributions and the difference between means estimated using Bayesian Model 1 to compare the performance of the proposed algorithm and SoA method.

Model 2

The maximum PSRF across all parameters was 1.002, indicating good convergence, while the minimum ESS was 5,153.011, indicating stable estimates of the bounds of the credible intervals (Supplementary Table 27). The medians with 95% ETIs and 95% HDIs of the posterior estimates are presented in Supplementary Table 28. Group-level correlations were summarized without transformation.

Posterior predictive checks were performed by overlaying model-generated predictions on the observed data (Supplementary Fig. 21a). The posterior distributions (Supplementary Fig. 21b) indicated that the proposed algorithm achieved a higher sensitivity (98.2%, [97.2-98.8%]) than the SoA algorithm (53.4%, [46.7-60.1%]). The posterior distribution of the difference excluded zero, and less than 2.5% of its distribution fell within ROPE.

Supplementary Table 27: Posterior MCMC diagnostics for Model 2 of firing identification sensitivity in tracked MG MUs across synthetic grid displacement.

	PSRF Pt	PSRF UpCI	ESS
α_{μ}	1.001	1.002	4,552.855
α_{ϕ}	1.000	1.001	2,9216.538
α_{ζ}	1.000	1.001	1,0302.998
α_{κ}	1.000	1.001	1,4547.824
$\beta_{\mu,\text{Proposed}}$	1.000	1.001	6,159.842
$\beta_{\phi,\text{Proposed}}$	1.000	1.000	1,4114.821
$\beta_{\zeta,\text{Proposed}}$	1.000	1.000	1,0420.945
$\beta_{\kappa,\text{Proposed}}$	1.000	1.001	1,5274.517
τ_{b0}	1.001	1.002	5,141.714
$\tau_{b\text{Proposed}}$	1.001	1.002	6,820.759
R_{12}	1.000	1.001	1,0623.000

Supplementary Table 28: Posterior summaries for Model 2 of firing identification sensitivity in tracked MG MUs across synthetic grid displacement.

	50%	95% ETI		Mode	95% HDI	
		2.5%	97.5%		low	high
α_{μ}	0.536	0.468	0.603	0.534	0.470	0.604
α_{ϕ}	4.260	3.619	4.961	4.240	3.615	4.956
α_{ζ}	0.005	0.001	0.017	0.004	0.000	0.015
α_{κ}	0.131	0.005	0.809	0.034	0.000	0.709
$\beta_{\mu,\text{Proposed}}$	33.914	24.279	47.343	33.062	23.438	46.113
$\beta_{\phi,\text{Proposed}}$	7.284	5.364	9.779	7.110	5.158	9.543
$\beta_{\zeta,\text{Proposed}}$	165.861	50.989	841.294	119.567	25.673	637.582
$\beta_{\kappa,\text{Proposed}}$	813.029	17.770	4,6822.670	-843.483	0.470	2,4428.118
τ_{b0}	0.574	0.413	0.822	0.569	0.394	0.793
$\tau_{b\text{Proposed}}$	0.585	0.380	0.885	0.572	0.359	0.855
R_{12}	-0.931	-0.991	-0.708	-0.962	-0.999	-0.761

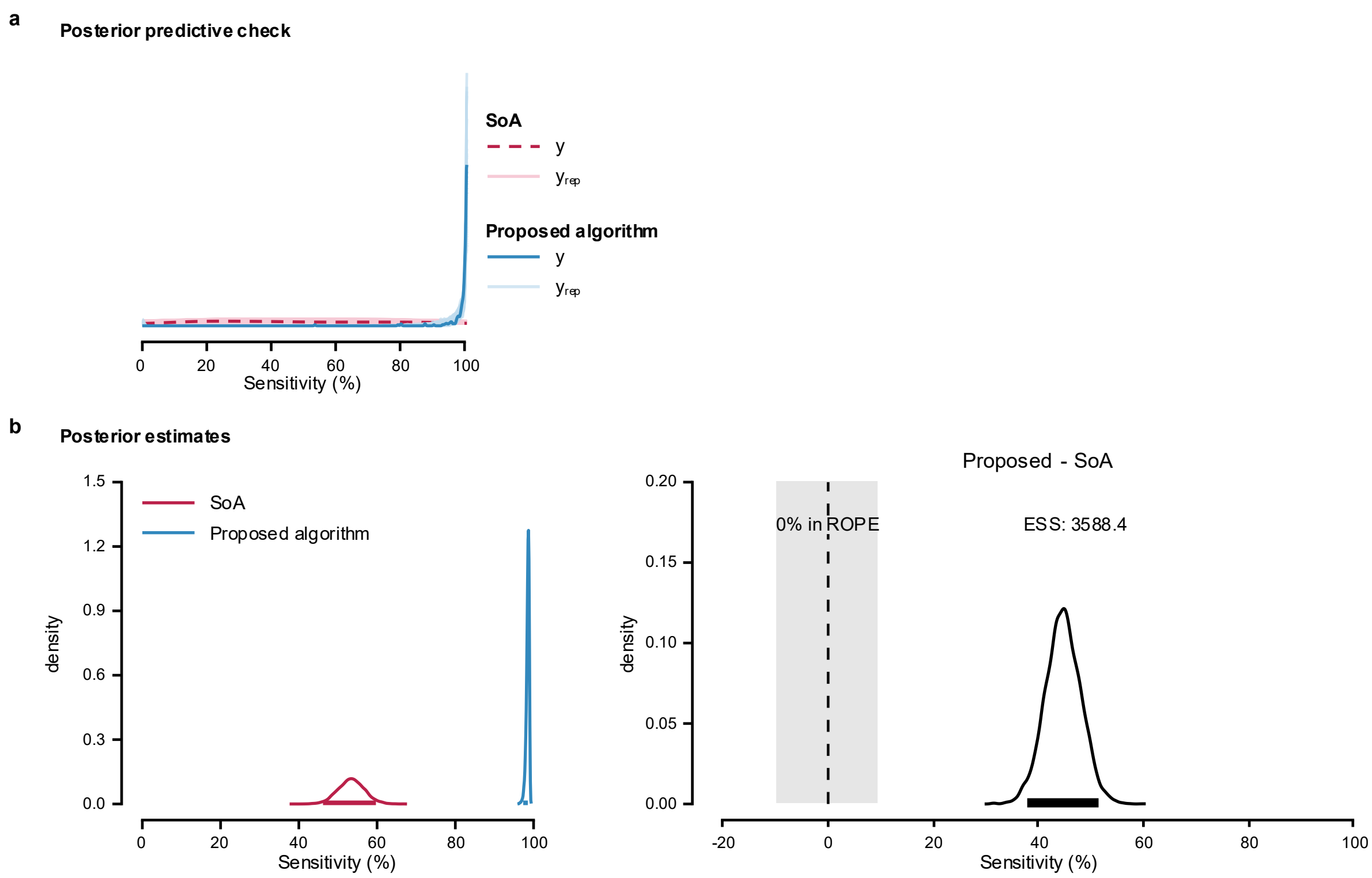

Supplementary Fig. 21: Posterior predictive check and posterior estimates for Model 2 of firing identification sensitivity in tracked MG MUs across synthetic grid displacement.

a, Posterior predictive check of the model; **b**, Posterior distributions and the difference in means from Bayesian Model 2, used to compare the performance of the proposed algorithm and SoA method.

<u>Model 3</u>

The maximum PSRF across all parameters was 1.002, indicating good convergence, while the minimum ESS was 6,270.581, indicating stable estimates of the bounds of the credible intervals (Supplementary Table 29). Posterior medians, 95% ETIs and 95% HDIs are reported on interpretable scales (Supplementary Table 30). Correlations are reported on their native model/link and correlation scales.

Posterior predictive checks were performed by overlaying model-generated predictions on the observed data (Supplementary Fig. 22a). The posterior distributions (Supplementary Fig. 22b) indicated that the proposed algorithm achieved a higher sensitivity (98.2%, [97.2-98.8%]) than the SoA algorithm (53.3%, [46.7-60.2%]). The posterior distribution of the difference excluded zero, and less than 2.5% of its distribution fell within ROPE.

Supplementary Table 29: Posterior MCMC diagnostics for Model 3 of firing identification sensitivity in tracked MG MUs across synthetic grid displacement.

	**PSRF Pt**	**PSRF UpCI**	**ESS**
α_{μ}	1.001	1.002	6,270.581
α_{ϕ}	1.000	1.000	2,3045.385
α_{ζ}	1.000	1.000	1,8057.101
α_{κ}	1.000	1.001	2,5932.481
$\beta_{\mu,\text{Proposed}}$	1.000	1.001	8,603.863
$\beta_{\phi,\text{Proposed}}$	1.000	1.000	2,3671.972
$\beta_{\zeta,\text{Proposed}}$	1.000	1.000	1,8006.556
$\beta_{\kappa,\text{Proposed}}$	1.000	1.001	2,1635.468
τ_{b0}	1.000	1.001	6,798.793
$\tau_{b\text{Proposed}}$	1.000	1.000	8,910.656
τ_{c}	1.000	1.000	6,711.978
R_{12}	1.000	1.000	1,2045.112

Supplementary Table 30: Posterior summaries for Model 3 of firing identification sensitivity in tracked MG MUs across synthetic grid displacement.

	50%	95% ETI		Mode	95% HDI	
		2.5%	97.5%		low	high
α_{μ}	0.536	0.469	0.603	0.539	0.470	0.603
α_{ϕ}	4.310	3.671	5.064	4.234	3.645	5.022
α_{ζ}	0.005	0.001	0.017	0.004	0.001	0.015
α_{κ}	0.138	0.006	0.811	0.035	0.000	0.715
$\beta_{\mu,\text{Proposed}}$	34.080	24.216	47.391	33.097	23.828	46.774
$\beta_{\phi,\text{Proposed}}$	7.259	5.311	9.717	7.078	5.252	9.638
$\beta_{\zeta,\text{Proposed}}$	164.977	51.514	832.822	113.996	26.886	635.875
$\beta_{\kappa,\text{Proposed}}$	764.369	16.831	4,7034.930	-796.872	0.475	2,3437.830
τ_{b0}	0.577	0.408	0.824	0.557	0.389	0.795
$\tau_{b\text{Proposed}}$	0.592	0.383	0.888	0.558	0.366	0.862
τ_{c}	0.064	0.003	0.224	0.017	0.000	0.193
R_{12}	-0.929	-0.990	-0.710	-0.964	-0.998	-0.763

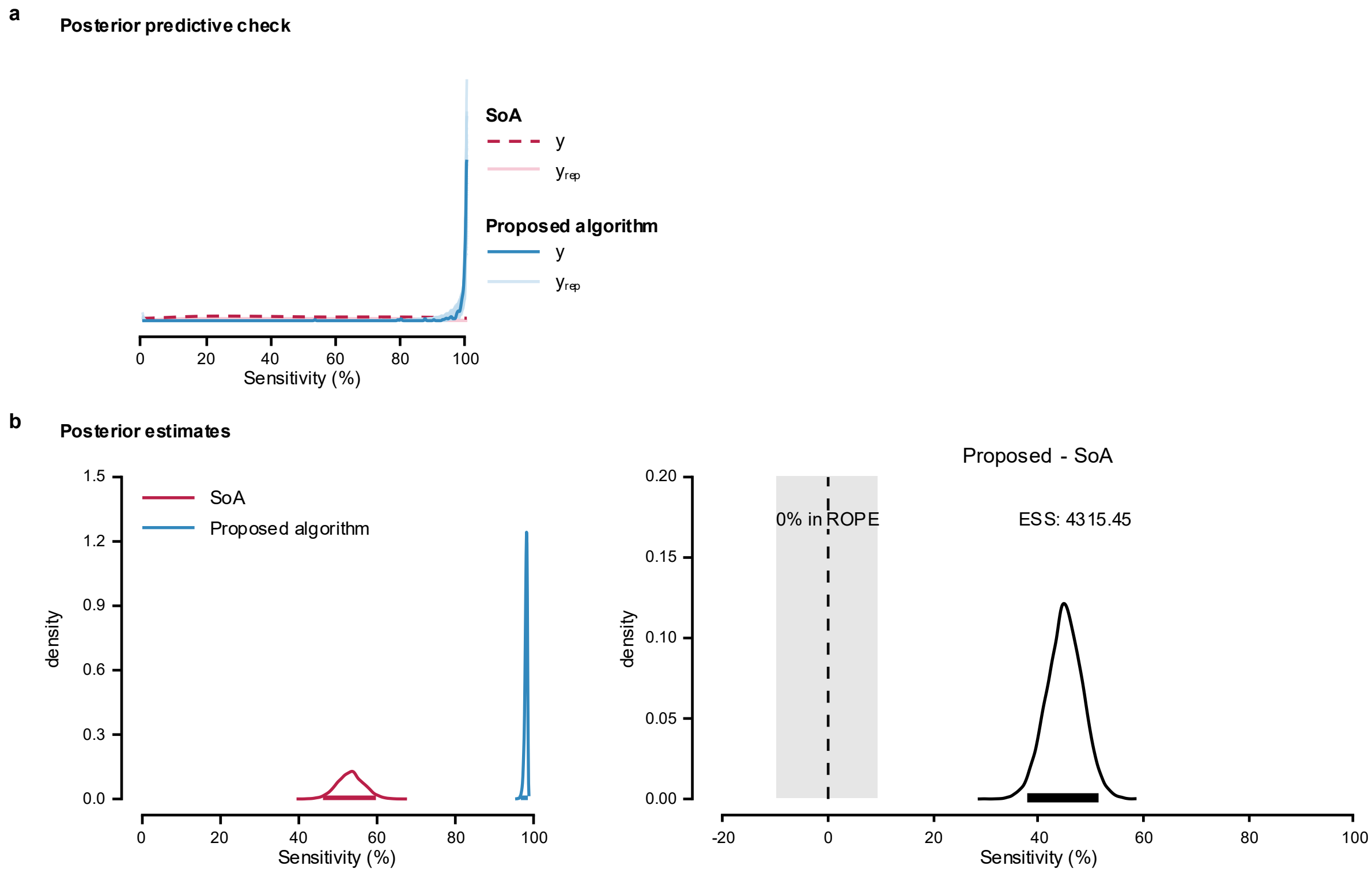

Supplementary Fig. 22: Posterior predictive check and posterior estimates for Model 3 of firing identification sensitivity in tracked MG MUs across synthetic grid displacement.

a, Posterior predictive check of the model; **b**, Posterior distributions and the difference in means from Bayesian Model 3, used to compare the performance of the proposed algorithm and SoA method.

MODEL COMPARISON

Model 2 showed the greatest expected predictive accuracy. ELPD diff relative to Model 2 were −0.8 (SE = 0.2) for Model 3 and −24.9 (SE = 5.4) for Model 1, suggesting similar predictive performance for Models 2 and 3 and reduced performance for Model 1.

Supplementary Note 11. Comparison of firing identification precision in tracked MG MUs across synthetic grid displacement.

Model implementation, likelihood function, and priors are detailed in Supplementary Note 5. The full dataset contains firing identification precision values for 300 tracked MG MUs (hereafter referred to as 'precision'). The MUs firings were obtained by applying the MU filters calculated using the SoA algorithm[5] and the proposed algorithm to the HDsEMG signals generated with synthetic grid displacement. Distribution of the data is shown in Supplementary Fig. 23.

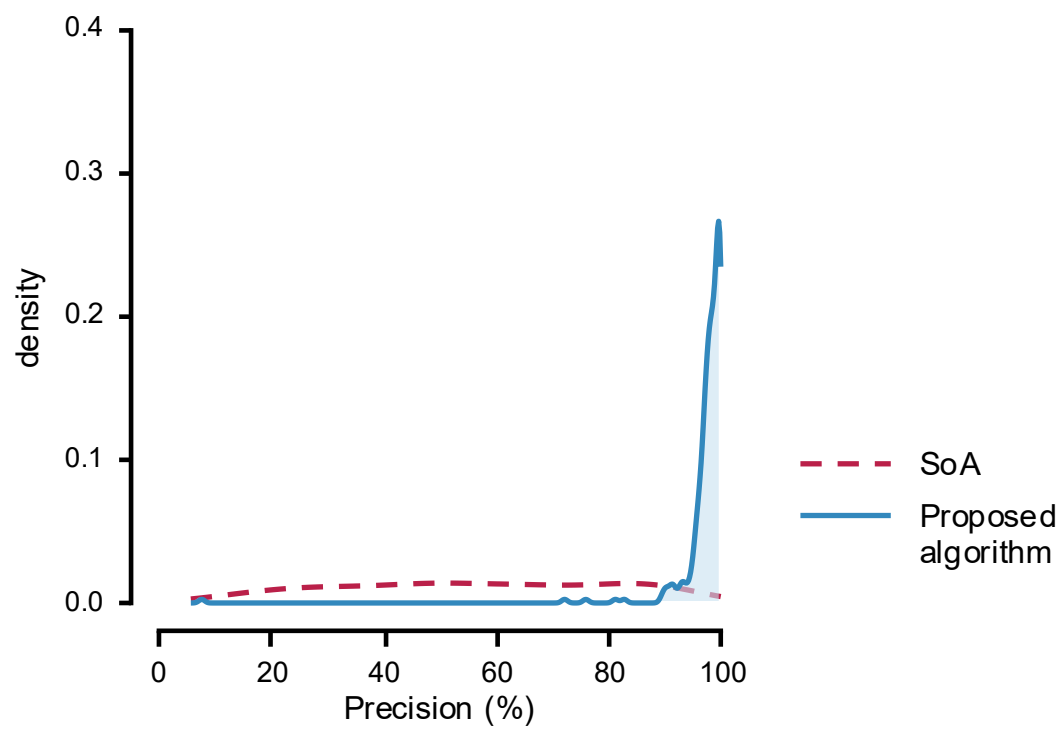

Supplementary Fig. 23: Distribution of firing identification precision in tracked MG MUs across synthetic grid displacement.

Precision values were calculated from firing identification obtained by applying MU filters calculated using SoA (dashed line) and the proposed algorithms (solid line) to the HDsEMG signals generated with synthetic grid displacement.

In all models, ZOIB parameters were back transformed into their interpretable component scales: *mu*, *zoi*, and *coi* to probabilities, and *phi* to the positive precision scale. Group-level SDs are reported on their native model/link scale.

Model 1

The maximum PSRF across all parameters was 1.002, indicating good convergence, while the minimum ESS was 7,975.097, indicating stable estimates of the bounds of the credible intervals (Supplementary Table 31). Posterior summaries are reported in Supplementary Table 32 as medians with 95% ETIs and 95% HDIs.

Posterior predictive checks were performed by overlaying model-generated predictions on the observed data (Supplementary Fig. 24a). The posterior distributions (Supplementary Fig. 24b) indicated that the proposed algorithm achieved a higher precision (96.7%, [95.7-97.4 %]) than the SoA algorithm (57.0%, [53.3-60.7%]). The posterior distribution of the difference excluded zero, and less than 2.5% of its distribution fell within ROPE.

Supplementary Table 31: Posterior MCMC diagnostics for Model 1 of firing identification precision in tracked MG MUs across synthetic grid displacement.

	PSRF Pt	PSRF UpCI	ESS
α_{μ}	1.000	1.001	1,4238.646
α_{ϕ}	1.000	1.000	2,5652.337
α_{ζ}	1.001	1.002	1,7246.424
α_{κ}	1.000	1.001	2,6810.992
$\beta_{\mu,\text{Proposed}}$	1.000	1.000	2,6089.046
$\beta_{\phi,\text{Proposed}}$	1.000	1.001	2,3659.052
$\beta_{\zeta,\text{Proposed}}$	1.001	1.002	1,7319.186
$\beta_{\kappa,\text{Proposed}}$	1.000	1.001	2,4694.811
τ_b	1.000	1.000	7,975.097

Supplementary Table 32: Posterior summaries for Model 1 of firing identification precision in tracked MG MUs across synthetic grid displacement.

	50%	95% ETI		Mode	95% HDI	
		2.5%	97.5%		low	high
α_{μ}	0.573	0.536	0.610	0.572	0.536	0.610
α_{ϕ}	3.766	3.197	4.405	3.753	3.174	4.379
α_{ζ}	0.006	0.001	0.018	0.004	0.001	0.016
α_{κ}	0.168	0.007	0.854	0.043	0.000	0.774
$\beta_{\mu,\text{Proposed}}$	19.984	16.761	23.780	19.711	16.601	23.562
$\beta_{\phi,\text{Proposed}}$	5.637	4.295	7.325	5.431	4.232	7.243
$\beta_{\zeta,\text{Proposed}}$	40.759	12.805	204.304	26.014	7.296	152.293
$\beta_{\kappa,\text{Proposed}}$	288.282	5.695	2,0479.150	-313.086	0.081	9,662.875
τ_{b}	0.243	0.127	0.397	0.243	0.115	0.383

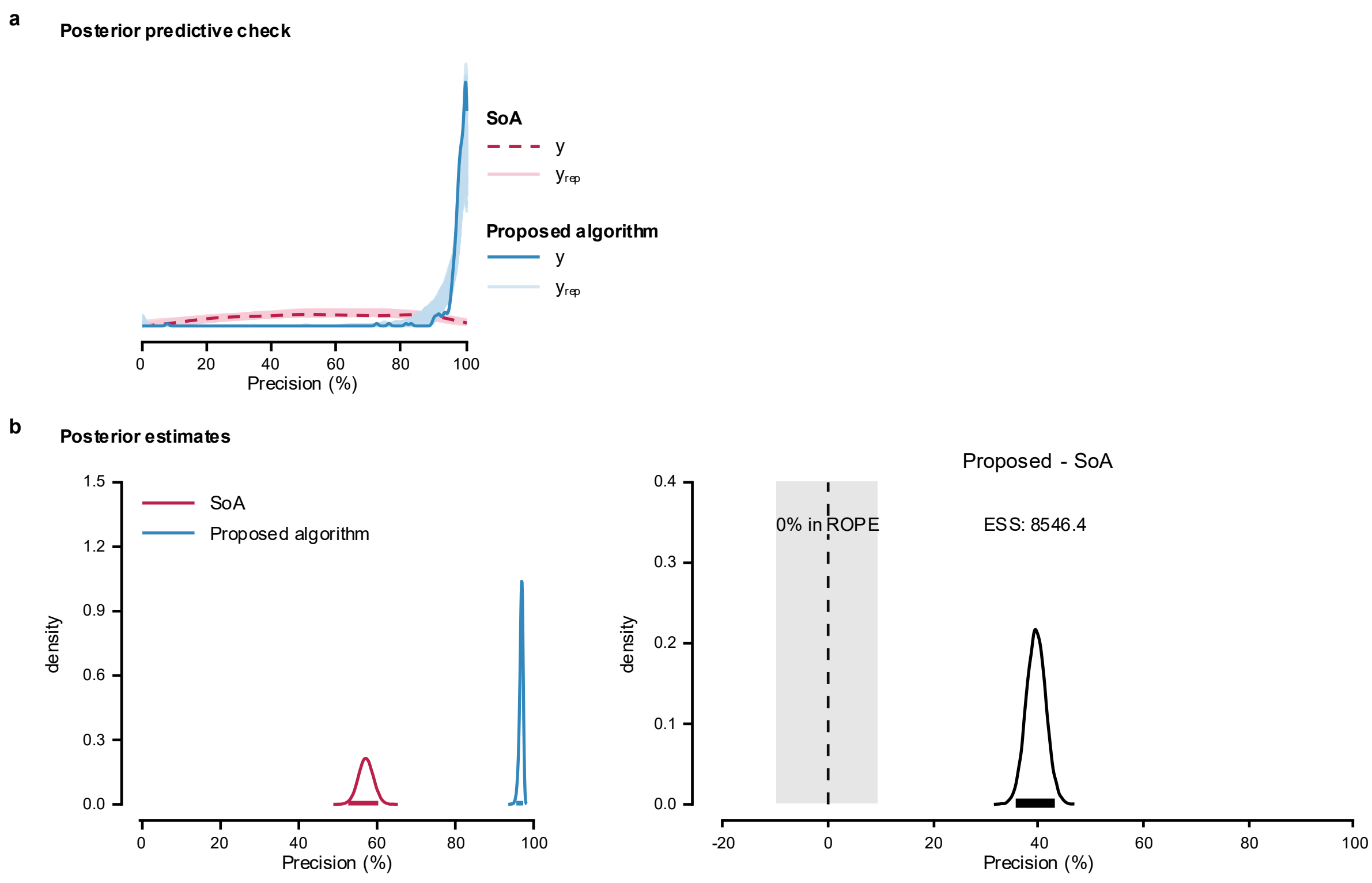

Supplementary Fig. 24: Posterior predictive check and posterior estimates for Model 1 of firing identification precision in tracked MG MUs across synthetic grid displacement. **a**, Posterior predictive check of the model; **b**, Posterior distributions and the difference between means estimated using Bayesian Model 1 to compare the performance of the proposed algorithm and SoA method.

<ins>Model 2</ins>

The maximum PSRF across all parameters was 1.005, indicating good convergence, while the minimum ESS was 4,697.237, indicating stable estimates of the bounds of the credible intervals (Supplementary Table 33). Posterior summaries as medians with 95% ETIs and 95% HDIs are reported in Supplementary Table 34. Group-level correlations are reported on the correlation scale.

Posterior predictive checks were performed by overlaying model-generated predictions on the observed data (Supplementary Fig. 25a). The posterior distributions (Supplementary Fig.

25b) indicated that the proposed algorithm achieved a higher precision (96.5%, [95.5-97.2%]) than the SoA algorithm (60.0%, [54.6-65.6%]). The posterior distribution of the difference excluded zero, and less than 2.5% of its distribution fell within ROPE.

Supplementary Table 33: Posterior MCMC diagnostics for Model 2 of firing identification precision in tracked MG MUs across synthetic grid displacement.

	**PSRF Pt**	**PSRF UpCI**	**ESS**
α_{μ}	1.000	1.001	4,697.237
α_{ϕ}	1.000	1.001	2,6948.408
α_{ζ}	1.000	1.000	1,0601.011
α_{κ}	1.000	1.001	1,4062.031
$\beta_{\mu,\text{Proposed}}$	1.000	1.001	5,975.843
$\beta_{\phi,\text{Proposed}}$	1.000	1.001	1,6215.100
$\beta_{\zeta,\text{Proposed}}$	1.000	1.000	1,0766.322
$\beta_{\kappa,\text{Proposed}}$	1.000	1.001	1,5707.576
τ_{b0}	1.002	1.005	5,390.199
$\tau_{b\text{Proposed}}$	1.001	1.003	6,270.154
R_{12}	1.000	1.000	7,627.012

Supplementary Table 34: Posterior summaries for Model 2 of firing identification precision in tracked MG MUs across synthetic grid displacement.

	50%	95% ETI		Mode	95% HDI	
		2.5%	97.5%		low	high
α_{μ}	0.602	0.549	0.658	0.603	0.550	0.658
α_{ϕ}	4.527	3.873	5.269	4.519	3.859	5.249
α_{ζ}	0.006	0.001	0.018	0.004	0.001	0.016
α_{κ}	0.168	0.007	0.845	0.044	0.000	0.770
$\beta_{\mu,\text{Proposed}}$	16.691	12.324	22.223	16.650	12.085	21.884
$\beta_{\phi,\text{Proposed}}$	4.992	3.844	6.421	4.991	3.765	6.313
$\beta_{\zeta,\text{Proposed}}$	41.291	12.603	200.589	27.793	7.144	149.862
$\beta_{\kappa,\text{Proposed}}$	291.746	5.888	2,1124.880	-312.421	0.143	1,0045.306
τ_{b0}	0.472	0.329	0.685	0.447	0.321	0.670
$\tau_{b\text{Proposed}}$	0.547	0.364	0.809	0.540	0.359	0.799
R_{12}	-0.896	-0.983	-0.644	-0.936	-0.994	-0.698

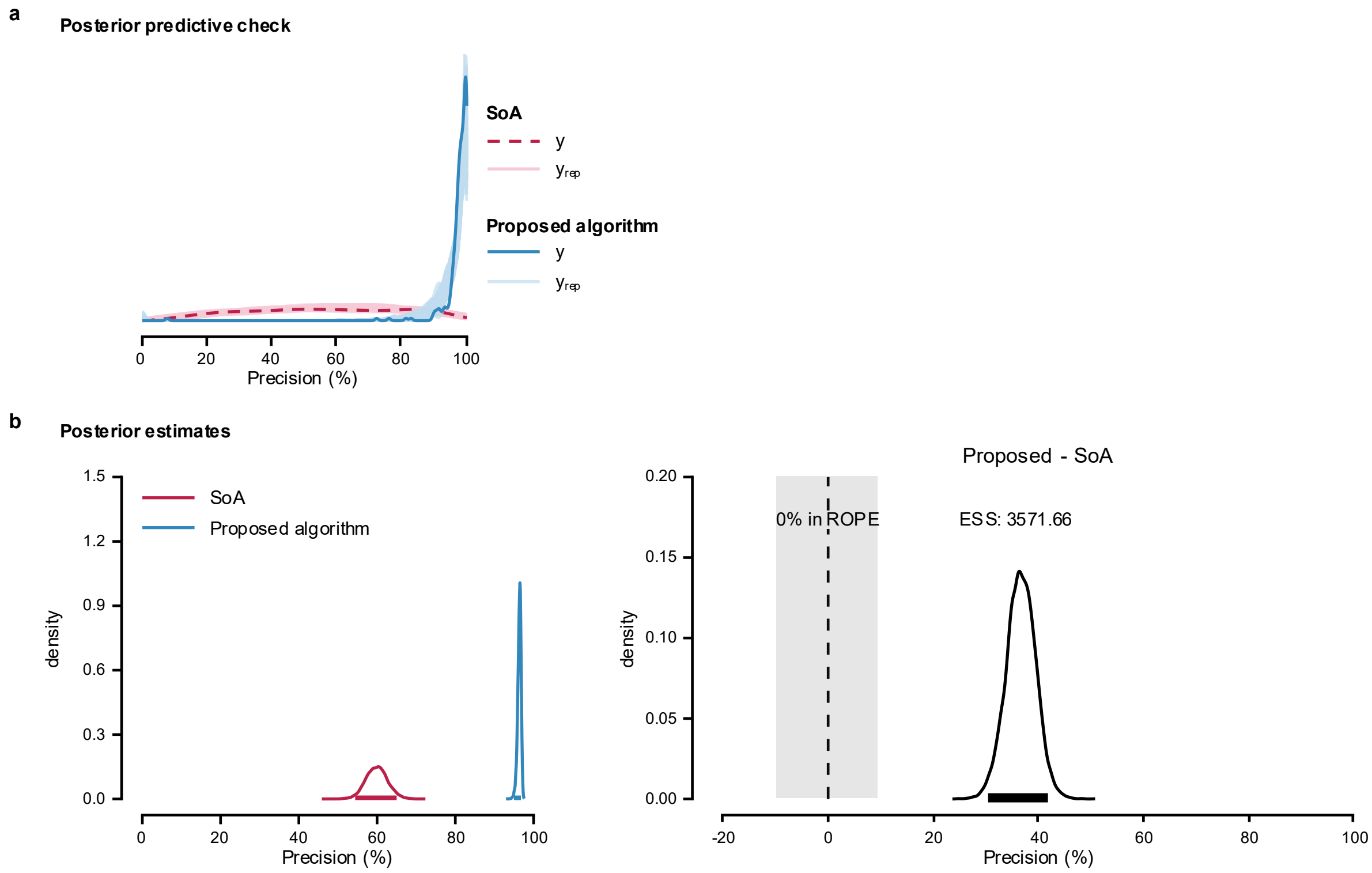

Supplementary Fig. 25: Posterior predictive check and posterior estimates for Model 2 of firing identification precision in tracked MG MUs across synthetic grid displacement. **a**, Posterior predictive check of the model; **b**, Posterior distributions and the difference between means estimated using Bayesian Model 2 to compare the performance of the proposed algorithm and SoA method.

<u>Model 3</u>

The maximum PSRF across all parameters was 1.003, indicating good convergence, while the minimum ESS was 3,675.855, indicating stable estimates of the bounds of the credible intervals (Supplementary Table 35). Posterior summaries are reported as medians with 95% ETIs and 95% HDIs (Supplementary Table 36). Group-level correlations are reported on the correlation scale.

Posterior predictive checks were performed by overlaying model-generated predictions on the observed data (Supplementary Fig. 26a). The posterior distributions (Supplementary Fig.

26b) indicated that the proposed algorithm achieved a higher precision (95.2%, [94.4-95.8%]) than the SoA algorithm (29.5%, [26.0-33.1%]). The posterior distribution of the difference excluded zero, and less than 2.5% of its distribution fell within ROPE.

Supplementary Table 35: Posterior MCMC diagnostics for Model 3 of firing identification precision in tracked MG MUs across synthetic grid displacement.

	**PSRF Pt**	**PSRF UpCI**	**ESS**
α_{μ}	1.001	1.003	6,707.515
α_{ϕ}	1.000	1.000	1,3539.405
α_{ζ}	1.000	1.001	1,4119.397
α_{κ}	1.000	1.000	2,1987.004
$\beta_{\mu,\text{Proposed}}$	1.001	1.002	7,261.005
$\beta_{\phi,\text{Proposed}}$	1.000	1.001	3,675.855
$\beta_{\zeta,\text{Proposed}}$	1.000	1.001	1,4193.201
$\beta_{\kappa,\text{Proposed}}$	1.000	1.000	1,9256.450
τ_{b0}	1.000	1.001	6,586.077
$\tau_{b\text{Proposed}}$	1.000	1.000	6,752.279
τ_{c}	1.000	1.001	4,099.645
R_{12}	1.001	1.003	5,109.176

1079 **Supplementary Table 36: Posterior summaries for Model 3 of firing identification**

1080 **precision in tracked MG MUs across synthetic grid displacement.**

	**50%**	**95% ETI**		**Mode**	**95% HDI**	
		**2.5%**	**97.5%**		**low**	**high**
α_{μ}	0.605	0.542	0.666	0.604	0.545	0.669
α_{ϕ}	5.556	4.550	6.774	5.475	4.475	6.687
α_{ζ}	0.006	0.001	0.018	0.004	0.000	0.016
α_{κ}	0.169	0.007	0.856	0.043	0.001	0.775
$\beta_{\mu,\text{Proposed}}$	24.856	17.430	35.033	23.991	16.691	33.977
$\beta_{\phi,\text{Proposed}}$	17.705	10.757	30.779	16.286	9.877	28.974
$\beta_{\zeta,\text{Proposed}}$	40.809	12.599	201.477	25.376	6.692	152.015
$\beta_{\kappa,\text{Proposed}}$	287.547	5.805	1,9533.890	-303.170	0.149	9,755.657
τ_{b0}	0.505	0.338	0.749	0.487	0.322	0.722
$\tau_{b\text{Proposed}}$	0.712	0.514	1.017	0.692	0.499	0.989
τ_{c}	0.632	0.533	0.734	0.625	0.529	0.730
R_{12}	-0.773	-0.933	-0.421	-0.825	-0.961	-0.482

1081

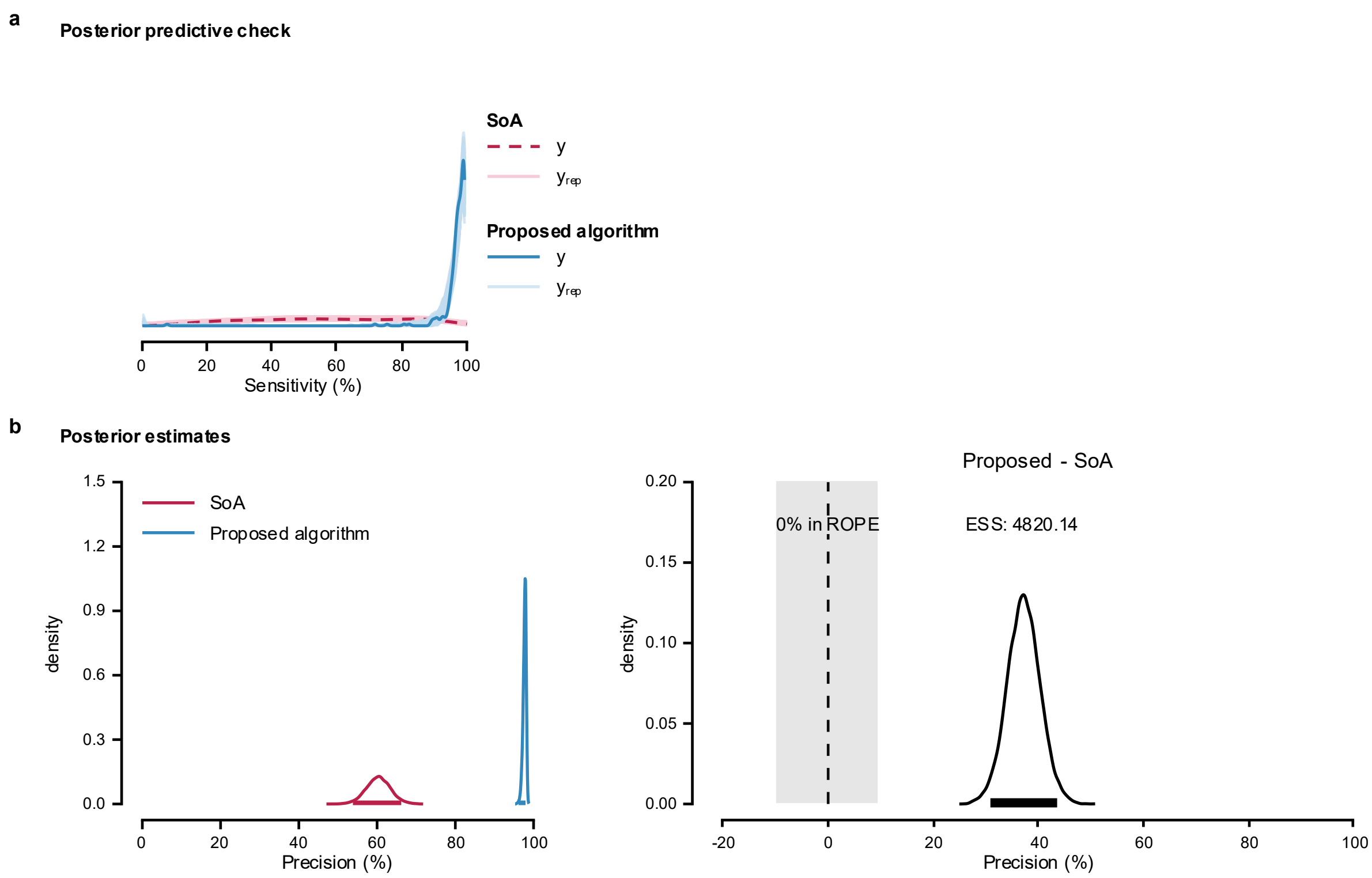

Supplementary Fig. 26: Posterior predictive check and posterior estimates for Model 3 of firing identification precision in tracked MG MUs across synthetic grid displacement. **a**, Posterior predictive check of the model; **b**, Posterior distributions and the difference between means estimated using Bayesian Model 1 to compare the performance of the proposed algorithm and SoA method.

MODEL COMPARISON

Model 2 had the highest expected predictive accuracy. Relative to Model 2, the ELPD diff were −7.5 (SE = 15.1) for Model 3 and −17.6 (SE = 14.6) for Model 1. As both differences were small relative to their uncertainty, the models showed no clear differences in out-of-sample predictive performance, although the point estimates favored Model 2.

Supplementary Note 12. Comparison of firing identification PNR for tracked TA MUs across testing visits.

Model implementation, likelihood function, and priors are detailed in Supplementary Note 5. The full dataset contains firing identification PNR values for 119 tracked MUs in TA muscle (Supplementary Fig. 27). The MUs firings were obtained by applying the MU filters calculated using the SoA algorithm[5] and the proposed algorithm to the HDsEMG signals obtained in the second testing visit.

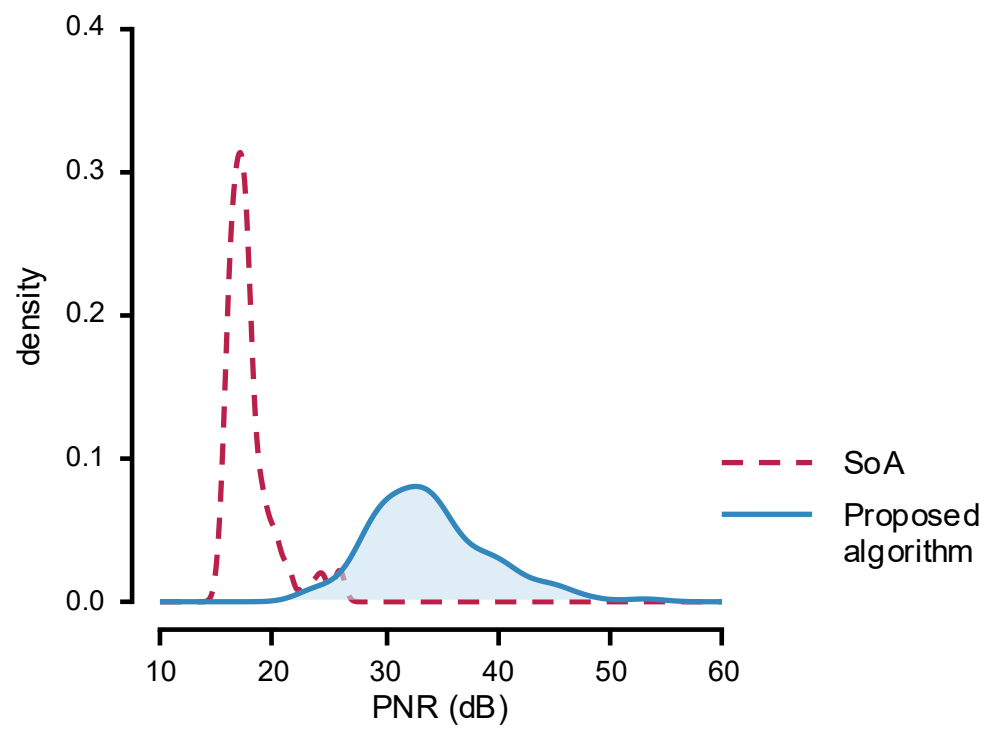

Supplementary Fig. 27: Distribution of firing identification PNR in tracked TA MUs across testing visits.

PNR values were calculated from firing identification obtained by applying MU filters calculated using SoA (dashed line) and the proposed algorithms (solid line) to the HDsEMG signals recorded in the second testing visit.

In all models, the lognormal mean and sigma effects were exponentiated before the posterior summaries were calculated. Group-level SDs are reported on their native model or link scales.

Model 1

The maximum PSRF across all parameters was 1.002, indicating good convergence, while the minimum ESS was 4,130.150, indicating stable estimates of the bounds of the credible

intervals (Supplementary Table 37). Median of the estimate value in the original scale of PNR, the limits of the 95% ETI, and the limits of the 95% HDI are reported in Supplementary Table 38.

Supplementary Table 37: Posterior MCMC diagnostics for Model 1 of firing identification PNR in tracked TA MUs across testing visits.

	**PSRF Pt**	**PSRF UpCI**	**ESS**
α_{μ}	1.001	1.002	4,130.150
α_{σ}	1.000	1.000	1,2408.010
$\beta_{\mu,\text{Proposed}}$	1.000	1.000	2,0473.389
$\beta_{\sigma,\text{Proposed}}$	1.000	1.000	1,4264.345
τ_{μ}	1.001	1.001	5,003.463

Supplementary Table 38: Posterior summaries for Model 1 of firing identification PNR in tracked TA MUs across testing visits.

	**50%**	**95% ETI**		**Mode**	**95% HDI**	
		**2.5%**	**97.5%**		**low**	**high**
α_{μ}	17.948	17.013	18.959	17.973	16.987	18.926
α_{σ}	1.871	1.811	1.935	1.867	1.811	1.935
$\beta_{\mu,\text{Proposed}}$	0.080	0.069	0.094	0.080	0.068	0.093
$\beta_{\sigma,\text{Proposed}}$	2.063	1.665	2.541	2.030	1.638	2.509
τ_{μ}	0.083	0.051	0.142	0.074	0.047	0.133

Posterior predictive checks were performed by overlaying model-generated predictions on the observed data (Supplementary Fig. 28a). The posterior distributions (Supplementary Fig. 28b) indicated that the proposed algorithm achieved a higher PNR (34.1 dB, [32.1-36.2 dB]) than the SoA algorithm (18.0 dB, [17.1-19.0 dB]). The posterior distribution of the difference excluded zero, and less than 2.5% of its distribution fell within ROPE.

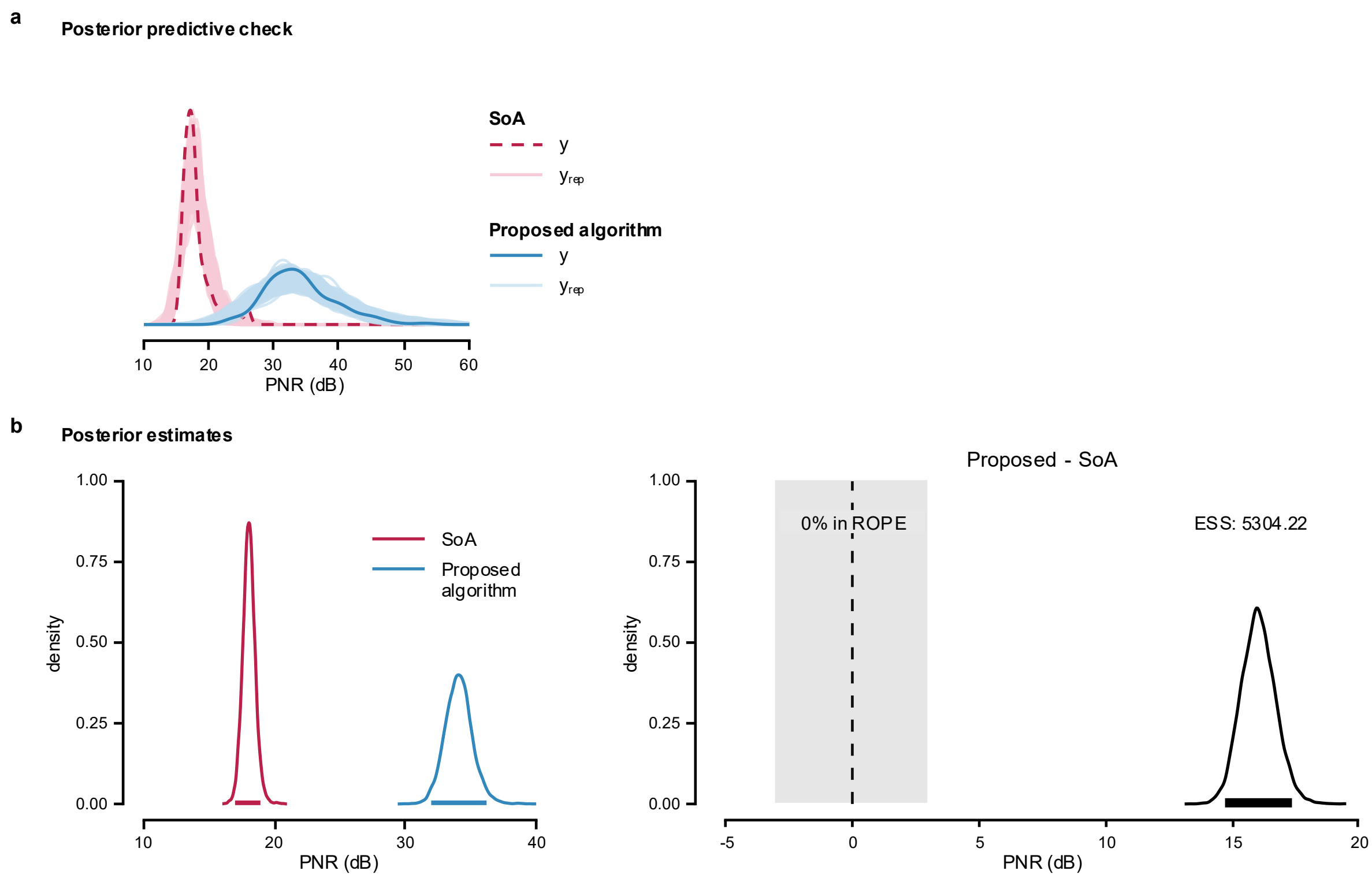

Supplementary Fig. 28: Posterior predictive check and posterior estimates for Model 1 of firing identification PNR in tracked TA MUs across testing visits.

a, Posterior predictive check of the model; **b**, Posterior distributions and the difference between means estimated using Bayesian Model 1 to compare the performance of the proposed algorithm and SoA method.

Model 2

The maximum PSRF across all parameters was 1.003, indicating good convergence, while the minimum ESS was 4,523.017, indicating stable estimates of the bounds of the credible intervals (Supplementary Table 39). Posterior estimates are summarized by the median, 95% ETIs and 95% HDIs (Supplementary Table 40). Group-level correlations were retained on their native scales.

Posterior predictive checks were performed by overlaying model-generated predictions on the observed data (Supplementary Fig. 29a). The posterior distributions (Supplementary Fig. 29b) indicated that the proposed algorithm achieved a higher PNR (33.2 dB, [31.0-35.0 dB]) than the SoA algorithm (18.3 dB, [17.2-19.6 dB]). The posterior distribution of the difference excluded zero, and less than 2.5% of its distribution fell within ROPE.

Model 3

The maximum PSRF across all parameters was 1.006, indicating good convergence, while the minimum ESS was 4,930.471, indicating stable estimates of the bounds of the credible intervals (Supplementary Table 41). Correlations were summarized on their native scales and values are reported as medians with 95% ETIs and 95% HDIs (Supplementary Table 42).

Posterior predictive checks were performed by overlaying model-generated predictions on the observed data (Supplementary Fig. 30a). The posterior distributions (Supplementary Fig. 30b) indicated that the proposed algorithm achieved a higher PNR (33.1 dB, [31.0-35.0 dB]) than the SoA algorithm (18.3 dB, [17.3-19.5 dB]). The posterior distribution of the difference excluded zero, and less than 2.5% of its distribution fell within ROPE.

Supplementary Table 39: Posterior MCMC diagnostics for Model 2 of firing identification PNR in tracked TA MUs across testing visits.

	PSRF Pt	PSRF UpCI	ESS
α_{μ}	1.000	1.001	4,523.017
α_{σ}	1.000	1.000	1,7991.923
$\beta_{\mu,\text{Proposed}}$	1.000	1.000	5,467.359
$\beta_{\sigma,\text{Proposed}}$	1.000	1.000	1,9290.546
τ_{b0}	1.001	1.003	5,921.262
$\tau_{b\text{Proposed}}$	1.000	1.001	7,009.741
R_{12}	1.000	1.001	8,687.863

Supplementary Table 40: Posterior summaries for Model 2 of firing identification PNR in tracked TA MUs across testing visits.

	50%	95% ETI		Mode	95% HDI	
		2.5%	97.5%		low	high
α_{μ}	18.216	17.183	19.487	18.146	17.115	19.418
α_{σ}	0.074	0.065	0.085	0.073	0.065	0.085
$\beta_{\mu,\text{Proposed}}$	1.802	1.636	1.947	1.798	1.646	1.955
$\beta_{\sigma,\text{Proposed}}$	1.917	1.600	2.288	1.914	1.585	2.266
τ_{b0}	0.096	0.064	0.156	0.088	0.059	0.147
$\tau_{b\text{Proposed}}$	0.122	0.075	0.208	0.117	0.069	0.195
R_{12}	-0.794	-0.965	-0.297	-0.866	-0.987	-0.395

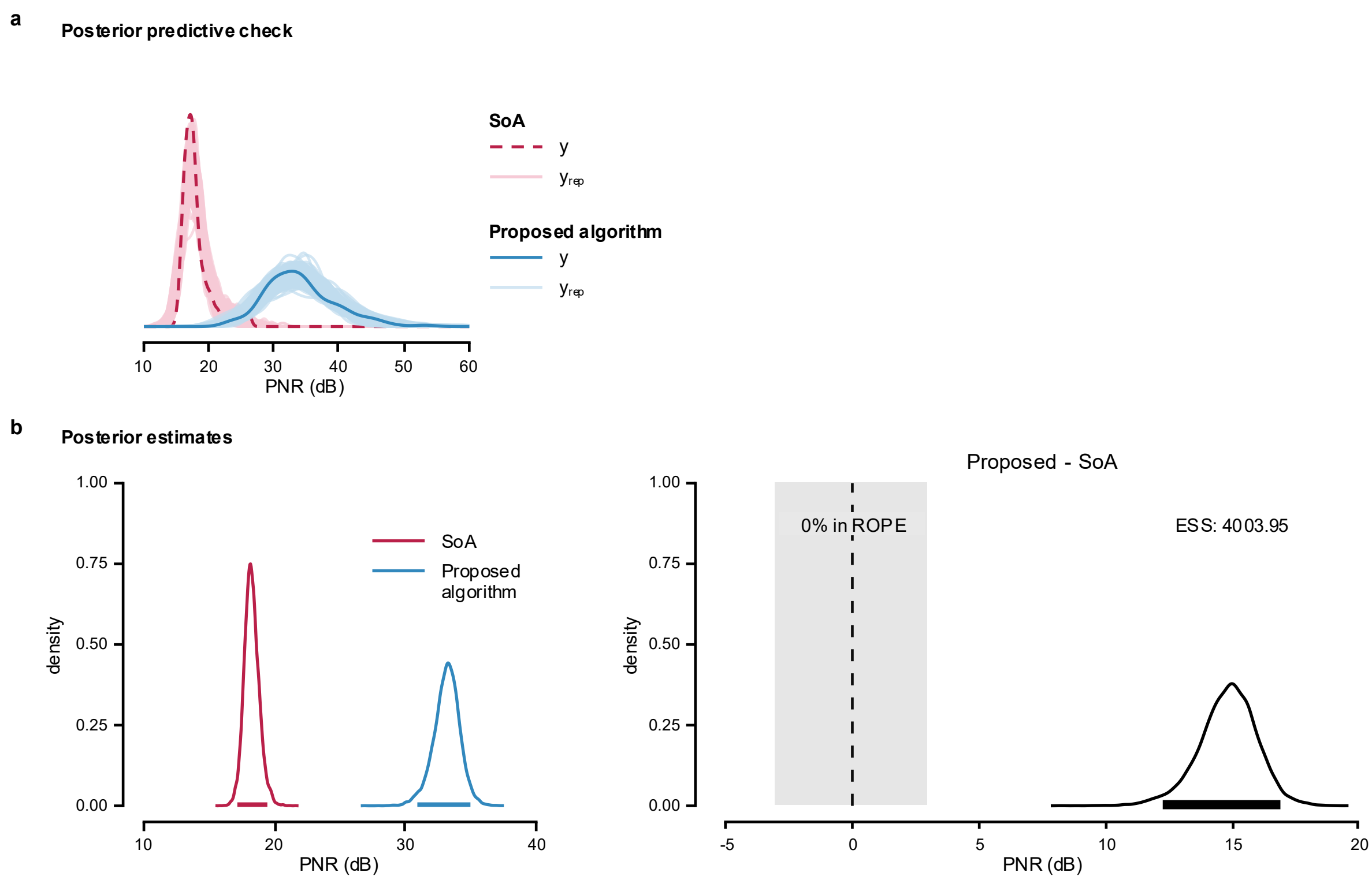

Supplementary Fig. 29: Posterior predictive check and posterior estimates for Model 2 of firing identification PNR in tracked TA MUs across testing visits.

a, Posterior predictive check of the model; **b**, Posterior distributions and the difference in means from Bayesian Model 2, used to compare the performance of the proposed algorithm and SoA method.

Supplementary Table 41: Posterior MCMC diagnostics for Model 3 of firing identification PNR in tracked TA MUs across testing visits.

	PSRF Pt	PSRF UpCI	ESS
α_{μ}	1.000	1.001	4,930.471
α_{σ}	1.000	1.000	2,0872.561
$\beta_{\mu,\text{Proposed}}$	1.000	1.001	6,970.288
$\beta_{\sigma,\text{Proposed}}$	1.000	1.000	2,5687.106
τ_{b0}	1.000	1.002	7,382.935
$\tau_{b\text{Proposed}}$	1.001	1.003	7,809.601
τ_{c}	1.002	1.006	8,923.323
R_{12}	1.000	1.000	9,823.605

Supplementary Table 42: Posterior summaries for Model 3 of firing identification PNR in tracked TA MUs across testing visits.

	50%	95% ETI		Mode	95% HDI	
		2.5%	97.5%		low	high
α_{μ}	18.229	17.211	19.462	18.170	17.176	19.426
α_{σ}	0.074	0.064	0.085	0.073	0.064	0.085
$\beta_{\mu,\text{Proposed}}$	1.801	1.637	1.941	1.802	1.647	1.950
$\beta_{\sigma,\text{Proposed}}$	1.929	1.601	2.316	1.912	1.577	2.289
τ_{b0}	0.096	0.064	0.157	0.088	0.061	0.149
$\tau_{b\text{Proposed}}$	0.122	0.075	0.210	0.114	0.069	0.199
τ_{c}	0.010	0.000	0.030	0.003	0.000	0.026
R_{12}	-0.792	-0.965	-0.278	-0.880	-0.986	-0.376

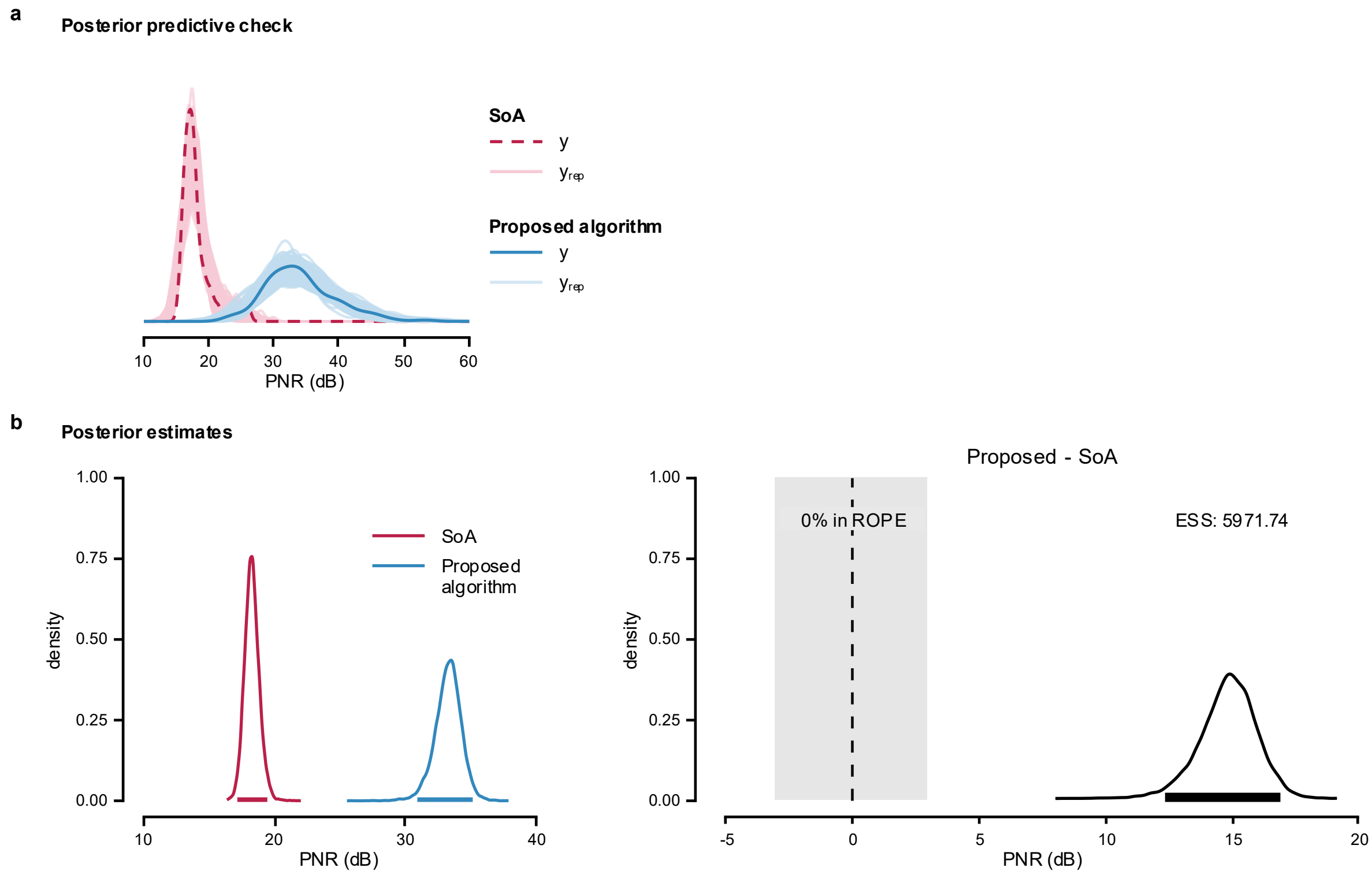

Supplementary Fig. 30: Posterior predictive check and posterior estimates for Model 3 of firing identification PNR in tracked TA MUs across testing visits.

a, Posterior predictive check of the model; **b**, Posterior distributions and the difference in means from Bayesian Model 3, used to compare the performance of the proposed algorithm and SoA method.

MODEL COMPARISON

Model 2 showed the greatest expected predictive accuracy. ELPD diff relative to Model 2 were −0.9 (SE = 0.3) for Model 3 and −22.5 (SE = 7.6) for Model 1, suggesting similar predictive performance for Models 2 and 3 and reduced performance for Model 1.

Supplementary Note 13. Comparison of firing identification sensitivity in tracked TA MUs across testing visits.

Model implementation, likelihood function, and priors are detailed in Supplementary Note 5. The full dataset contains firing identification sensitivity values for 119 tracked TA MUs (hereafter referred to as 'sensitivity'). The MUs firings were obtained by applying the MU filters calculated using the SoA algorithm[5] and the proposed algorithm to the HDsEMG signals obtained in the second testing visit. Distribution of the data is shown in Supplementary Fig. 31.

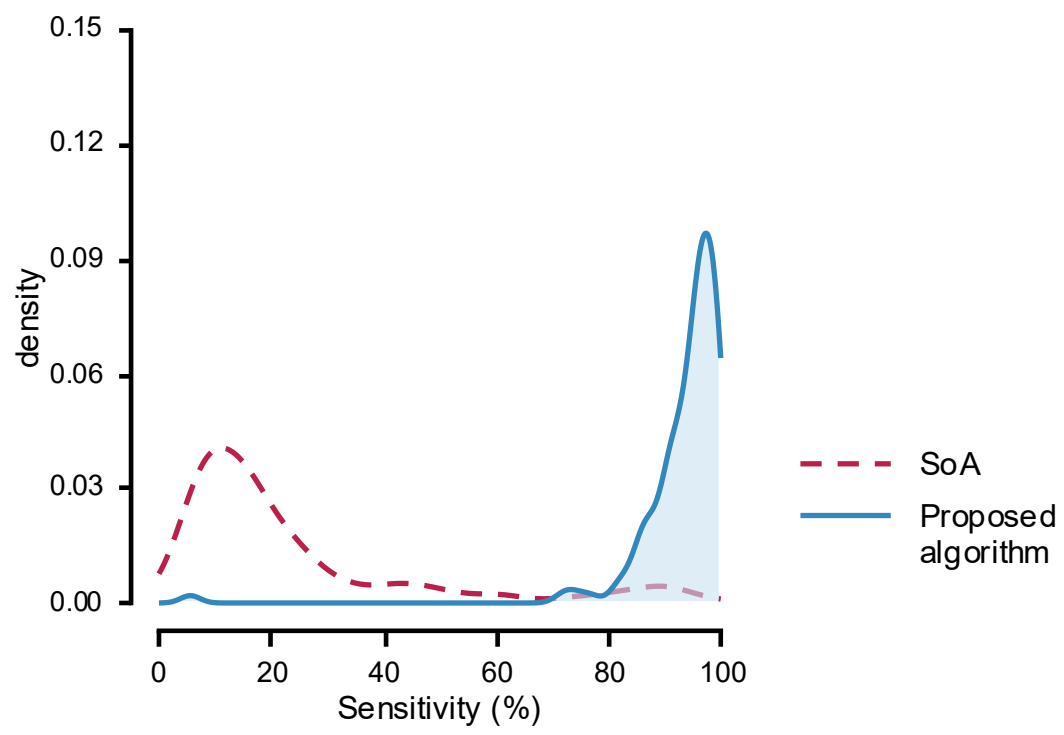

Supplementary Fig. 31: Distribution of firing identification sensitivity in tracked TA MUs across testing visits.

Sensitivity values were calculated from firing identification obtained by applying MU filters calculated using SoA (dashed line) and the proposed algorithms (solid line) to the HDsEMG signals generated with synthetic grid displacement.

In all models, ZOIB parameters were back transformed into their interpretable component scales: *mu*, *zoi*, and *coi* to probabilities, and *phi* to the positive precision scale. Group-level SDs are reported on their native model/link scale.

Model 1

The maximum PSRF across all parameters was 1.003, indicating good convergence, while the minimum ESS was 4,215.681, indicating stable estimates of the bounds of the credible intervals (Supplementary Table 43). Posterior summaries are reported in Supplementary Table 44 as medians with 95% ETIs and the 95% HDIs.

Posterior predictive checks were performed by overlaying model-generated predictions on the observed data (Supplementary Fig. 32a). The posterior distributions (Supplementary Fig. 32b) indicated that the proposed algorithm achieved a higher sensitivity (92.0%, [87.5-95.1%]) than the SoA algorithm (22.5%, [14.6-31.6%]). The posterior distribution of the difference excluded zero, and less than 2.5% of its distribution fell within ROPE.

Supplementary Table 43: Posterior MCMC diagnostics for Model 1 of firing identification sensitivity in tracked TA MUs across testing visits.

	**PSRF Pt**	**PSRF UpCI**	**ESS**
α_{μ}	1.001	1.003	4,215.681
α_{ϕ}	1.000	1.001	1,5084.716
α_{ζ}	1.000	1.001	1,0663.543
α_{κ}	1.000	1.000	1,4091.757
$\beta_{\mu,\text{Proposed}}$	1.000	1.001	1,5437.385
$\beta_{\phi,\text{Proposed}}$	1.000	1.001	1,6987.396
$\beta_{\zeta,\text{Proposed}}$	1.000	1.001	1,1797.238
$\beta_{\kappa,\text{Proposed}}$	1.000	1.000	1,5458.274
τ_b	1.000	1.001	5,863.542

Supplementary Table 44: Posterior summaries for Model 1 of firing identification sensitivity in tracked TA MUs across testing visits.

	50%	95% ETI		Mode	95% HDI	
		2.5%	97.5%		low	high
α_{μ}	0.223	0.144	0.318	0.223	0.141	0.312
α_{ϕ}	9.623	7.063	12.692	9.481	6.984	12.578
α_{ζ}	0.014	0.003	0.042	0.009	0.001	0.036
α_{κ}	0.248	0.011	0.900	0.054	0.001	0.845
$\beta_{\mu,\text{Proposed}}$	41.576	31.925	53.373	41.717	31.163	52.277
$\beta_{\phi,\text{Proposed}}$	1.023	0.670	1.556	0.968	0.644	1.509
$\beta_{\zeta,\text{Proposed}}$	8.266	2.200	46.386	5.258	1.005	34.951
$\beta_{\kappa,\text{Proposed}}$	48.988	0.818	4,662.301	-59.101	0.009	2,232.798
τ_{b}	0.804	0.522	1.351	0.755	0.475	1.263

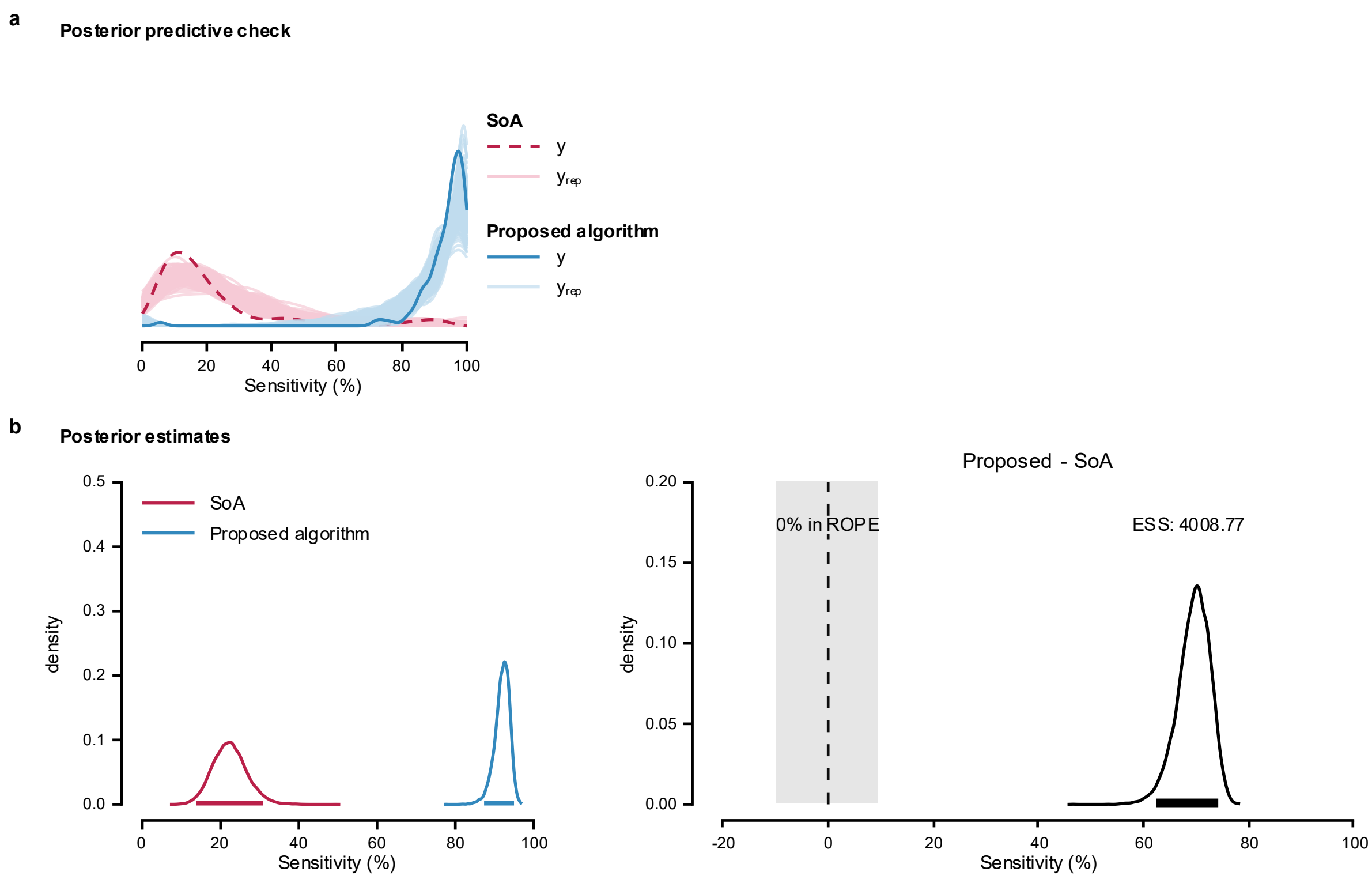

Supplementary Fig. 32: Posterior predictive check and posterior estimates for Model 1 of firing identification sensitivity in tracked TA MUs across testing visits.

a, Posterior predictive check of the model; **b**, Posterior distributions and the difference between means estimated using Bayesian Model 1 to compare the performance of the proposed algorithm and SoA method.

Model 2

The maximum PSRF across all parameters was 1.001, indicating good convergence, while the minimum ESS was 5,341.062, indicating stable estimates of the bounds of the credible intervals (Supplementary Table 45). Medians with 95% ETIs and 95% HDIs are reported in Supplementary Table 46. Group-level correlations are reported on the correlation scale.

Posterior predictive checks were performed by overlaying model-generated predictions on the observed data (Supplementary Fig. 33a). The posterior distributions (Supplementary Fig. 33b) indicated that the proposed algorithm achieved a higher sensitivity (91.3%, [88.1-

93.7%]) than the SoA algorithm (23.5%, [15.1-33.5%]). The posterior distribution of the difference excluded zero, and less than 2.5% of its distribution fell within ROPE.

Supplementary Table 45: Posterior MCMC diagnostics for Model 2 of firing identification sensitivity in tracked TA MUs across testing visits.

	PSRF Pt	PSRF UpCI	ESS
α_{μ}	1.000	1.001	5,341.062
α_{ϕ}	1.000	1.000	2,3605.135
α_{ζ}	1.000	1.001	1,5785.997
α_{κ}	1.000	1.000	2,0488.653
$\beta_{\mu,\text{Proposed}}$	1.000	1.001	6,985.508
$\beta_{\phi,\text{Proposed}}$	1.000	1.000	2,4487.073
$\beta_{\zeta,\text{Proposed}}$	1.000	1.001	1,6925.057
$\beta_{\kappa,\text{Proposed}}$	1.000	1.000	2,0792.586
τ_{b0}	1.000	1.000	8,042.371
$\tau_{b\text{Proposed}}$	1.000	1.000	1,0041.826
R_{12}	1.000	1.001	1,1376.206

Supplementary Table 46: Posterior summaries for Model 2 of firing identification sensitivity in tracked TA MUs across testing visits.

	50%	95% ETI		Mode	95% HDI	
		2.5%	97.5%		low	high
α_{μ}	0.230	0.149	0.335	0.229	0.147	0.332
α_{ϕ}	11.018	8.326	14.181	10.979	8.147	13.941
α_{ζ}	0.013	0.003	0.042	0.009	0.001	0.036
α_{κ}	0.248	0.012	0.905	0.056	0.001	0.850
$\beta_{\mu,\text{Proposed}}$	35.187	20.475	59.348	33.674	18.915	56.528
$\beta_{\phi,\text{Proposed}}$	0.929	0.628	1.377	0.883	0.595	1.324
$\beta_{\zeta,\text{Proposed}}$	8.332	2.159	47.273	4.762	1.042	35.144
$\beta_{\kappa,\text{Proposed}}$	49.394	0.784	4,082.842	-57.578	0.004	1,963.511
τ_{b0}	0.842	0.583	1.302	0.800	0.546	1.240
$\tau_{b\text{Proposed}}$	0.752	0.461	1.225	0.703	0.433	1.175
R_{12}	-0.920	-0.992	-0.579	-0.967	-0.999	-0.657

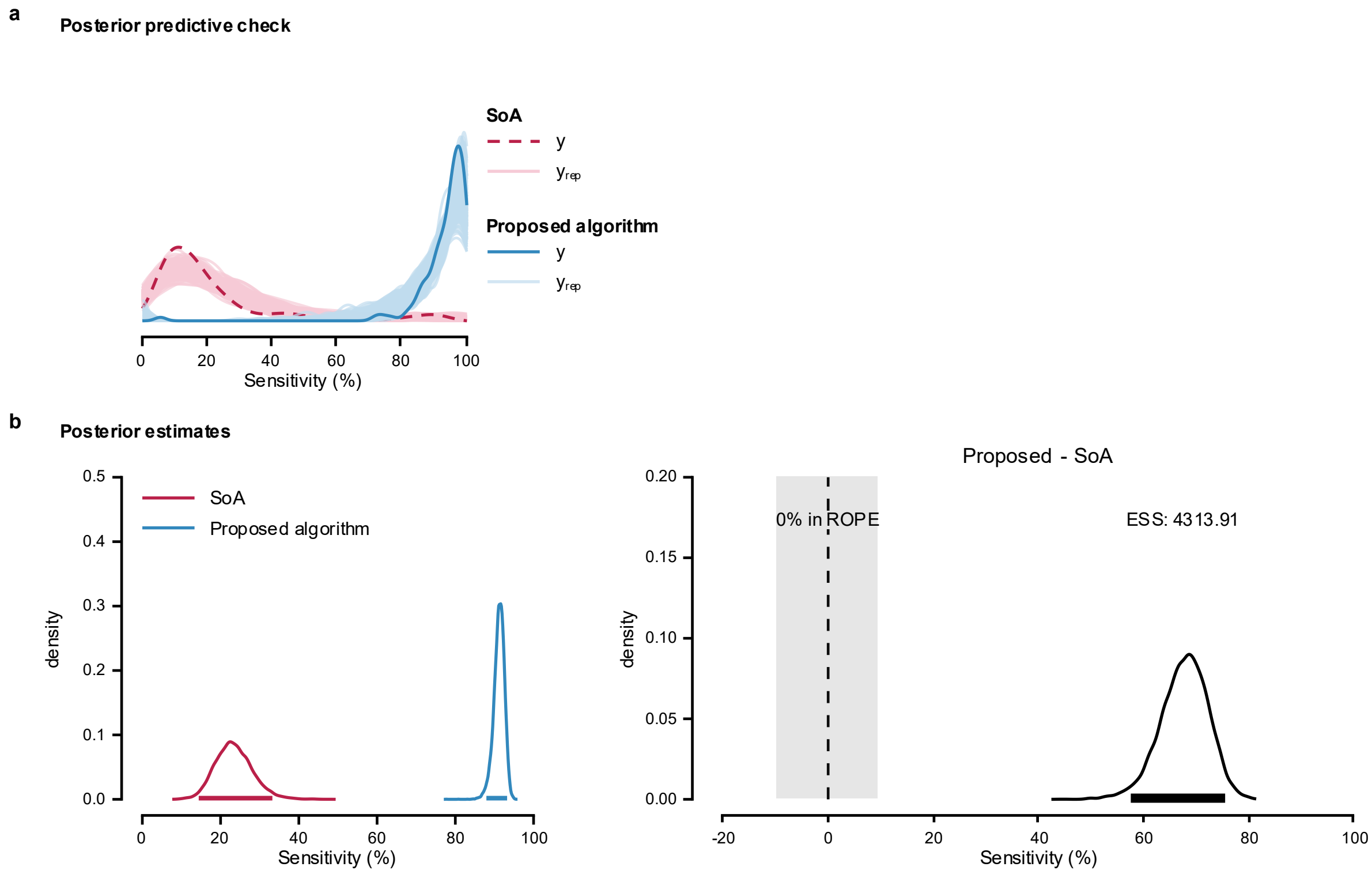

Supplementary Fig. 33: Posterior predictive check and posterior estimates for Model 2 of firing identification sensitivity in tracked TA MUs across testing visits.

a, Posterior predictive check of the model; **b**, Posterior distributions and the difference in means from Bayesian Model 2, used to compare the performance of the proposed algorithm and SoA method.

<u>Model 3</u>

The maximum PSRF across all parameters was 1.005, indicating good convergence, while the minimum ESS was 3,962.626, indicating stable estimates of the bounds of the credible intervals (Supplementary Table 47). Posterior parameter summaries are given as medians with 95% ETIs and 95% HDIs (Supplementary Table 48). Correlations were summarized without transformation.

Posterior predictive checks were performed by overlaying model-generated predictions on the observed data (Supplementary Fig. 34a). The posterior distributions (Supplementary Fig.

34b) indicated that the proposed algorithm achieved a higher sensitivity (91.3%, [88.1-93.7%]) than the SoA algorithm (23.4%, [14.7-33.6%]). The posterior distribution of the difference excluded zero, and less than 2.5% of its distribution fell within ROPE.

Supplementary Table 47: Posterior MCMC diagnostics for Model 3 of firing identification sensitivity in tracked TA MUs across testing visits.

	PSRF Pt	PSRF UpCI	ESS
α_{μ}	1.002	1.005	3,962.626
α_{ϕ}	1.000	1.000	2,9278.024
α_{ζ}	1.000	1.000	1,6426.892
α_{κ}	1.000	1.000	2,5438.194
$\beta_{\mu,\text{Proposed}}$	1.001	1.003	5,509.277
$\beta_{\phi,\text{Proposed}}$	1.000	1.000	3,1206.007
$\beta_{\zeta,\text{Proposed}}$	1.000	1.000	1,7555.071
$\beta_{\kappa,\text{Proposed}}$	1.000	1.000	2,6403.029
τ_{b0}	1.000	1.001	7,358.389
$\tau_{b\text{Proposed}}$	1.000	1.000	9,447.571
τ_{c}	1.000	1.000	1,2668.569
R_{12}	1.000	1.000	1,1599.114

Supplementary Table 48: Posterior summaries for Model 3 of firing identification sensitivity in tracked TA MUs across testing visits.

	50%	95% ETI		Mode	95% HDI	
		2.5%	97.5%		low	high
α_{μ}	0.230	0.146	0.337	0.233	0.143	0.332
α_{ϕ}	11.141	8.424	14.418	11.043	8.278	14.257
α_{ζ}	0.013	0.003	0.043	0.009	0.001	0.037
α_{κ}	0.253	0.012	0.907	0.055	0.001	0.852
$\beta_{\mu,\text{Proposed}}$	35.258	20.413	60.053	33.004	18.812	56.833
$\beta_{\phi,\text{Proposed}}$	0.922	0.613	1.367	0.911	0.580	1.320
$\beta_{\zeta,\text{Proposed}}$	8.261	2.132	48.618	4.999	0.848	35.860
$\beta_{\kappa,\text{Proposed}}$	47.391	0.763	4,531.740	-56.369	0.015	2,054.125
τ_{b0}	0.837	0.578	1.298	0.793	0.553	1.246
$\tau_{b\text{Proposed}}$	0.750	0.460	1.213	0.698	0.421	1.154
τ_{c}	0.057	0.002	0.201	0.015	0.000	0.173
R_{12}	-0.918	-0.992	-0.566	-0.967	-1.000	-0.655

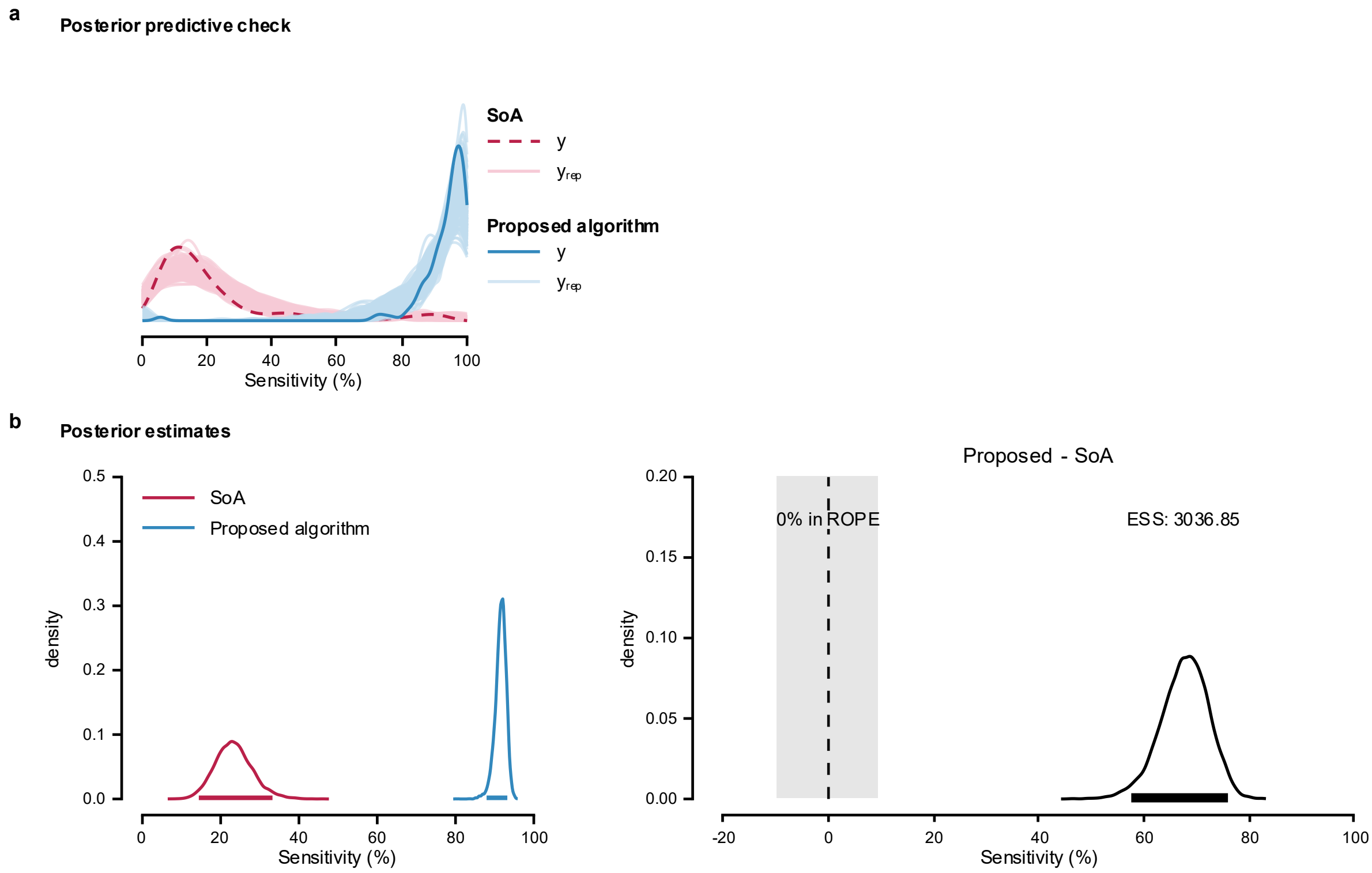

Supplementary Fig. 34: Posterior predictive check and posterior estimates for Model 3 of firing identification sensitivity in tracked TA MUs across testing visits.

a, Posterior predictive check of the model; **b**, Posterior distributions and the difference in means from Bayesian Model 3, used to compare the performance of the proposed algorithm and SoA method.

MODEL COMPARISON

Model 2 showed the greatest expected predictive accuracy. ELPD diff relative to Model 2 were −4.0 (SE = 3.1) for Model 3 and −16.0 (SE = 5.3) for Model 1, suggesting similar predictive performance for Models 2 and 3 and reduced performance for Model 1.

Supplementary Note 14. Comparison of firing identification precision in tracked TA MUs across testing visits.

Model implementation, likelihood function, and priors are detailed in Supplementary Note 5. The full dataset contains firing identification precision values for 119 tracked MUs in TA muscle (hereafter referred to as 'precision'). The MUs firings were obtained by applying the MU filters calculated using the SoA algorithm[5] and the proposed algorithm to the HDsEMG signals obtained in the second testing visit. Distribution of the data is shown in Supplementary Fig. 35.

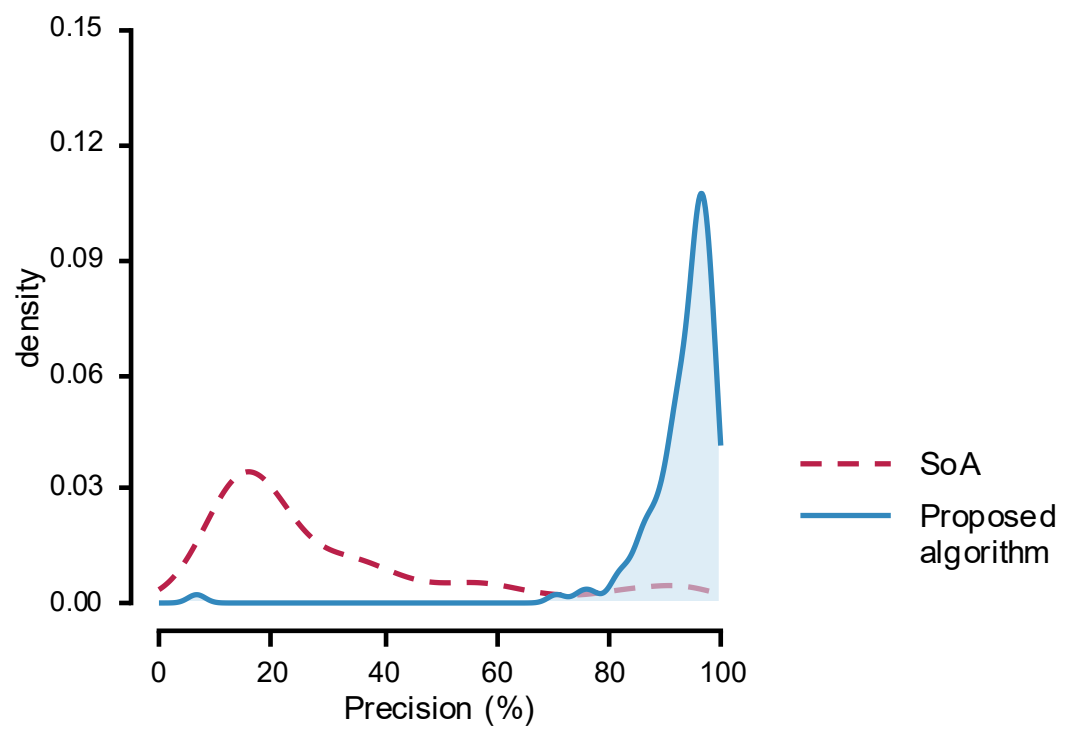

Supplementary Fig. 35: Distribution of firing identification precision in tracked TA MUs across testing visits.

Precision values were calculated from firing identification obtained by applying MU filters calculated using SoA (dashed line) and the proposed algorithms (solid line) to the HDsEMG signals generated with synthetic grid displacement.

In all models, ZOIB parameters were back transformed into their interpretable component scales: *mu*, *zoi*, and *coi* to probabilities, and *phi* to the positive precision scale. Group-level SDs are reported on their native model/link scale.

Model 1

The maximum PSRF across all parameters was 1.003, indicating good convergence, while the minimum ESS was 4,540.438, indicating stable estimates of the bounds of the credible intervals (Supplementary Table 49). Posterior summaries are reported as medians with 95% ETIs and 95% HDIs (Supplementary Table 50).

Posterior predictive checks were performed by overlaying model-generated predictions on the observed data (Supplementary Fig. 36a). The posterior distributions (Supplementary Fig. 36b) indicated that the proposed algorithm achieved a higher precision (91.2%, [87.1-94.2%]) compared to the SoA algorithm (30.3%, [22.0-39.3%]). The posterior distribution of the difference excluded zero, and less than 2.5% of its distribution fell within ROPE.

Supplementary Table 49: Posterior MCMC diagnostics for Model 1 of firing identification precision in tracked TA MUs across testing visits.

	PSRF Pt	PSRF UpCI	ESS
α_{μ}	1.001	1.002	4,540.438
α_{ϕ}	1.000	1.001	1,5449.885
α_{ζ}	1.000	1.001	1,5118.605
α_{κ}	1.000	1.001	1,6093.714
$\beta_{\mu,\text{Proposed}}$	1.000	1.001	1,5564.318
$\beta_{\phi,\text{Proposed}}$	1.000	1.000	1,8582.117
$\beta_{\zeta,\text{Proposed}}$	1.000	1.001	1,8112.688
$\beta_{\kappa,\text{Proposed}}$	1.000	1.001	1,8588.570
τ_{b}	1.001	1.003	6,051.129

Supplementary Table 50: Posterior summaries for Model 1 of firing identification precision in tracked TA MUs across testing visits.

	50%	95% ETI		Mode	95% HDI	
		2.5%	97.5%		low	high
α_{μ}	0.300	0.218	0.389	0.297	0.218	0.389
α_{ϕ}	6.870	5.046	9.047	6.860	4.960	8.930
α_{ζ}	0.015	0.003	0.045	0.010	0.001	0.039
α_{κ}	0.352	0.017	0.936	0.067	0.004	0.901
$\beta_{\mu,\text{Proposed}}$	26.418	20.655	33.566	26.016	20.231	33.048
$\beta_{\phi,\text{Proposed}}$	1.626	1.086	2.452	1.565	1.008	2.350
$\beta_{\zeta,\text{Proposed}}$	2.076	0.385	13.144	1.292	0.086	9.588
$\beta_{\kappa,\text{Proposed}}$	8.321	0.086	1,250.553	-11.953	0.001	553.799
τ_{b}	0.635	0.387	1.073	0.587	0.344	1.002

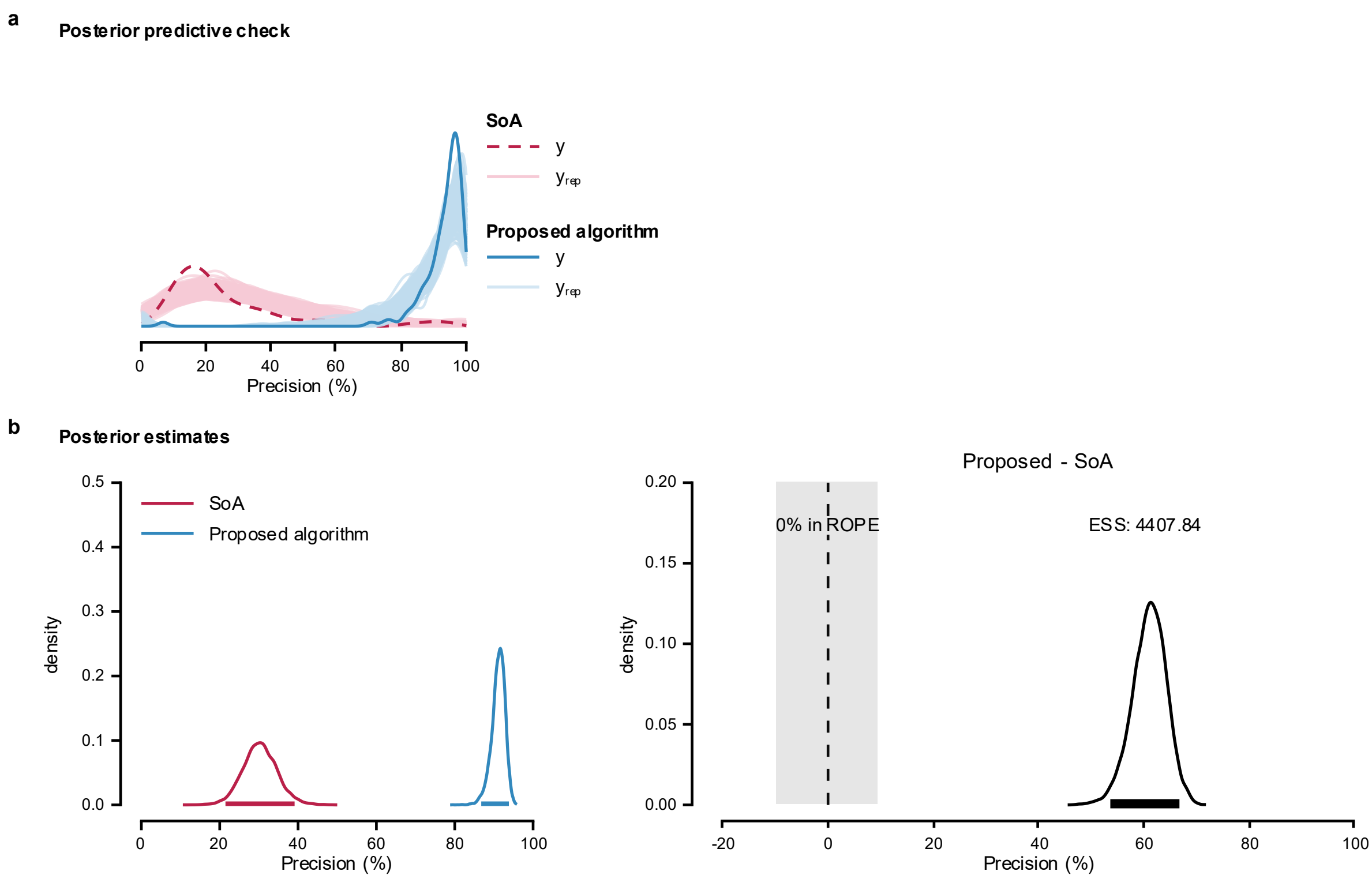

Supplementary Fig. 36: Posterior predictive check and posterior estimates for Model 1 of firing identification precision in tracked TA MUs across testing visits.

a, Posterior predictive check of the model; **b**, Posterior distributions and the difference between means estimated using Bayesian Model 1 to compare the performance of the proposed algorithm and SoA method.

Model 2

The maximum PSRF across all parameters was 1.002, indicating good convergence, while the minimum ESS was 4,533.320, indicating stable estimates of the bounds of the credible intervals (Supplementary Table 51). Posterior summaries are reported in Supplementary Table 52 as medians with 95% ETIs and 95% HDIs. Group-level correlations are reported on the correlation scale.

Posterior predictive checks were performed by overlaying model-generated predictions on the observed data (Supplementary Fig. 37a). The posterior distributions (Supplementary Fig.

37b) indicated that the proposed algorithm achieved a higher precision (90.8%, [87.7-93.1%]) compared to the SoA algorithm (31.1%, [21.6-41.5%]). The posterior distribution of the difference excluded zero, and less than 2.5% of its distribution fell within ROPE.

Supplementary Table 51: Posterior MCMC diagnostics for Model 2 of firing identification precision in tracked TA MUs across testing visits.

	PSRF Pt	PSRF UpCI	ESS
α_{μ}	1.001	1.002	4,533.320
α_{ϕ}	1.000	1.000	2,0022.001
α_{ζ}	1.000	1.001	1,6363.712
α_{κ}	1.000	1.001	1,9524.266
$\beta_{\mu,\text{Proposed}}$	1.001	1.002	5,984.829
$\beta_{\phi,\text{Proposed}}$	1.000	1.000	2,0163.696
$\beta_{\zeta,\text{Proposed}}$	1.000	1.001	1,7875.132
$\beta_{\kappa,\text{Proposed}}$	1.000	1.001	2,1373.496
τ_{b0}	1.000	1.001	6,405.917
$\tau_{b\text{Proposed}}$	1.000	1.001	8,052.448
R_{12}	1.001	1.001	1,0623.779

Supplementary Table 52: Posterior summaries for Model 2 of firing identification precision in tracked TA MUs across testing visits.

	50%	95% ETI		Mode	95% HDI	
		2.5%	97.5%		low	high
α_{μ}	0.307	0.214	0.413	0.306	0.211	0.410
α_{ϕ}	8.126	6.185	10.432	7.985	6.087	10.318
α_{ζ}	0.015	0.003	0.044	0.010	0.001	0.038
α_{κ}	0.348	0.018	0.940	0.072	0.003	0.906
$\beta_{\mu,\text{Proposed}}$	24.027	14.850	38.650	22.374	14.127	37.301
$\beta_{\phi,\text{Proposed}}$	1.430	0.980	2.111	1.393	0.919	2.023
$\beta_{\zeta,\text{Proposed}}$	2.069	0.402	13.479	1.132	0.107	9.879
$\beta_{\kappa,\text{Proposed}}$	8.276	0.084	1,187.696	-11.894	0.000	542.914
τ_{b0}	0.744	0.502	1.177	0.695	0.463	1.102
$\tau_{b\text{Proposed}}$	0.658	0.387	1.079	0.615	0.348	1.020
R_{12}	-0.914	-0.991	-0.563	-0.965	-0.999	-0.644

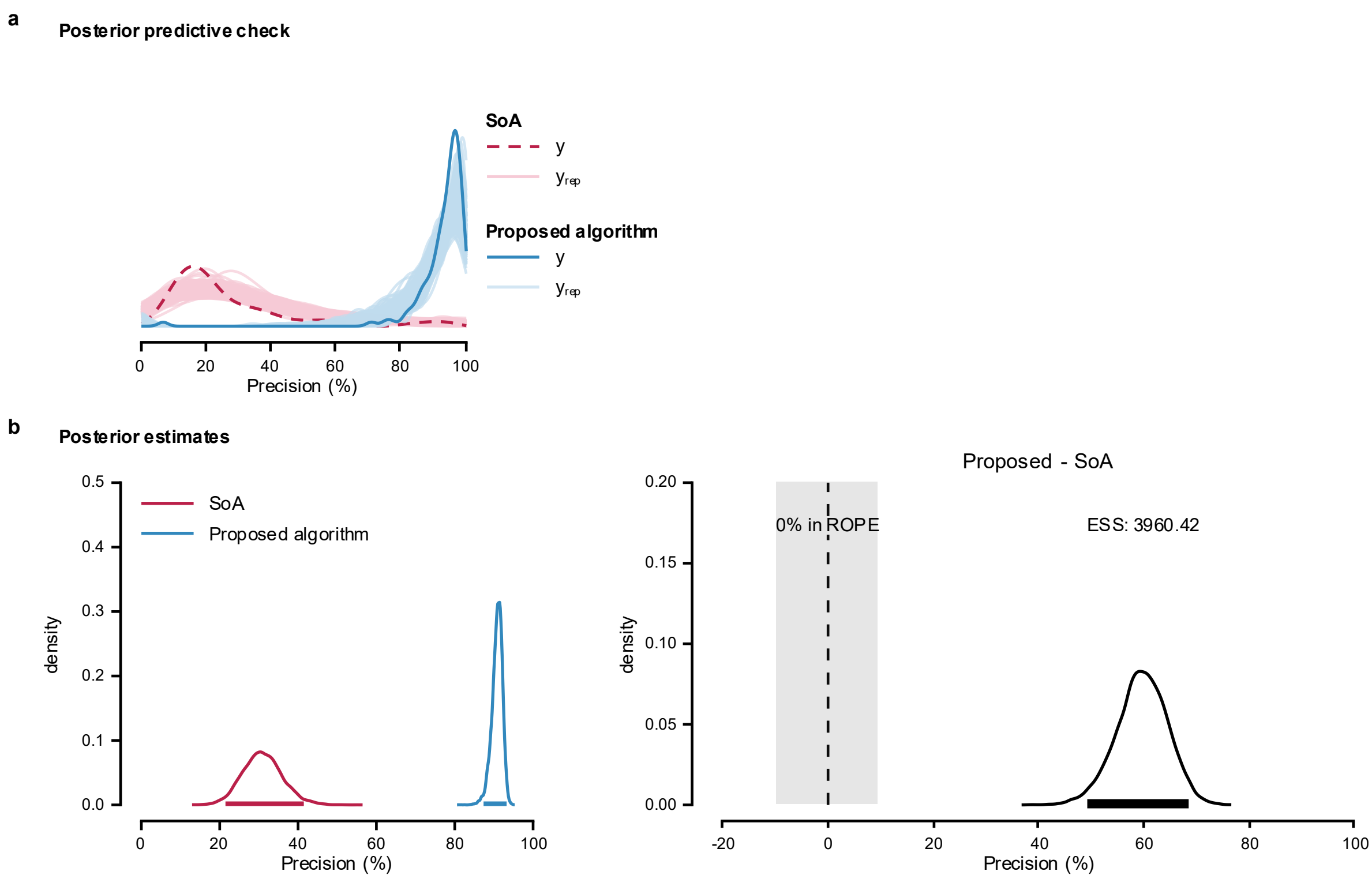

Supplementary Fig. 37: Posterior predictive check and posterior estimates for Model 2 of firing identification precision in tracked TA MUs across testing visits.

a, Posterior predictive check of the model; **b**, Posterior distributions and the difference between means estimated using Bayesian Model 2 to compare the performance of the proposed algorithm and SoA method.

<u>Model 3</u>

The maximum PSRF across all parameters was 1.006, indicating good convergence, while the minimum ESS was 3,747.211, indicating stable estimates of the bounds of the credible intervals (Supplementary Table 53). Posterior summaries are reported in Supplementary Table 54 as medians with 95% ETIs and 95% HDIs. Group-level correlations are reported on the correlation scale.

Posterior predictive checks were performed by overlaying model-generated predictions on the observed data (Supplementary Fig. 38a). The posterior distributions (Supplementary Fig.

38b) indicated that the proposed algorithm achieved a higher precision (90.8%, [87.6-93.2%]) compared to the SoA algorithm (30.8%, [21.2-41.2%]). The posterior distribution of the difference excluded zero, and less than 2.5% of its distribution fell within ROPE.

Supplementary Table 53: Posterior MCMC diagnostics for Model 3 of firing identification precision in tracked TA MUs across testing visits.

	PSRF Pt	PSRF UpCI	ESS
α_{μ}	1.002	1.006	3,747.211
α_{ϕ}	1.000	1.000	2,4548.305
α_{ζ}	1.000	1.000	1,9715.834
α_{κ}	1.000	1.000	2,3351.139
$\beta_{\mu,\text{Proposed}}$	1.001	1.004	4,987.113
$\beta_{\phi,\text{Proposed}}$	1.000	1.000	2,8334.340
$\beta_{\zeta,\text{Proposed}}$	1.000	1.000	2,4179.562
$\beta_{\kappa,\text{Proposed}}$	1.000	1.000	2,8037.912
τ_{b0}	1.000	1.001	6,566.549
$\tau_{b\text{Proposed}}$	1.000	1.001	8,418.065
τ_{c}	1.000	1.001	1,0474.429
R_{12}	1.001	1.001	1,2333.569

Supplementary Table 54: Posterior summaries for Model 3 of firing identification precision in tracked TA MUs across synthetic grid displacement.

	50%	95% ETI		Mode	95% HDI	
		2.5%	97.5%		low	high
α_{μ}	0.305	0.212	0.411	0.305	0.209	0.407
α_{ϕ}	8.202	6.210	10.639	7.988	6.118	10.519
α_{ζ}	0.015	0.003	0.043	0.010	0.001	0.038
α_{κ}	0.354	0.017	0.943	0.074	0.004	0.910
$\beta_{\mu,\text{Proposed}}$	24.273	14.967	38.998	22.776	14.044	37.212
$\beta_{\phi,\text{Proposed}}$	1.418	0.965	2.085	1.360	0.929	2.026
$\beta_{\zeta,\text{Proposed}}$	2.058	0.392	12.788	1.450	0.108	9.303
$\beta_{\kappa,\text{Proposed}}$	7.937	0.077	1,207.445	-11.383	0.001	531.034
τ_{b0}	0.745	0.506	1.169	0.716	0.478	1.119
$\tau_{b\text{Proposed}}$	0.658	0.389	1.091	0.620	0.357	1.041
τ_{c}	0.061	0.003	0.212	0.018	0.000	0.184
R_{12}	-0.915	-0.992	-0.550	-0.967	-0.999	-0.638

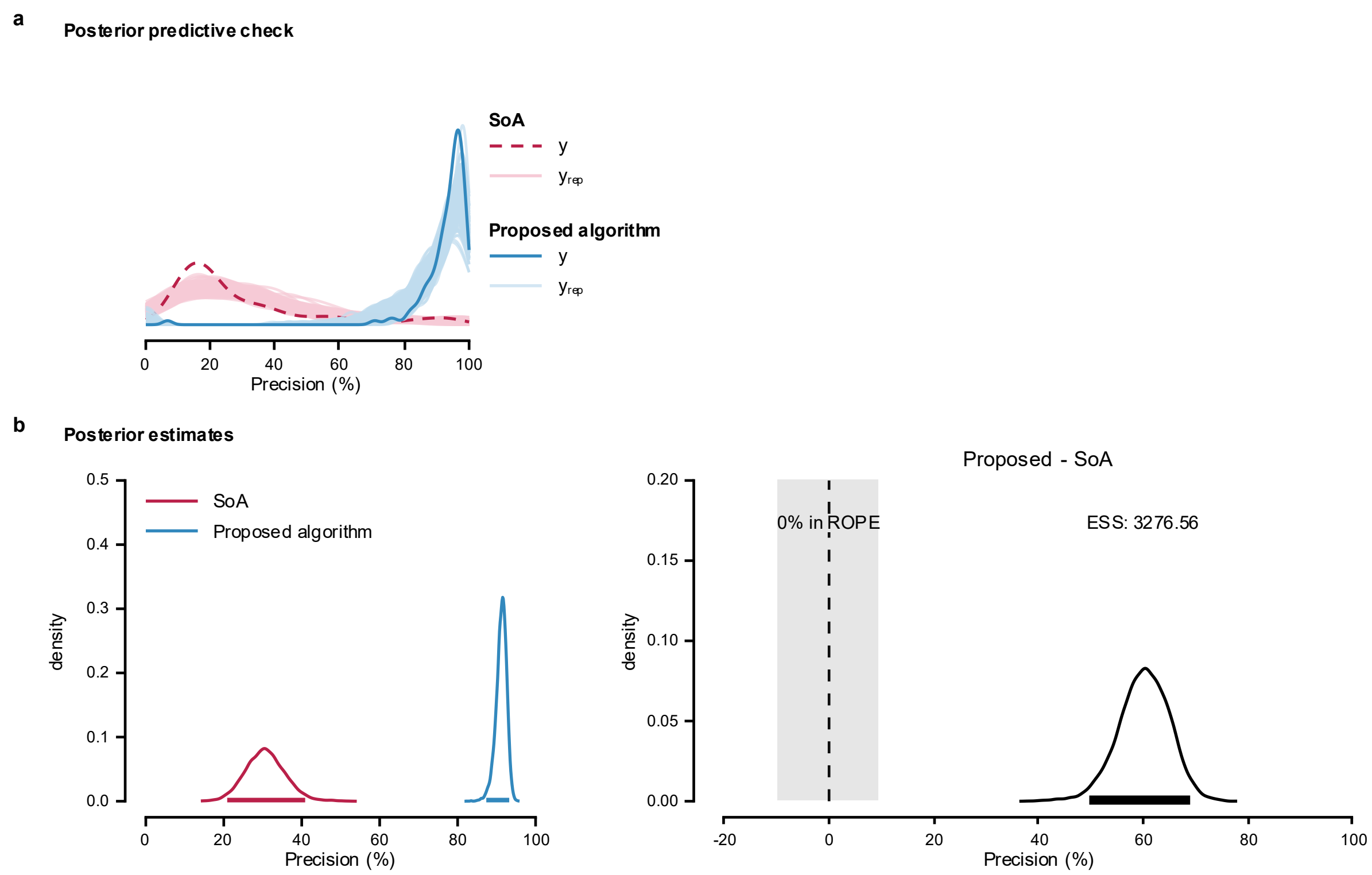

Supplementary Fig. 38: Posterior predictive check and posterior estimates for Model 3 of firing identification precision in tracked TA MUs across testing visits.

a, Posterior predictive check of the model; **b**, Posterior distributions and the difference between means estimated using Bayesian Model 1 to compare the performance of the proposed algorithm and SoA method.

MODEL COMPARISON

Model 2 showed the greatest expected predictive accuracy. ELPD diff relative to Model 2 were −1.3 (SE = 0.7) for Model 3 and −14.8 (SE = 5.2) for Model 1, suggesting similar predictive performance for Models 2 and 3 and reduced performance for Model 1.

Supplementary Note 15. Comparison of firing identification PNR for tracked MG MUs across testing visits.

Model implementation, likelihood function, and priors are detailed in Supplementary Note 5. The full dataset contains firing identification PNR values for 113 tracked MUs in MG muscle (Supplementary Fig. 39). The MUs firings were obtained by applying the MU filters calculated using the SoA algorithm[5] and the proposed algorithm to the HDsEMG signals obtained in the second testing visit.

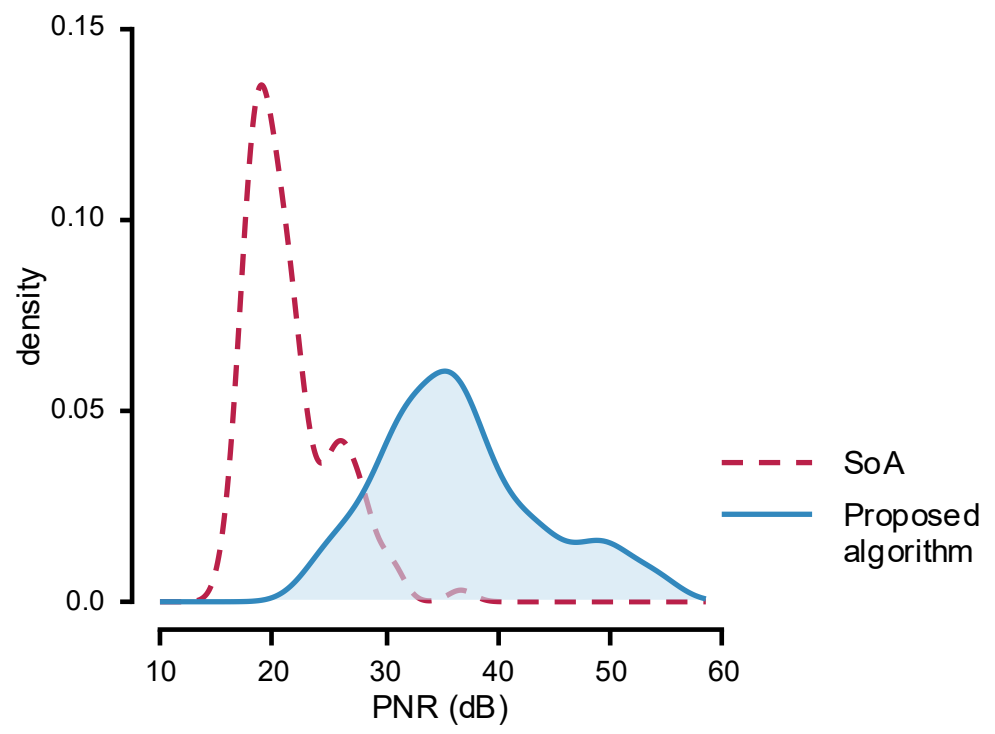

Supplementary Fig. 39: Distribution of firing identification PNR in tracked MG MUs across testing visits.

PNR values were calculated from firing identification obtained by applying MU filters calculated using SoA (dashed line) and the proposed algorithms (solid line) to the HDsEMG signals generated with synthetic grid displacement.

In all models, the lognormal mean and sigma effects were exponentiated before the posterior summaries were calculated. Group-level SDs are reported on their native model or link scales.

Model 1

The maximum PSRF across all parameters was 1.001, indicating good convergence, while the minimum ESS was 5,873.055, indicating stable estimates of the bounds of the credible intervals (Supplementary Table 55). Supplementary Table 56 provides median of the estimate value in the original scale of PNR, the limits of the 95% ETI, and the limits of the 95% HDI.

Supplementary Table 55: Posterior MCMC diagnostics for Model 1 of firing identification PNR in tracked MG MUs across testing visits.

	PSRF Pt	PSRF UpCI	ESS
α_{μ}	1.000	1.000	6,332.681
α_{σ}	1.000	1.000	1,6503.502
$\beta_{\mu,\text{Proposed}}$	1.000	1.000	2,4740.583
$\beta_{\sigma,\text{Proposed}}$	1.000	1.000	1,6925.304
τ_{μ}	1.001	1.001	5,873.055

Supplementary Table 56: Posterior summaries for Model 1 of firing identification PNR in tracked MG MUs across testing visits.

	50%	95% ETI		Mode	95% HDI	
		2.5%	97.5%		low	high
α_{μ}	21.612	20.207	23.165	21.619	20.200	23.155
α_{σ}	1.701	1.629	1.779	1.699	1.626	1.775
$\beta_{\mu,\text{Proposed}}$	0.155	0.134	0.181	0.154	0.133	0.180
$\beta_{\sigma,\text{Proposed}}$	1.173	0.930	1.470	1.164	0.919	1.455
τ_{μ}	0.086	0.047	0.167	0.077	0.043	0.156

Posterior predictive checks were performed by overlaying model-generated predictions on the observed data (Supplementary Fig. 40a). The posterior distributions (Supplementary Fig. 40b) indicated that the proposed algorithm achieved a higher PNR (37.4 dB, [34.9-40.1 dB]) compared to the SoA algorithm (21.9 dB, [20.4-23.4 dB]). The posterior distribution of the difference excluded zero, and less than 2.5% of its distribution fell within ROPE.

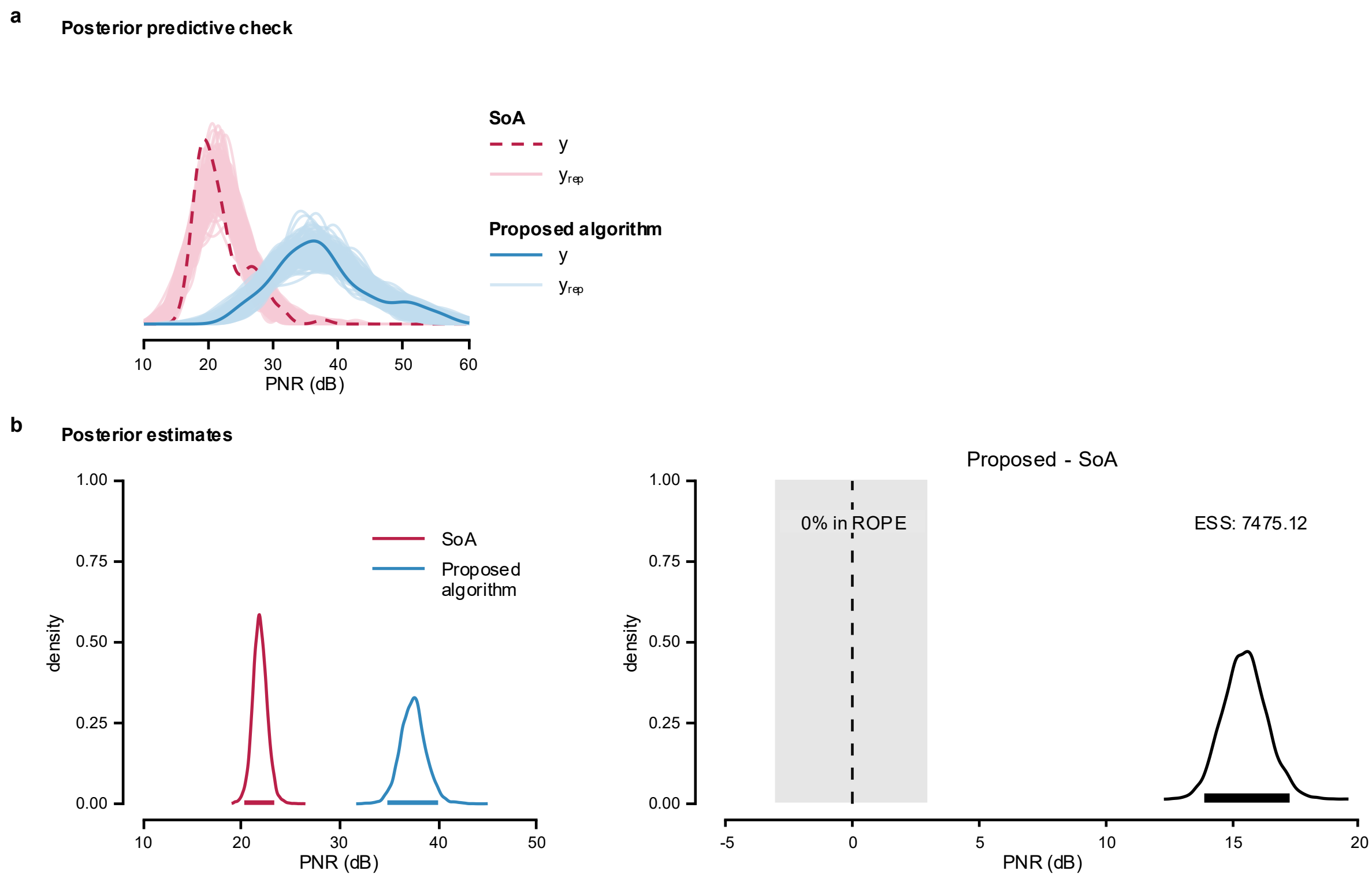

Supplementary Fig. 40: Posterior predictive check and posterior estimates for Model 1 of firing identification PNR in tracked MG MUs across testing visits.

a, Posterior predictive check of the model; **b**, Posterior distributions and the difference between means estimated using Bayesian Model 1 to compare the performance of the proposed algorithm and SoA method.

Model 2

The maximum PSRF across all parameters was 1.002, indicating good convergence, while the minimum ESS was 5,659.873, indicating stable estimates of the bounds of the credible intervals (Supplementary Table 57). Posterior parameter summaries are given as medians with 95% ETIs and 95% HDIs (Supplementary Table 58). Correlations were summarized on their native scales.

Posterior predictive checks were performed by overlaying model-generated predictions on the observed data (Supplementary Fig. 41a). The posterior distributions (Supplementary Fig. 41b) indicated that the proposed algorithm achieved a higher PNR (35.3 dB, [30.6-39.6 dB]) than the SoA algorithm (22.6 dB, [20.7-25.0 dB]). The posterior distribution of the difference excluded zero, and less than 2.5% of its distribution fell within ROPE.

Model 3

The maximum PSRF across all parameters was 1.004, indicating good convergence, while the minimum ESS was 2,644.540, indicating stable estimates of the bounds of the credible intervals (Supplementary Table 59). Posterior parameter summaries are given as medians with 95% ETIs and 95% HDIs (Supplementary Table 60). Correlations were summarized on their native scales.

Posterior predictive checks were performed by overlaying model-generated predictions on the observed data (Supplementary Fig. 42a). The posterior distributions (Supplementary Fig. 42b) indicated that the proposed algorithm achieved a higher PNR (35.3 dB, [30.4-39.6 dB]) than the SoA algorithm (22.6 dB, [20.7-24.9 dB]). The posterior distribution of the difference excluded zero, and less than 2.5% of its distribution fell within ROPE.

Supplementary Table 57: Posterior MCMC diagnostics for Model 2 of firing identification PNR in tracked MG MUs across testing visits.

	PSRF Pt	PSRF UpCI	ESS
α_{μ}	1.001	1.002	5,805.282
α_{σ}	1.000	1.000	1,9194.010
$\beta_{\mu,\text{Proposed}}$	1.001	1.002	5,865.638
$\beta_{\sigma,\text{Proposed}}$	1.000	1.000	2,1977.249
τ_{b0}	1.000	1.000	5,828.215
$\tau_{b\text{Proposed}}$	1.001	1.002	5,659.873
R_{12}	1.000	1.000	7,316.349

Supplementary Table 58: Posterior summaries for Model 2 of firing identification PNR in tracked MG MUs across testing visits.

	50%	95% ETI		Mode	95% HDI	
		2.5%	97.5%		low	high
α_{μ}	22.302	20.478	24.764	22.262	20.339	24.574
α_{σ}	0.134	0.118	0.154	0.132	0.117	0.153
$\beta_{\mu,\text{Proposed}}$	1.568	1.318	1.780	1.573	1.332	1.791
$\beta_{\sigma,\text{Proposed}}$	1.130	0.943	1.356	1.127	0.932	1.340
τ_{b0}	0.127	0.077	0.232	0.118	0.068	0.213
$\tau_{b\text{Proposed}}$	0.206	0.127	0.378	0.182	0.116	0.347
R_{12}	-0.558	-0.873	0.071	-0.653	-0.918	-0.030

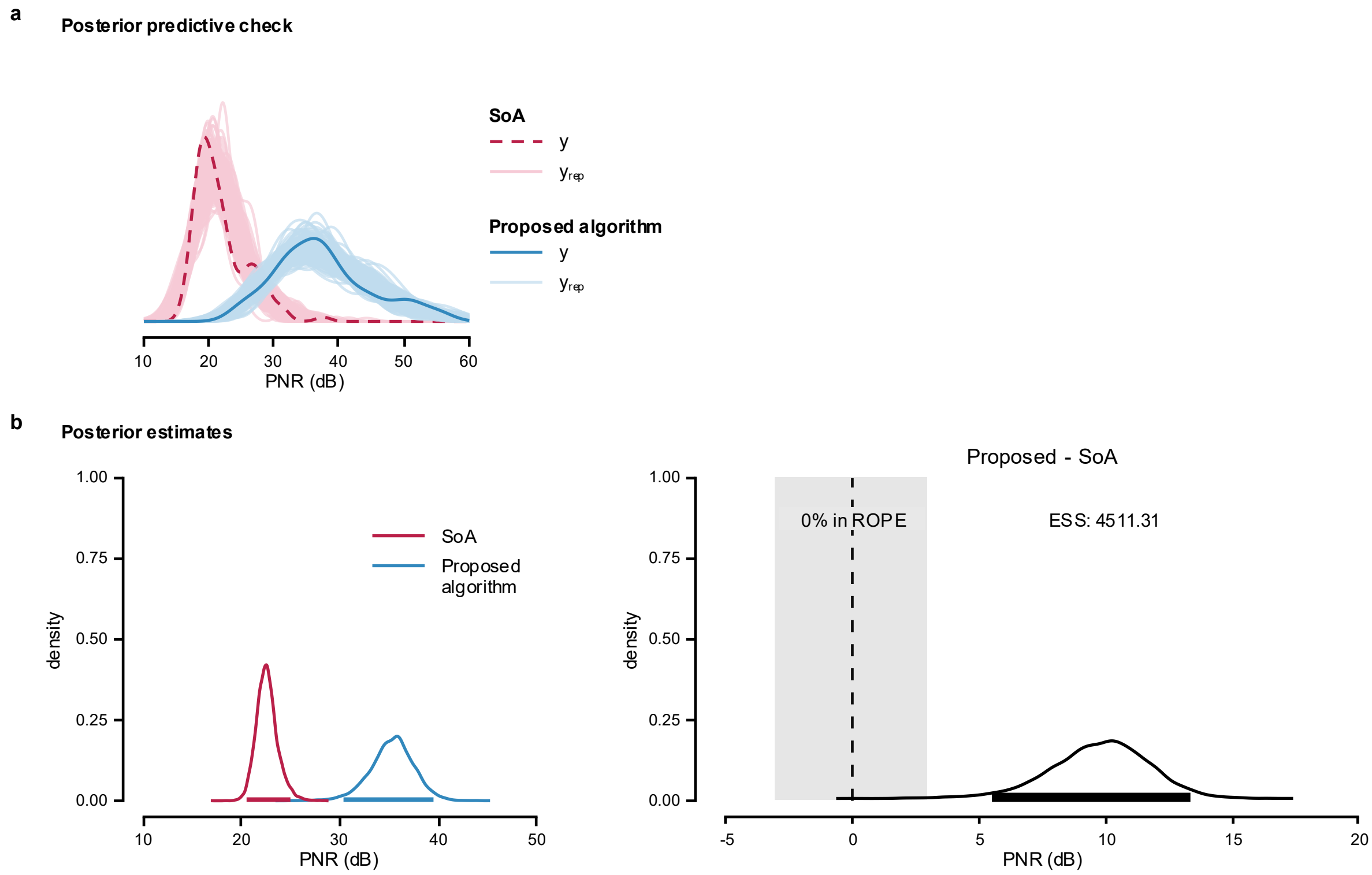

Supplementary Fig. 41: Posterior predictive check and posterior estimates for Model 2 of firing identification PNR in tracked MG muscle MUs across testing visits.

a, Posterior predictive check of the model; **b**, Posterior distributions and the difference in means from Bayesian Model 2, used to compare the performance of the proposed algorithm and SoA method.

Supplementary Table 59: Posterior MCMC diagnostics for Model 3 of firing identification PNR in tracked MG MUs across testing visits.

	PSRF Pt	PSRF UpCI	ESS
α_{μ}	1.000	1.001	4,358.505
α_{σ}	1.000	1.001	6,124.032
$\beta_{\mu,\text{Proposed}}$	1.000	1.001	4,564.199
$\beta_{\sigma,\text{Proposed}}$	1.000	1.000	1,8511.083
τ_{b0}	1.001	1.003	6,140.874
$\tau_{b\text{Proposed}}$	1.000	1.000	4,941.607
τ_{c}	1.001	1.004	2,644.540
R_{12}	1.000	1.001	5,771.900

Supplementary Table 60: Posterior summaries for Model 3 of firing identification PNR in tracked MG MUs across testing visits.

	50%	95% ETI		Mode	95% HDI	
		2.5%	97.5%		low	high
α_{μ}	22.312	20.480	24.718	22.169	20.257	24.455
α_{σ}	0.127	0.106	0.148	0.128	0.106	0.148
$\beta_{\mu,\text{Proposed}}$	1.567	1.319	1.775	1.587	1.323	1.778
$\beta_{\sigma,\text{Proposed}}$	1.142	0.936	1.403	1.110	0.926	1.390
τ_{b0}	0.127	0.076	0.226	0.114	0.069	0.211
$\tau_{b\text{Proposed}}$	0.208	0.129	0.377	0.186	0.114	0.344
τ_{c}	0.043	0.003	0.078	0.050	0.000	0.073
R_{12}	-0.562	-0.875	0.053	-0.662	-0.910	-0.034

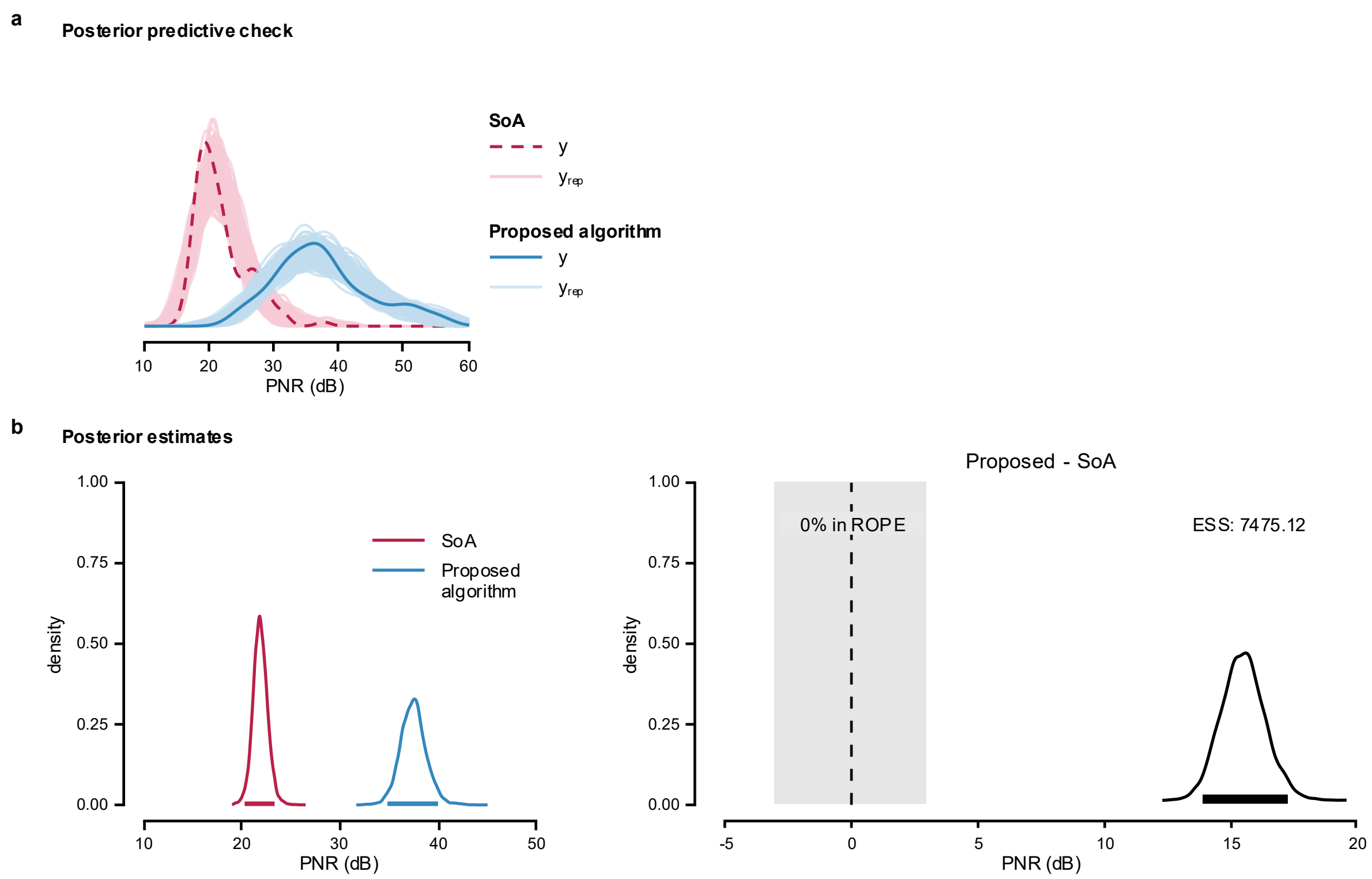

Supplementary Fig. 42: Posterior predictive check and posterior estimates for Model 3 of firing identification PNR in tracked MG MUs across testing visits.

a, Posterior predictive check of the model; **b**, Posterior distributions and the difference in means from Bayesian Model 3, used to compare the performance of the proposed algorithm and SoA method.

MODEL COMPARISON

Model 2 showed the greatest expected predictive accuracy. ELPD diff relative to Model 2 were −0.01 (SE = 1.7) for Model 3 and −34.0 (SE = 7.6) for Model 1, suggesting similar predictive performance for Models 2 and 3 and reduced performance for Model 1.

Supplementary Note 16. Comparison of firing identification sensitivity in tracked MG MUs across testing visits.

Model implementation, likelihood function, and priors are detailed in Supplementary Note 5. The full dataset contains firing identification sensitivity values for 113 tracked MUs (hereafter referred to as 'sensitivity') in MG muscle. The MUs firings were obtained by applying the MU filters calculated using the SoA algorithm[5] and the proposed algorithm to the HDsEMG signals obtained in the second testing visit. Distribution of the data is shown in Supplementary Fig. 43.

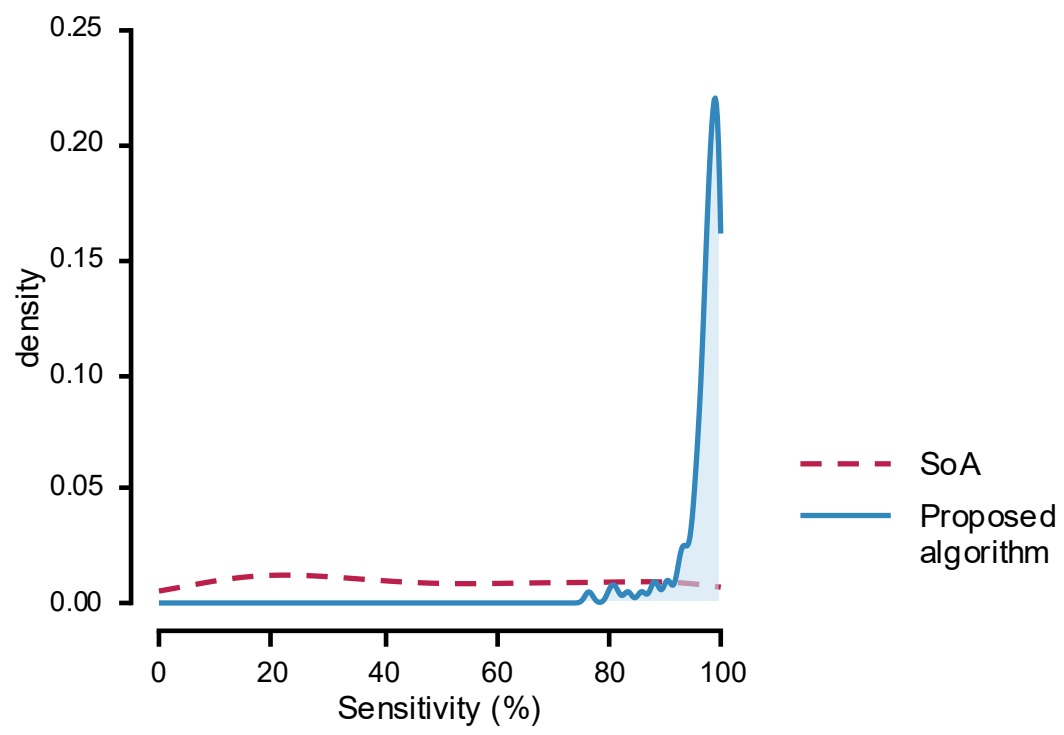

Supplementary Fig. 43: Distribution of firing identification sensitivity in tracked MG MUs across testing visits.

Sensitivity values were calculated from firing identification obtained by applying MU filters calculated using SoA (dashed line) and the proposed algorithms (solid line) to the HDsEMG signals generated with synthetic grid displacement.

In all models, ZOIB parameters were back transformed into their interpretable component scales: *mu*, *zoi*, and *coi* to probabilities, and *phi* to the positive precision scale. Group-level SDs are reported on their native model/link scale.

Model 1

The maximum PSRF across all parameters was 1.004, indicating good convergence, while the minimum ESS was 4,839.106, indicating stable estimates of the bounds of the credible intervals (Supplementary Table 61). Posterior summaries are reported in Supplementary Table 62 as medians with 95% ETIs and 95% HDIs. Group-level correlations are reported on the correlation scale.

Posterior predictive checks were performed by overlaying model-generated predictions on the observed data (Supplementary Fig. 44a). The posterior distributions (Supplementary Fig. 44a) indicated that the proposed algorithm achieved a higher sensitivity (96.1%, [93.8-97.5 %]) than the SoA algorithm (51.4%, [43.3-59.0%]). The posterior distribution of the difference excluded zero, and less than 2.5% of its distribution fell within ROPE.

Supplementary Table 61: Posterior MCMC diagnostics for Model 1 of firing identification sensitivity in tracked MG MUs across testing visits.

	PSRF Pt	PSRF UpCI	ESS
α_{μ}	1.000	1.000	7,797.901
α_{ϕ}	1.000	1.000	1,2026.624
α_{ζ}	1.001	1.002	1,0378.099
α_{κ}	1.000	1.000	1,3885.065
$\beta_{\mu,\text{Proposed}}$	1.000	1.000	1,6491.057
$\beta_{\phi,\text{Proposed}}$	1.000	1.000	1,3513.627
$\beta_{\zeta,\text{Proposed}}$	1.001	1.002	1,1425.987
$\beta_{\kappa,\text{Proposed}}$	1.000	1.000	1,5895.304
τ_{b}	1.001	1.004	4,839.106

Supplementary Table 62: Posterior summaries for Model 1 of firing identification sensitivity in tracked MG MUs across testing visits.

	**50%**	**95% ETI**		**Mode**	**95% HDI**	
		**2.5%**	**97.5%**		**low**	**high**
α_{μ}	0.518	0.435	0.594	0.520	0.435	0.594
α_{ϕ}	2.257	1.725	2.935	2.236	1.711	2.909
α_{ζ}	0.014	0.002	0.043	0.009	0.001	0.037
α_{κ}	0.233	0.011	0.898	0.055	0.001	0.841
$\beta_{\mu,\text{Proposed}}$	26.266	19.897	34.704	25.728	19.554	34.233
$\beta_{\phi,\text{Proposed}}$	13.146	8.484	19.958	12.835	8.106	19.283
$\beta_{\zeta,\text{Proposed}}$	12.022	3.101	72.367	6.865	1.274	50.916
$\beta_{\kappa,\text{Proposed}}$	67.964	1.125	5,593.709	-75.612	0.015	2,599.469
τ_{b}	0.335	0.070	0.753	0.302	0.009	0.665

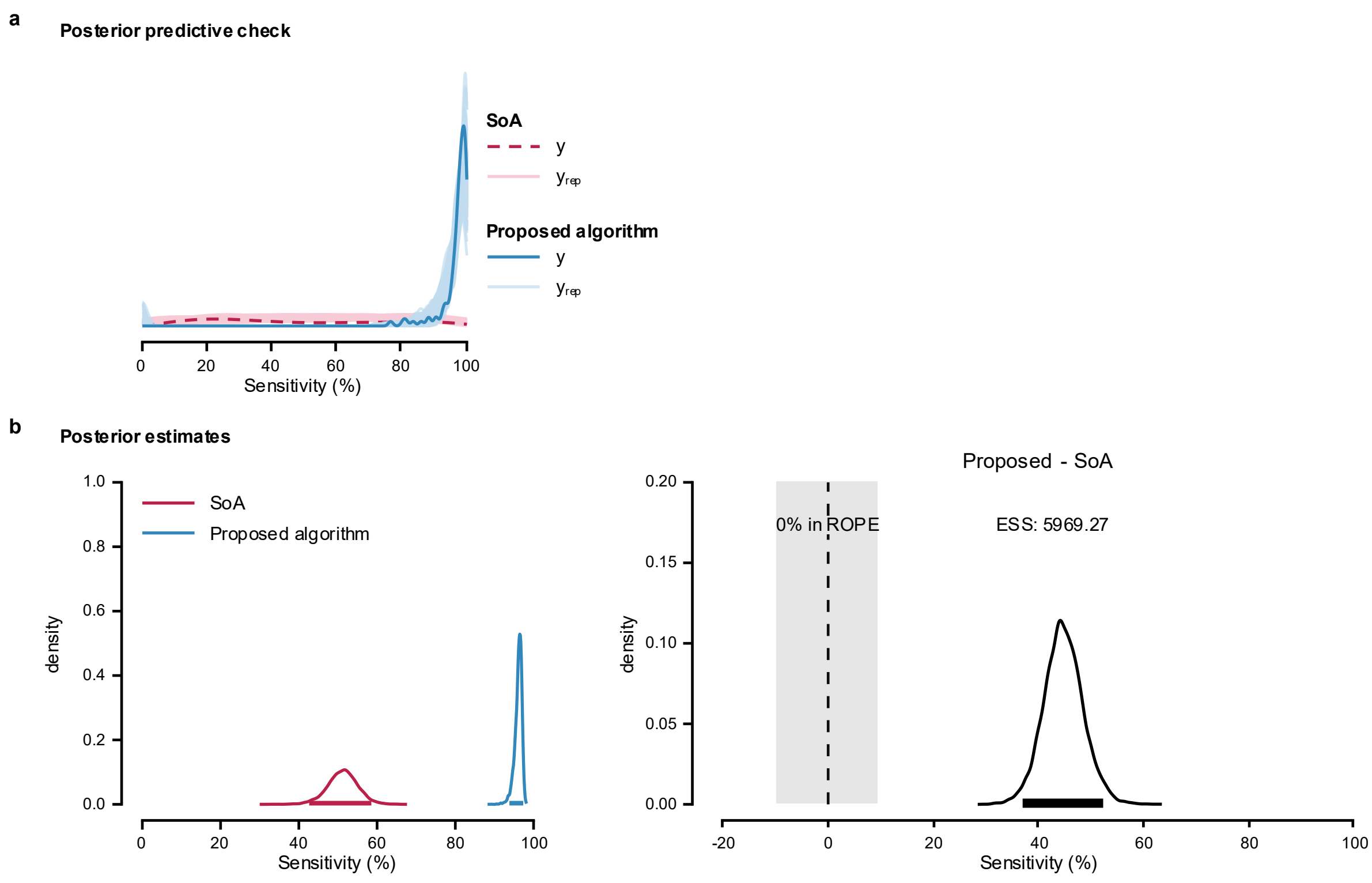

Supplementary Fig. 44: Posterior predictive check and posterior estimates for Model 1 of firing identification sensitivity in tracked MG MUs across testing visits.

a, Posterior predictive check of the model; **b**, Posterior distributions and the difference between means estimated using Bayesian Model 1 to compare the performance of the proposed algorithm and SoA method.

<u>Model 2</u>

The maximum PSRF across all parameters was 1.003, indicating good convergence, while the minimum ESS was 6,395.834, indicating stable estimates of the bounds of the credible intervals (Supplementary Table 63). Posterior summaries are reported in Supplementary Table 64 as medians with 95% ETIs and 95% HDIs. Group-level correlations are reported on the correlation scale.

Posterior predictive checks were performed by overlaying model-generated predictions on the observed data (Supplementary Fig. 45a). The posterior distributions (Supplementary Fig.

45b) indicated that the proposed algorithm achieved a higher sensitivity (96.0%, [93.6-97.3%]) than the SoA algorithm (52.9%, [39.8-65.1%]). The posterior distribution of the difference excluded zero, and less than 2.5% of its distribution fell within ROPE.

Supplementary Table 63: Posterior MCMC diagnostics for Model 2 of firing identification sensitivity in tracked MG MUs across testing visits.

	PSRF Pt	PSRF UpCI	ESS
α_{μ}	1.001	1.001	6,395.834
α_{ϕ}	1.000	1.000	2,0443.992
α_{ζ}	1.000	1.000	1,4026.245
α_{κ}	1.000	1.000	1,8505.763
$\beta_{\mu,\text{Proposed}}$	1.000	1.000	7,832.789
$\beta_{\phi,\text{Proposed}}$	1.000	1.000	2,1415.251
$\beta_{\zeta,\text{Proposed}}$	1.000	1.000	1,4427.101
$\beta_{\kappa,\text{Proposed}}$	1.000	1.001	1,9589.266
τ_{b0}	1.001	1.002	6,746.835
$\tau_{b\text{Proposed}}$	1.001	1.003	7,614.119
R_{12}	1.001	1.002	9,532.917

1615 **Supplementary Table 64: Posterior summaries for Model 2 of firing identification**

1616 **sensitivity in tracked MG MUs across testing visits.**

	50%	95% ETI		Mode	95% HDI	
		2.5%	97.5%		low	high
α_{μ}	0.535	0.402	0.659	0.543	0.404	0.661
α_{ϕ}	3.012	2.293	3.878	2.977	2.249	3.818
α_{ζ}	0.014	0.003	0.044	0.009	0.001	0.037
α_{κ}	0.235	0.012	0.896	0.054	0.001	0.838
$\beta_{\mu,\text{Proposed}}$	23.360	12.961	40.580	22.169	11.791	38.420
$\beta_{\phi,\text{Proposed}}$	10.358	6.865	15.461	9.984	6.577	14.964
$\beta_{\zeta,\text{Proposed}}$	11.943	3.150	69.443	7.100	1.521	51.155
$\beta_{\kappa,\text{Proposed}}$	67.527	1.132	5,103.709	-76.205	0.012	2,531.192
τ_{b0}	0.729	0.433	1.233	0.670	0.403	1.178
$\tau_{b\text{Proposed}}$	0.747	0.408	1.299	0.694	0.380	1.242
R_{12}	-0.879	-0.986	-0.414	-0.948	-0.998	-0.524

1617

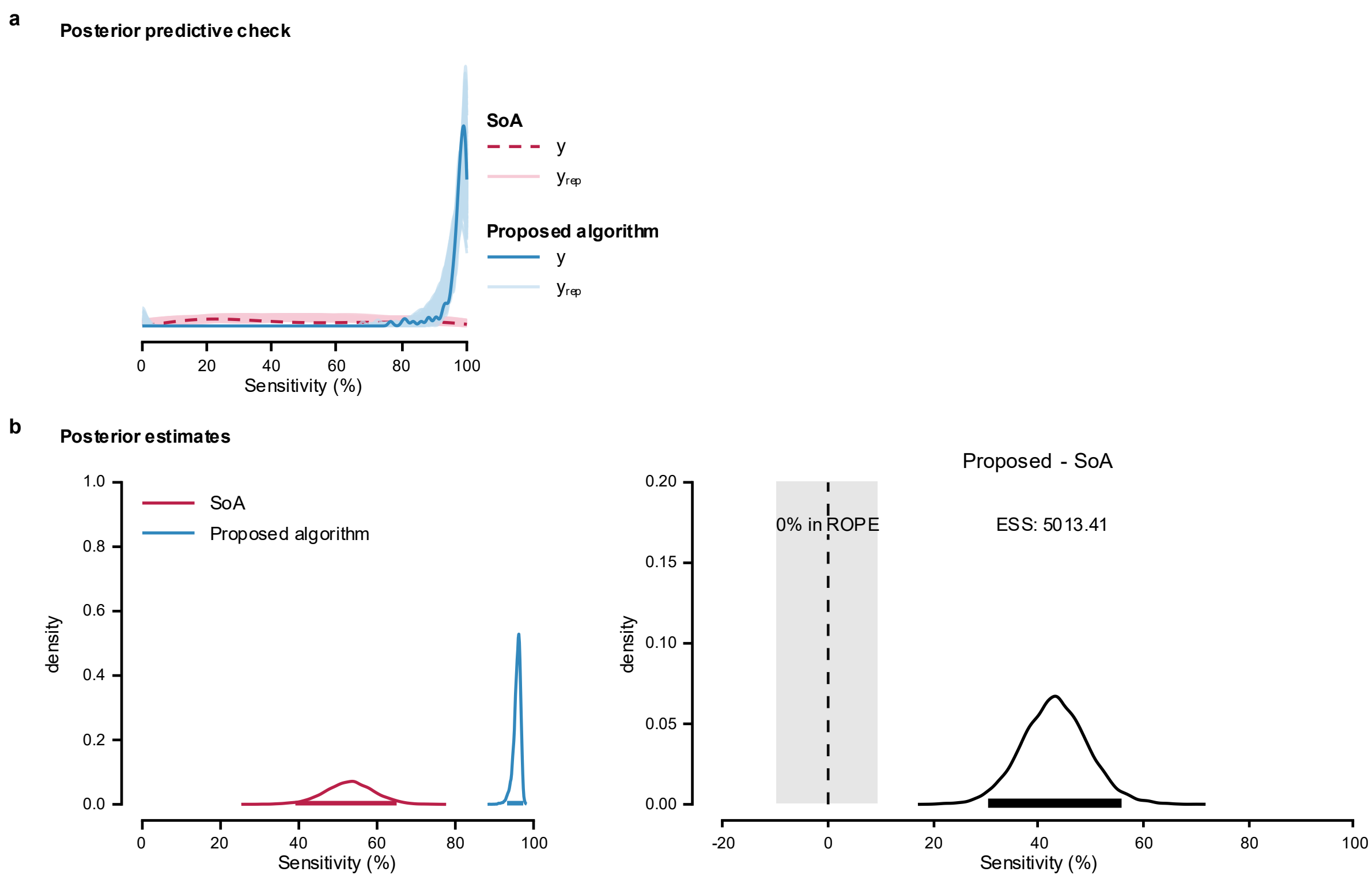

Supplementary Fig. 45: Posterior predictive check and posterior estimates for Model 2 of firing identification sensitivity in tracked MG MUs across testing visits.

a, Posterior predictive check of the model; **b**, Posterior distributions and the difference in means from Bayesian Model 2, used to compare the performance of the proposed algorithm and SoA method.

<u>Model 3</u>

The maximum PSRF across all parameters was 1.006, indicating good convergence, while the minimum ESS was 719.030, indicating stable estimates of the bounds of the credible intervals (Supplementary Table 65). Posterior summaries are reported as medians with 95% ETIs and 95% HDIs (Supplementary Table 66). Group-level correlations are reported on the correlation scale.

Posterior predictive checks were performed by overlaying model-generated predictions on the observed data (Supplementary Fig. 46a). The posterior distributions (Supplementary Fig.

46b) indicated that the proposed algorithm achieved a higher sensitivity (96.1%, [93.7-97.6%]) than the SoA algorithm (52.7%, [38.9-65.8%]). The posterior distribution of the difference excluded zero, and less than 2.5% of its distribution fell within ROPE.

Supplementary Table 65: Posterior MCMC diagnostics for Model 3 of firing identification sensitivity in tracked MG MUs across testing visits.

	PSRF Pt	PSRF UpCI	ESS
α_{μ}	1.000	1.001	5,036.184
α_{ϕ}	1.000	1.001	2,728.908
α_{ζ}	1.000	1.001	1,4121.714
α_{κ}	1.000	1.000	2,1999.444
$\beta_{\mu,\text{Proposed}}$	1.000	1.001	5,364.949
$\beta_{\phi,\text{Proposed}}$	1.003	1.006	719.030
$\beta_{\zeta,\text{Proposed}}$	1.000	1.001	1,4775.563
$\beta_{\kappa,\text{Proposed}}$	1.000	1.000	2,2125.638
τ_{b0}	1.000	1.000	5,943.786
$\tau_{b\text{Proposed}}$	1.000	1.000	6,944.338
τ_{c}	1.002	1.005	748.043
R_{12}	1.001	1.002	9,714.394

Supplementary Table 66: Posterior summaries for Model 3 of firing identification sensitivity in tracked MG MUs across testing visits.

	50%	95% ETI		Mode	95% HDI	
		2.5%	97.5%		low	high
α_{μ}	0.532	0.393	0.660	0.534	0.394	0.661
α_{ϕ}	3.266	2.417	4.705	3.118	2.294	4.514
α_{ζ}	0.014	0.003	0.044	0.010	0.001	0.037
α_{κ}	0.238	0.011	0.897	0.054	0.001	0.839
$\beta_{\mu,\text{Proposed}}$	24.620	13.294	46.154	23.050	11.575	42.878
$\beta_{\phi,\text{Proposed}}$	11.111	7.067	34.400	10.098	5.757	25.570
$\beta_{\zeta,\text{Proposed}}$	11.844	3.212	65.983	6.545	1.439	48.714
$\beta_{\kappa,\text{Proposed}}$	67.131	1.159	5,480.474	-74.184	0.045	2,587.436
τ_{b0}	0.755	0.447	1.293	0.693	0.416	1.231
$\tau_{b\text{Proposed}}$	0.788	0.430	1.362	0.766	0.382	1.284
τ_{c}	0.217	0.009	0.761	0.052	0.000	0.697
R_{12}	-0.869	-0.984	-0.393	-0.942	-0.998	-0.500

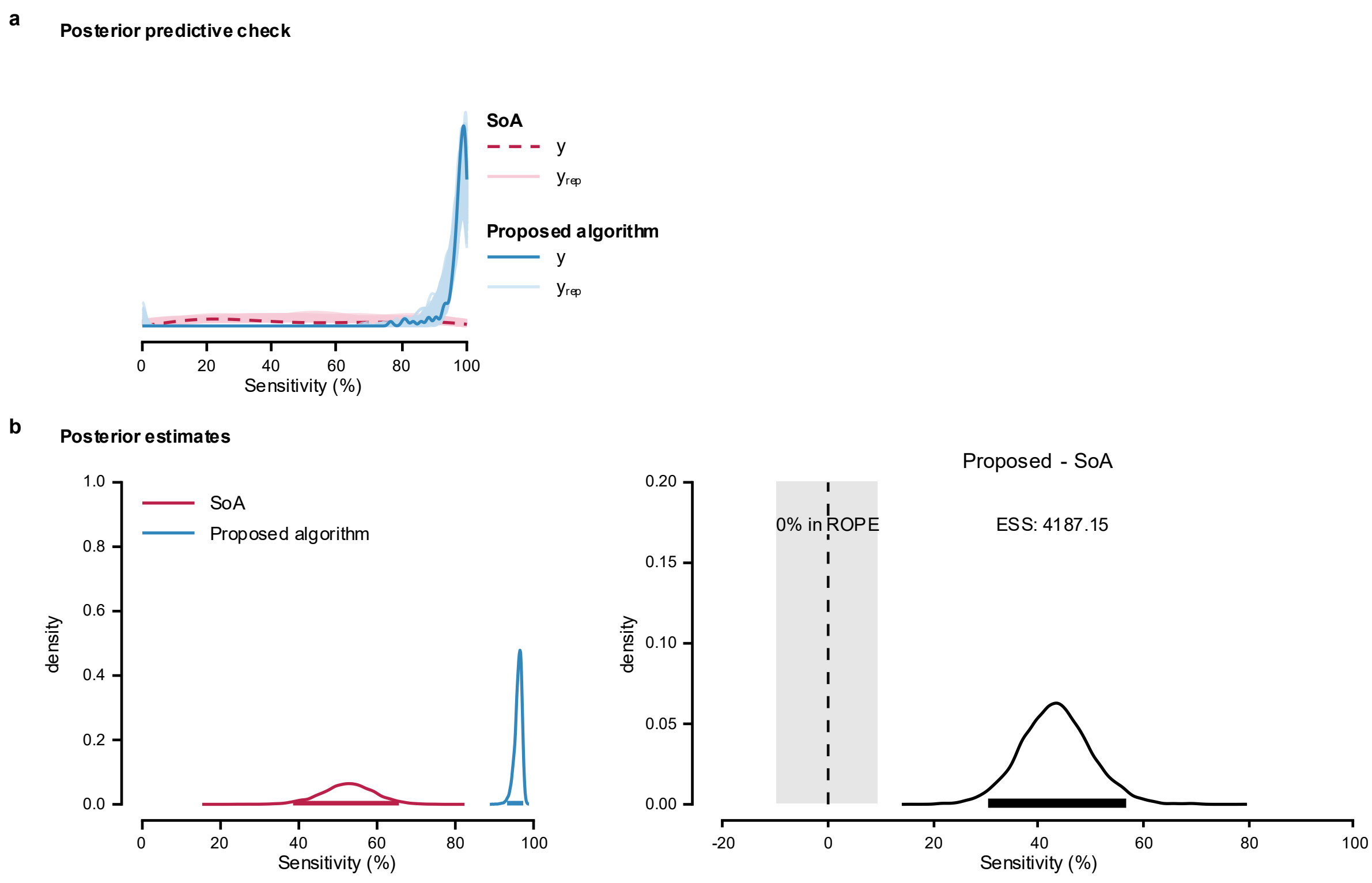

Supplementary Fig. 46: Posterior predictive check and posterior estimates for Model 3 of firing identification sensitivity in tracked MG MUs across testing visits.

a, Posterior predictive check of the model; **b**, Posterior distributions and the difference in means from Bayesian Model 3, used to compare the performance of the proposed algorithm and SoA method.

MODEL COMPARISON

Model 2 showed the greatest expected predictive accuracy. ELPD diff relative to Model 2 were −1.9 (SE = 2.5) for Model 3 and −15.4 (SE = 4.7) for Model 1, suggesting similar predictive performance for Models 2 and 3 and reduced performance for Model 1.

Supplementary Note 17. Comparison of firing identification precision in tracked MG MUs across testing visits.

Model implementation, likelihood function, and priors are detailed in Supplementary Note 5. The full dataset contains firing identification precision values for 113 tracked MG MUs (hereafter referred to as 'precision'). The MUs firings were obtained by applying the MU filters calculated using the SoA algorithm[5] and the proposed algorithm to the HDsEMG signals obtained in the second testing visit. Distribution of the data is shown in Supplementary Fig. 47.

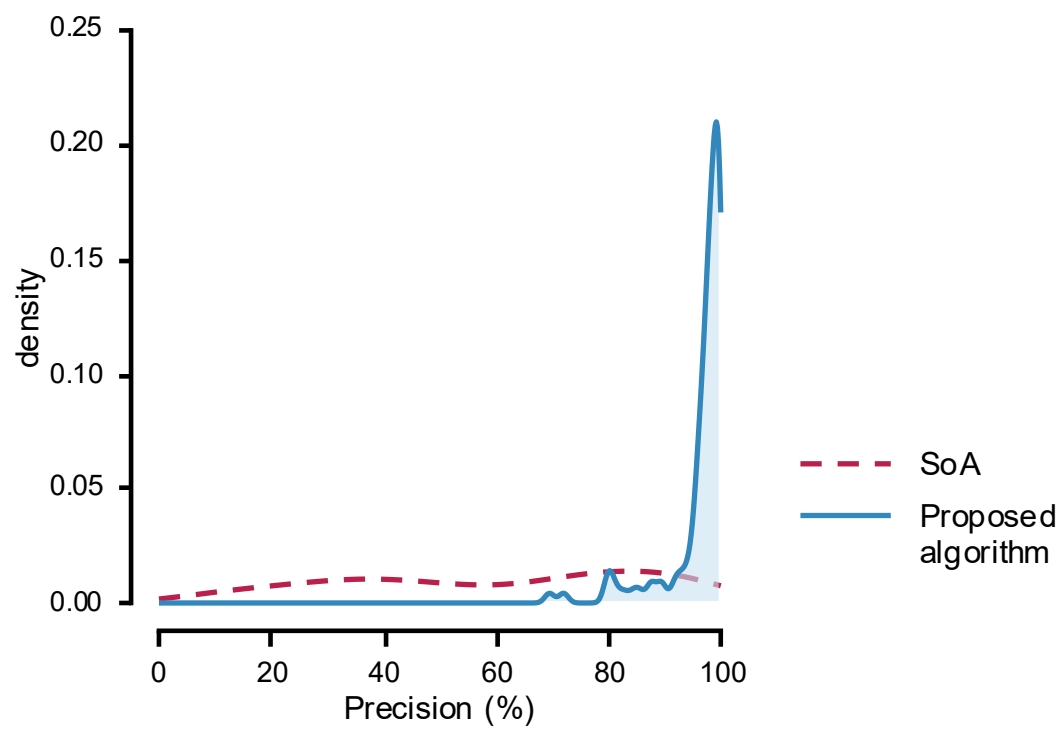

Supplementary Fig. 47: Distribution of firing identification precision in tracked MG MUs across testing visits.

Precision values were calculated from firing identification obtained by applying MU filters calculated using SoA (dashed line) and the proposed algorithms (solid line) to the HDsEMG signals generated with synthetic grid displacement.

In all models, ZOIB parameters were back transformed into their interpretable component scales: *mu*, *zoi*, and *coi* to probabilities, and *phi* to the positive precision scale. Group-level SDs are reported on their native model/link scale.

Model 1

The maximum PSRF across all parameters was 1.002, indicating good convergence, while the minimum ESS was 6,125.286, indicating stable estimates of the bounds of the credible intervals (Supplementary Table 67). Posterior summaries are reported in Supplementary Table 68 as medians with 95% ETIs and 95% HDIs. Group-level correlations are reported on the correlation scale.

Posterior predictive checks were performed by overlaying model-generated predictions on the observed data (Supplementary Fig. 48a). The posterior distributions (Supplementary Fig. 48b) indicated that the proposed algorithm achieved a higher precision (95.2%, [92.8-96.8 %]) than the SoA algorithm (57.9%, [50.4-64.7%]). The posterior distribution of the difference excluded zero, and less than 2.5% of its distribution fell within ROPE.

Supplementary Table 67: Posterior MCMC diagnostics for Model 1 of firing identification precision in tracked MG MUs across testing visits.

	PSRF Pt	PSRF UpCI	ESS
α_{μ}	1.000	1.001	1,0126.198
α_{ϕ}	1.000	1.001	2,1207.749
α_{ζ}	1.000	1.000	1,5749.186
α_{κ}	1.000	1.000	2,4702.617
$\beta_{\mu,\text{Proposed}}$	1	1.001	2,2631.534
$\beta_{\phi,\text{Proposed}}$	1.000	1.001	2,0799.107
$\beta_{\zeta,\text{Proposed}}$	1.000	1.000	1,6660.903
$\beta_{\kappa,\text{Proposed}}$	1.000	1.000	2,5294.913
τ_b	1.001	1.002	5,537.584

Supplementary Table 68: Posterior summaries for Model 1 of firing identification precision in tracked MG MUs across testing visits.

	50%	95% ETI		Mode	95% HDI	
		2.5%	97.5%		low	high
α_{μ}	0.581	0.505	0.649	0.585	0.508	0.651
α_{ϕ}	2.789	2.158	3.555	2.747	2.119	3.501
α_{ζ}	0.021	0.005	0.056	0.016	0.002	0.050
α_{κ}	0.537	0.070	0.950	0.613	0.081	0.958
$\beta_{\mu,\text{Proposed}}$	15.539	11.579	20.600	15.279	11.388	20.304
$\beta_{\phi,\text{Proposed}}$	6.868	4.512	10.240	6.595	4.380	10.013
$\beta_{\zeta,\text{Proposed}}$	9.389	2.984	42.956	6.121	1.544	32.650
$\beta_{\kappa,\text{Proposed}}$	17.654	0.580	700.510	-14.805	0.018	351.051
τ_b	0.308	0.082	0.673	0.278	0.047	0.621

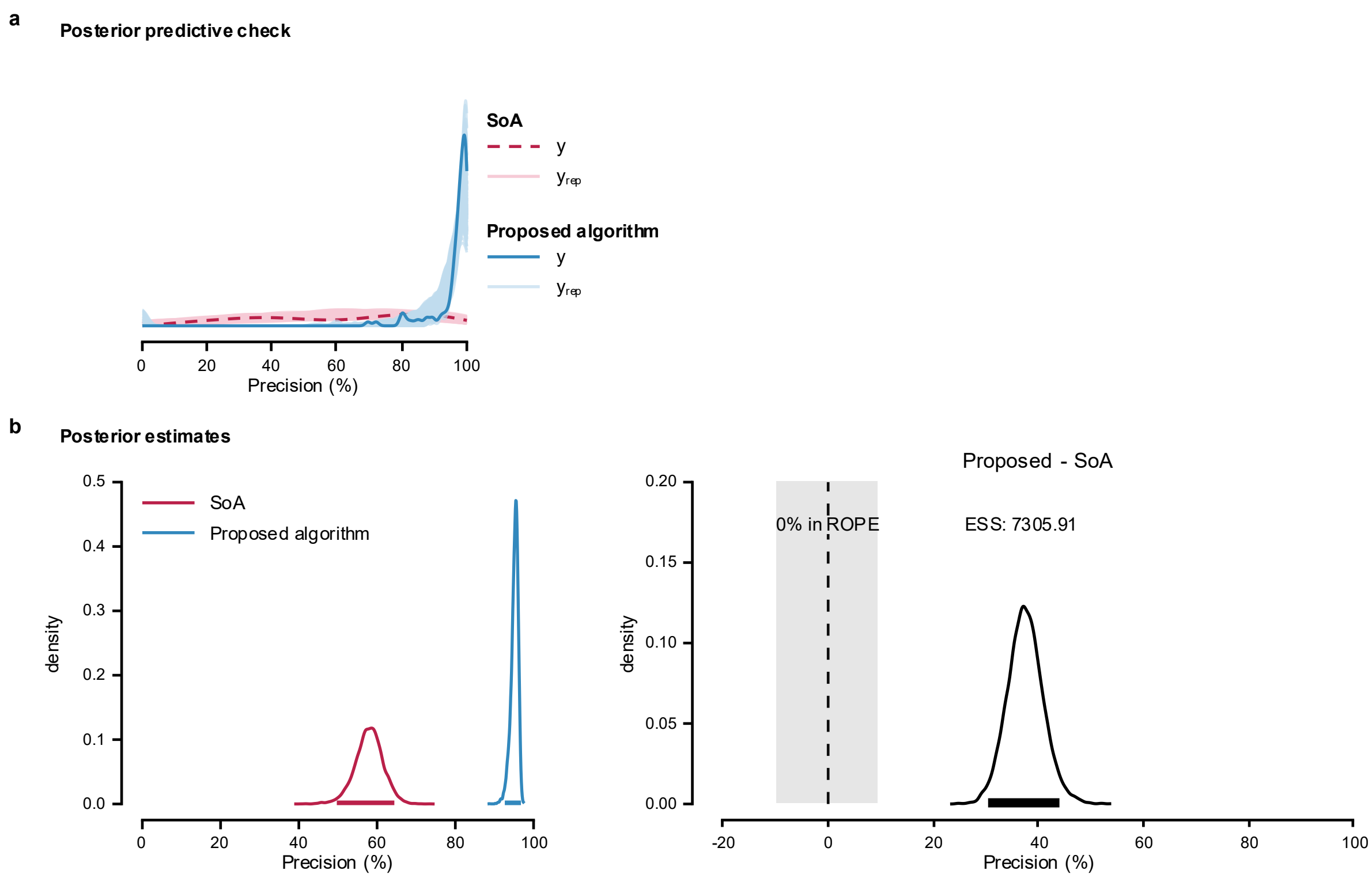

Supplementary Fig. 48: Posterior predictive check and posterior estimates for Model 1 of firing identification precision in tracked MG MUs across testing visits.

a, Posterior predictive check of the model; **b**, Posterior distributions and the difference between means estimated using Bayesian Model 1 to compare the performance of the proposed algorithm and SoA method.

Model 2

The maximum PSRF across all parameters was 1.002, indicating good convergence, while the minimum ESS was 6,162.999, indicating stable estimates of the bounds of the credible intervals (Supplementary Table 51). Posterior summaries are reported as medians with 95% ETIs and 95% HDIs (Supplementary Table 70). Group-level correlations are reported on the correlation scale.

Posterior predictive checks were performed by overlaying model-generated predictions on the observed data (Supplementary Fig. 49a). The posterior distributions (Supplementary Fig.

49b) indicated that the proposed algorithm achieved a higher precision (95.1%, [92.6-96.8%]) than the SoA algorithm (58.3%, [49.5-66.9%]). The posterior distribution of the difference excluded zero, and less than 2.5% of its distribution fell within ROPE.

Supplementary Table 69: Posterior MCMC diagnostics for Model 2 of firing identification precision in tracked MG MUs across testing visits.

	PSRF Pt	PSRF UpCI	ESS
α_{μ}	1.000	1.001	9,898.987
α_{ϕ}	1.001	1.001	1,5328.884
α_{ζ}	1.000	1.001	1,8374.223
α_{κ}	1.000	1.000	2,5391.269
$\beta_{\mu,\text{Proposed}}$	1.000	1.001	1,3683.989
$\beta_{\phi,\text{Proposed}}$	1.000	1.001	2,0644.275
$\beta_{\zeta,\text{Proposed}}$	1.000	1.001	1,9311.802
$\beta_{\kappa,\text{Proposed}}$	1.000	1.000	2,5785.492
τ_{b0}	1.001	1.002	6,538.517
$\tau_{b\text{Proposed}}$	1.001	1.002	6,162.999
R_{12}	1.000	1.001	1,0492.527

Supplementary Table 70: Posterior summaries for Model 2 of firing identification precision in tracked MG MUs across testing visits.

	50%	95% ETI		Mode	95% HDI	
		2.5%	97.5%		low	high
α_{μ}	0.585	0.494	0.671	0.582	0.494	0.670
α_{ϕ}	2.983	2.283	3.856	2.946	2.236	3.788
α_{ζ}	0.021	0.005	0.054	0.016	0.003	0.049
α_{κ}	0.536	0.069	0.948	0.645	0.090	0.966
$\beta_{\mu,\text{Proposed}}$	15.079	9.975	21.620	14.671	9.698	21.270
$\beta_{\phi,\text{Proposed}}$	6.488	4.250	9.735	6.421	4.033	9.392
$\beta_{\zeta,\text{Proposed}}$	9.530	3.022	41.290	6.192	1.469	31.235
$\beta_{\kappa,\text{Proposed}}$	17.023	0.569	707.389	-14.814	0.023	369.925
τ_{b0}	0.443	0.159	0.880	0.420	0.136	0.840
$\tau_{b\text{Proposed}}$	0.316	0.019	0.773	0.289	0.000	0.692
R_{12}	-0.601	-0.951	0.467	-0.825	-0.992	0.288

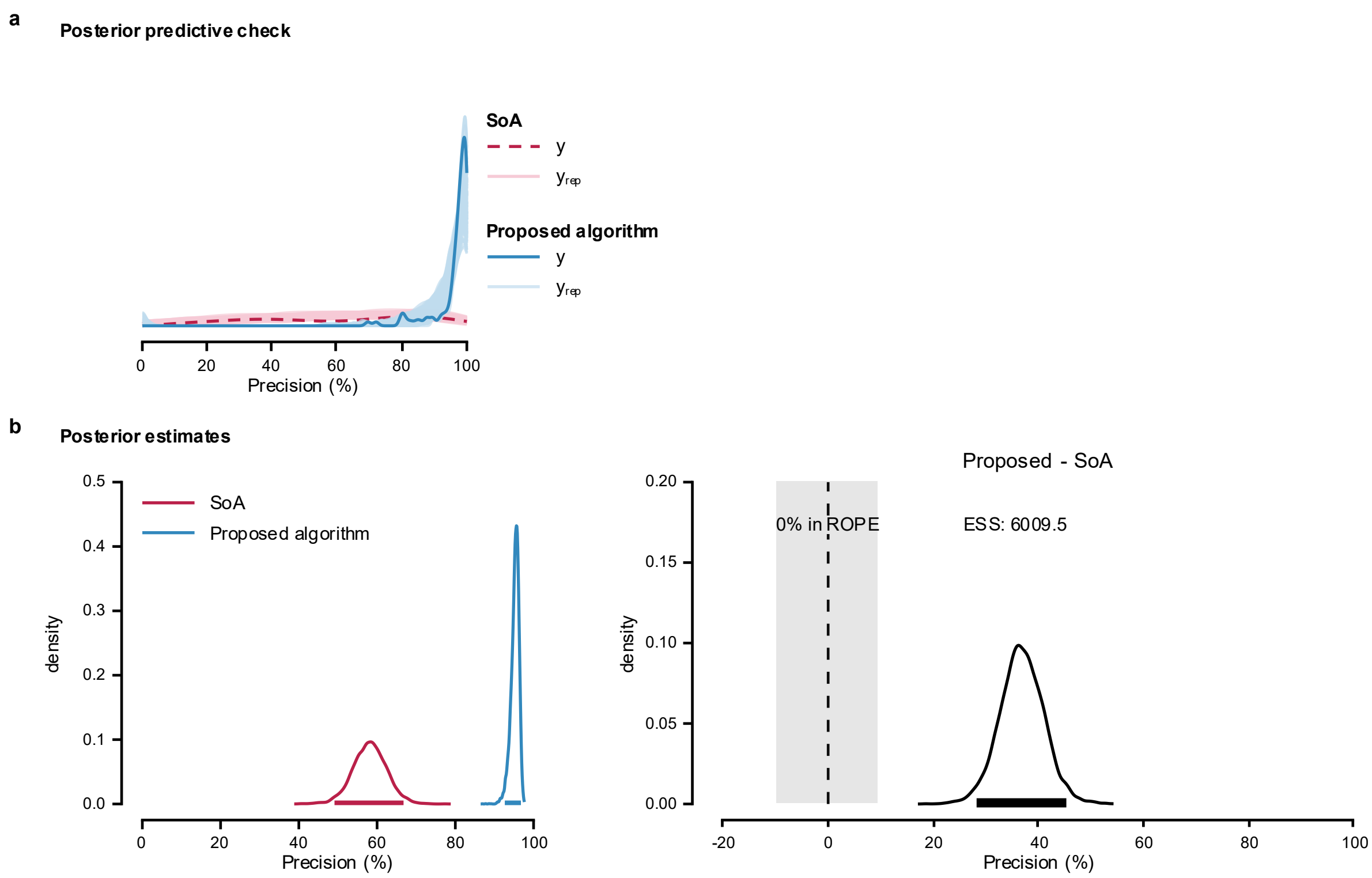

Supplementary Fig. 49: Posterior predictive check and posterior estimates for Model 2 of firing identification precision in tracked MG MUs across testing visits.
a, Posterior predictive check of the model; **b**, Posterior distributions and the difference between means estimated using Bayesian Model 2 to compare the performance of the proposed algorithm and SoA method.

<u>Model 3</u>

The maximum PSRF across all parameters was 1.002, indicating good convergence, while the minimum ESS was 4,792.538, indicating stable estimates of the bounds of the credible intervals (Supplementary Table 71). Posterior summaries are reported in Supplementary Table 72 as medians with 95% ETIs and 95% HDIs. Group-level correlations are reported on the correlation scale.

Posterior predictive checks were performed by overlaying model-generated predictions on the observed data (Supplementary Fig. 50a). The posterior distributions (Supplementary Fig.

50b) indicated that the proposed algorithm achieved a higher precision (95.2%, [92.6-96.8%]) than the SoA algorithm (58.3%, [49.2-66.8%]). The posterior distribution of the difference excluded zero, and less than 2.5% of its distribution fell within ROPE.

Supplementary Table 71: Posterior MCMC diagnostics for Model 3 of firing identification precision in tracked MG MUs across testing visits.

	**PSRF Pt**	**PSRF UpCI**	**ESS**
α_{μ}	1.000	1.000	1,0768.934
α_{ϕ}	1.000	1.001	1,2734.244
α_{ζ}	1.000	1.000	1,9212.704
α_{κ}	1.000	1.000	2,8112.325
$\beta_{\mu,\text{Proposed}}$	1.000	1.001	1,4713.291
$\beta_{\phi,\text{Proposed}}$	1.000	1.000	2,2458.397
$\beta_{\zeta,\text{Proposed}}$	1.000	1.000	2,0158.275
$\beta_{\kappa,\text{Proposed}}$	1.000	1.000	2,7228.644
τ_{b0}	1.001	1.002	6,214.410
$\tau_{b\text{Proposed}}$	1.000	1.001	6,278.603
τ_{c}	1.001	1.002	4,792.538
R_{12}	1.000	1.001	1,1848.773

Supplementary Table 72: Posterior summaries for Model 3 of firing identification precision in tracked MG MUs across testing visits.

	50%	95% ETI		Mode	95% HDI	
		2.5%	97.5%		low	high
α_{μ}	0.586	0.491	0.671	0.589	0.495	0.673
α_{ϕ}	3.059	2.312	4.000	2.999	2.269	3.938
α_{ζ}	0.021	0.005	0.055	0.016	0.003	0.049
α_{κ}	0.535	0.074	0.947	0.658	0.078	0.950
$\beta_{\mu,\text{Proposed}}$	15.207	10.086	22.128	14.864	9.685	21.579
$\beta_{\phi,\text{Proposed}}$	6.508	4.238	9.882	6.256	4.043	9.542
$\beta_{\zeta,\text{Proposed}}$	9.504	3.002	41.608	6.014	1.587	31.326
$\beta_{\kappa,\text{Proposed}}$	17.422	0.599	680.230	75.864	0.031	357.144
τ_{b0}	0.446	0.156	0.876	0.415	0.142	0.856
$\tau_{b\text{Proposed}}$	0.318	0.020	0.786	0.327	0.000	0.699
τ_{c}	0.110	0.005	0.392	0.039	0.000	0.338
R_{12}	-0.595	-0.952	0.467	-0.816	-0.991	0.288

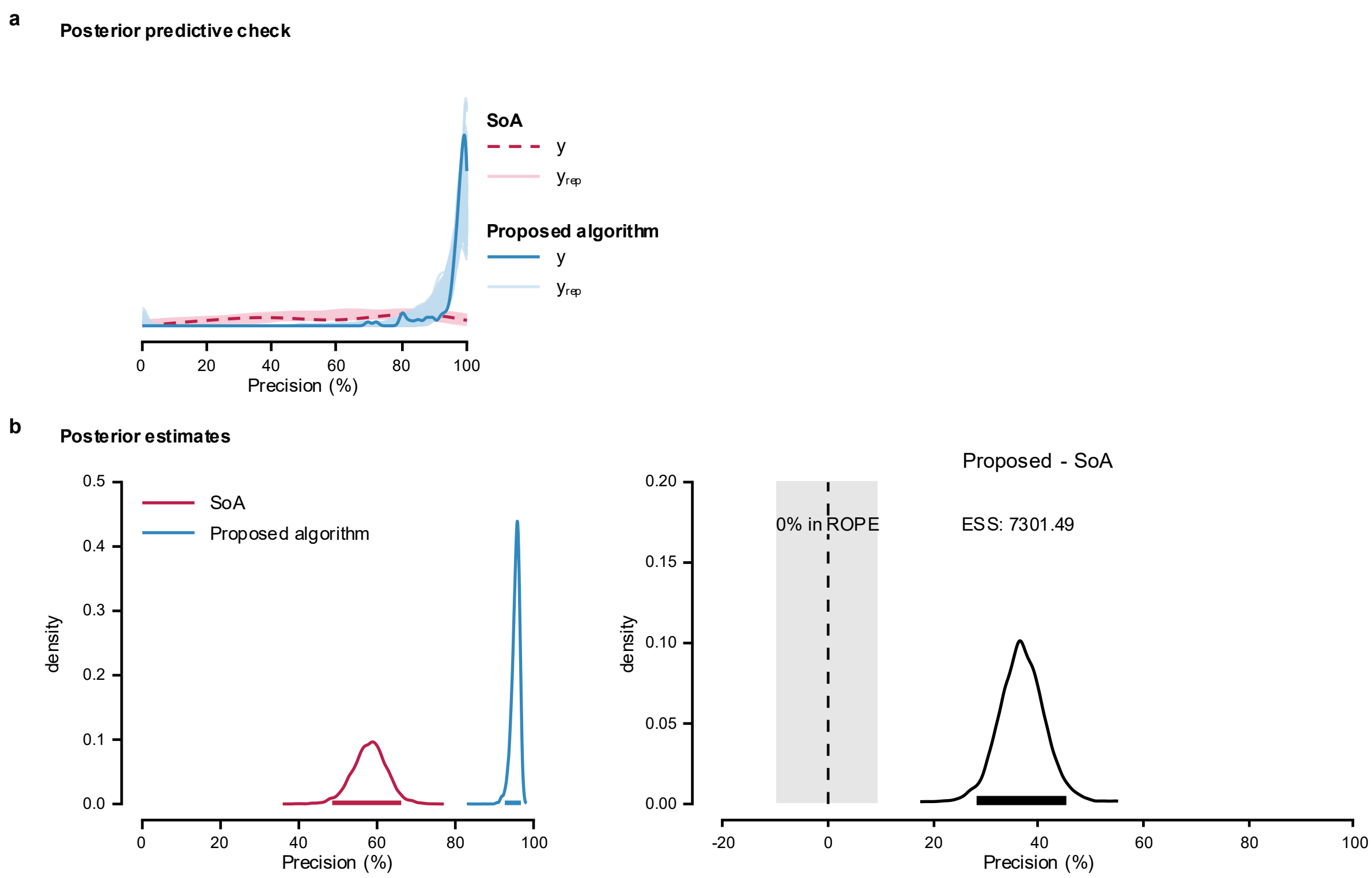

Supplementary Fig. 50: Posterior predictive check and posterior estimates for Model 3 of firing identification precision in tracked MG MUs across testing visits.

a, Posterior predictive check of the model; **b**, Posterior distributions and the difference between means estimated using Bayesian Model 1 to compare the performance of the proposed algorithm and SoA method.

MODEL COMPARISON

Model 2 showed the greatest expected predictive accuracy. ELPD diff relative to Model 2 were −0.7 (SE = 0.3) for Model 3 and −2.2 (SE = 1.5) for Model 1, suggesting similar predictive performance for all models.

Supplementary Note 18. Bayesian analysis R session info

```
R version 4.5.3 (2,026-03-11 ucrt)
Platform: x86_64-w64-mingw32/x64
Running under: Windows 10 x64 (build 1,9045)

Matrix products: default
  LAPACK version 3.12.1

locale:
[1] LC_COLLATE=English_United States.utf8
[2] LC_CTYPE=English_United States.utf8
[3] LC_MONETARY=English_United States.utf8
[4] LC_NUMERIC=C
[5] LC_TIME=English_United States.utf8

time zone: America/Chicago
tzcode source: internal

attached base packages:
[1] stats     graphics  grDevices utils     datasets  methods   base

other attached packages:
 [1] rstudioapi_0.18.0 future_1.70.0     tidyr_1.3.2       tibble_3.3.1      car_3.1-5
 [6] HDInterval_0.2.4  posterior_1.7.0   coda_0.19-4.1     readxl_1.5.0      loo_2.9.0
[11] carData_3.0-6     gridExtra_2.3     ggplot2_4.0.3     dplyr_1.2.1       brms_2.23.0
```

```
[16] bayesplot_1.15.0   Rcpp_1.1.1

loaded via a namespace (and not attached):
 [1] tidyselect_1.2.1      viridisLite_0.4.3     farver_2.1.2          S7_0.2.1
 [5] fastmap_1.2.0         lazyeval_0.2.3        TH.data_1.1-5         tensorA_0.36.2.1
 [9] digest_0.6.39         estimability_1.5.1    lifecycle_1.0.5       StanHeaders_2.32.10
[13] survival_3.8-6        processx_3.9.0        magrittr_2.0.5        compiler_4.5.3
[17] rlang_1.2.0           tools_4.5.3           utf8_1.2.6            data.table_1.18.2.1
[21] knitr_1.51            labeling_0.4.3        bridgesampling_1.2-1  htmlwidgets_1.6.4
[25] pkgbuild_1.4.8        plyr_1.8.9            RColorBrewer_1.1-3    multcomp_1.4-30
[29] abind_1.4-8           withr_3.0.2           purrr_1.2.2           future.apply_1.20.2
[33] stats4_4.5.3          inline_0.3.21         globals_0.19.1        emmeans_2.0.3
[37] scales_1.4.0          MASS_7.3-65           cli_3.6.6             mvtnorm_1.3-7
[41] crayon_1.5.3          generics_0.1.4        otel_0.2.0            RcppParallel_5.1.11-2
[45] grid_4.5.3            reshape2_1.4.5        httr_1.4.8            rstan_2.32.7
[49] stringr_1.6.0         splines_4.5.3         parallel_4.5.3        cellranger_1.1.0
[53] matrixStats_1.5.0     vctrs_0.7.3           Matrix_1.7-4          sandwich_3.1-1
[57] jsonlite_2.0.0        callr_3.7.6           Formula_1.2-5         listenv_0.10.1
[61] plotly_4.12.0         parallelly_1.47.0     glue_1.8.0            codetools_0.2-20
[65] gtable_0.3.6          QuickJSR_1.9.2        distributional_0.7.0  stringi_1.8.7
[69] pillar_1.11.1         htmltools_0.5.9       Brobdingnag_1.2-9     R6_2.6.1
[73] evaluate_1.0.5        lattice_0.22-9        backports_1.5.1       openxlsx_4.2.8.1
[77] rstantools_2.6.0      zip_2.3.3             nlme_3.1-168          checkmate_2.3.4
[81] xfun_0.57             zoo_1.8-15            pkgconfig_2.0.3
```

Supplementary Note 19. Linear mixed effect model for the rate and recruitment threshold of tracked MUs across follicular phase

The data contained 189 observations of MU firing rates (FR) and recruitment thresholds (RT) from 9 participants. The MUs were tracked across three visits during the follicular phase (EF1, EF2, and OV). FR and RT were analyzed separately. MUs that were only partially active within the 5 second window of stable torque production or were active outside that window, were not excluded from the analysis. The model was specified as follows

$$Y \sim \text{Visit} + (1 \mid \text{Subject:MUID}) \tag{S50}$$

where Y denotes the predicted variable (FR or RT) and MUID denotes the MU identification number. Model diagnostics were assessed visually using scaled Pearson residuals. Residual-versus-fitted plot and residuals by Visit were examined for systematic patterns and heteroscedasticity. Q-Q plots assessed the normality of residuals and MU-within-Subject conditional random effects, with 95 % pointwise confidence envelopes.

Linear mixed effect model for the MU firing rates

Standardized residuals were broadly centered around zero across fitted values, with no pronounced nonlinear pattern or evidence of heteroscedasticity, despite a slight upward trend (Supplementary Fig. 51). Residuals were similarly centered and dispersed across Visits, suggesting that this trend did not indicate substantial model misspecification. The Q–Q plot supported approximate residual normality: most points followed the theoretical quantiles and fell within the 95% pointwise confidence envelope, with only minor tail deviations. The model had a marginal R^2 of 0.009 and a conditional R^2 of 0.498. FR did not differ significantly across the follicular phase ($p = 0.180$, Supplementary Table 73). Response-scale estimated marginal means (EMM) for each Visit and model-scale pairwise comparison are provided in Supplementary Table 74 and Supplementary Table 75, respectively.

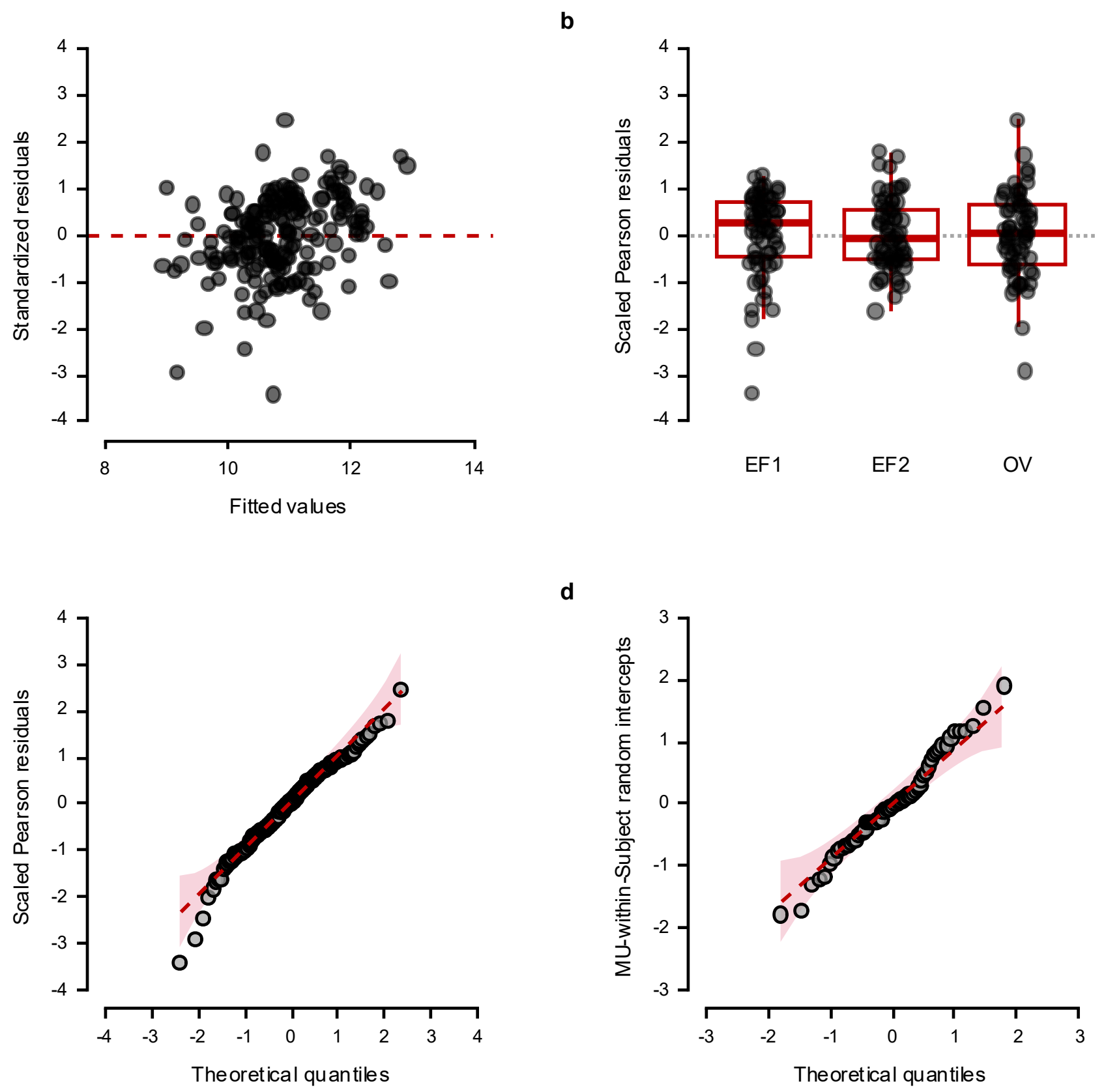

Supplementary Fig. 51: LMM of tracked MUs FR diagnostics plots.

a, Pearson residuals vs fitted values, **b**, residual by visit, **c**, Q-Q plot of scaled Pearson residuals, and **d**, Q-Q plot of random effects.

Supplementary Table 73: Type III Analysis of Variance with Satterthwaite's method of the LMM of tracked MUs FR.

	Sum Sq	Mean Sq	NumDF	DenDF	F value	Pr(>F)
Visit	2.745	1.372	2	124	1.738	0.18

Supplementary Table 74: EMM in response scale for each visit of the LMM of tracked MUs FR.

Visit	EMM	SE	95% CI
EF1	10.7	0.157	10.4 – 11.0
EF2	11.0	0.157	10.7 – 11.3
OV	10.9	0.157	10.6 – 11.2

Supplementary Table 75: Tukey-adjusted pairwise comparisons on the model scale of the LMM of tracked MUs FR.

contrast	estimate	SE	df	t ratio	p-value
EF1 – EF2	-0.292	0.158	124	-1.843	0.160
EF1 – OV	-0.185	0.158	124	-1.167	0.475
EF2 – OV	0.107	0.158	124	0.676	0.778

<u>Linear mixed effect model for the MU recruitment threshold</u>

A square root transformation was applied to the recruitment threshold because it improved the residual diagnostics (Supplementary Fig. 52). Standardized residuals were broadly centered around zero, with a slight upward trend. However, residuals by Visits showed no evidence of heteroscedasticity. The Q-Q plot indicated approximate normality: most scaled Pearson residuals closely followed the theoretical quantiles and fell within the 95% pointwise confidence envelope, with minor tail deviations. The model had a marginal R^2 of 0.059 and a conditional R^2 of 0.565.

Unlike the FR, RT showed a significant overall Visit effect in the Type III Analysis of Variance (ANOVA) using Satterthwaite's method (Supplementary Table 76), supported by

pairwise comparisons (p < 0.001, Supplementary Table 77). Response-scale EMMs are provided in Supplementary Table 78.

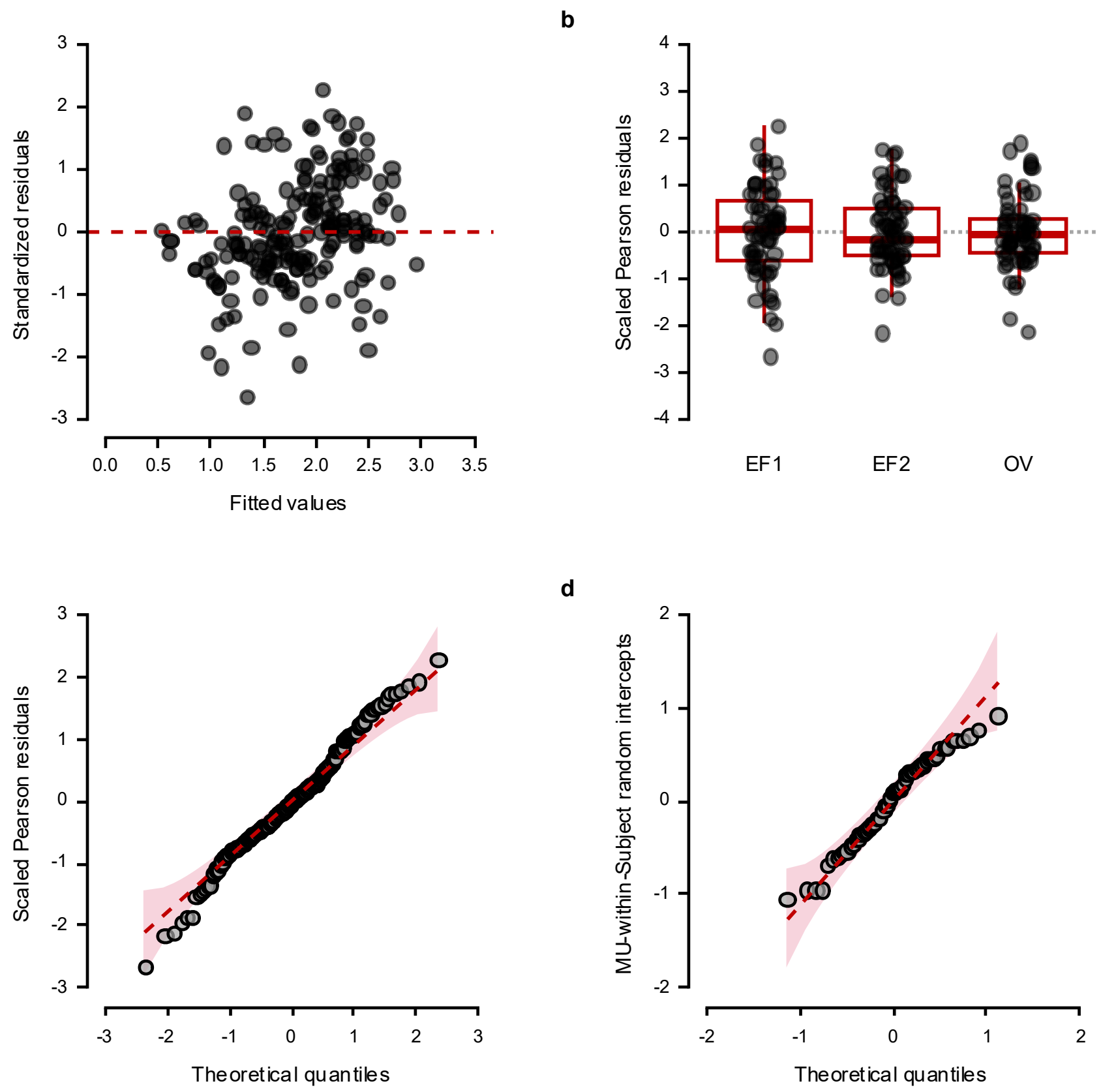

Supplementary Fig. 52: LMM of tracked MUs RT diagnostics plots.

a, Pearson residuals vs fitted values, **b**, residual by visit, **c**, Q-Q plot of scaled Pearson residuals, and **d**, Q-Q plot of random effects.

Supplementary Table 76: Type III Analysis of Variance with Satterthwaite's method of the LMM of tracked MUs RT.

	Sum Sq	Mean Sq	NumDF	DenDF	F value	Pr(>F)
Visit	6.400	3.200	2	124	12.684	< 0.001*

Supplementary Table 77: Tukey-adjusted pairwise comparisons on the model scale of the LMM of tracked MUs RT.

contrast	estimate	SE	df	t ratio	p-value
EF1 – EF2	0.451	0.089	124	5.036	< 0.001*
EF1 – OV	0.219	0.089	124	2.448	0.041*
EF2 – OV	-0.232	0.089	124	-2.588	0.029*

Supplementary Table 78: EMM in response scale for each visit of the LMM of tracked MUs RT.

Visit	EMM	SE	95% CI
EF1	4.13	0.378	3.41 – 4.91
EF2	2.50	0.294	1.95 – 3.12
OV	3.29	0.338	2.65 – 3.99

Supplementary Note 20. Linear mixed effect model of firing rate and recruitment threshold of all identified motor units across follicular phase

The data contained 393 observations of MU FR and RT from 9 participants. The MUs were tracked across three visits during the follicular phase (EF1, EF2, and OV). FR and RT were analyzed separately. MUs that were only partially active within the 5 second window of stable torque production or were active outside that window, were not excluded from the analysis. The model was specified as follows

$$Y \sim \text{Visit} + (1 \mid \text{Subject}) \tag{S51}$$

where Y is the predicted variable (FR or RT). Because MUs were not tracked across visits in all-observation analysis, MU-level repeated measure could not be specified as in the tracked MU case (Supplementary Note 19). This limited our ability to model within-MU correlation or MU-specific baseline differences across visits. Therefore, the model included Subject as the only random intercept, accounting for clustering of observations within Subject.

Model diagnostics were assessed visually using scaled Pearson residuals. Residual-versus-fitted plot and residuals by Visit were examined for systematic patterns and heteroscedasticity. Q-Q plots assessed the normality of residuals and within-Subject conditional random effects, with 95 % pointwise confidence envelopes.

<u>Linear mixed effect model for the MU firing rates</u>

Standardized residuals were broadly centered around zero across the range of fitted values, with no strong evidence of heteroscedasticity (Supplementary Fig. 53). Residuals showed similar spread across Visit, and the vertical bands were expected given the categorical predictor. The Q-Q plot supported approximate normality: most scaled Pearson residuals collowed the theoretical quantiles and fell within the 95% pointwise confidence envelope, with only minor tail deviations. The model had a marginal R^2 of 0.012 and a conditional R^2 of

0.067. The results suggest that the FR was not significantly different across the follicular phase (Supplementary Table 79). Response-scale EMMs and model scale pairwise are provided in Supplementary Table 80 and Supplementary Table 81, respectively.

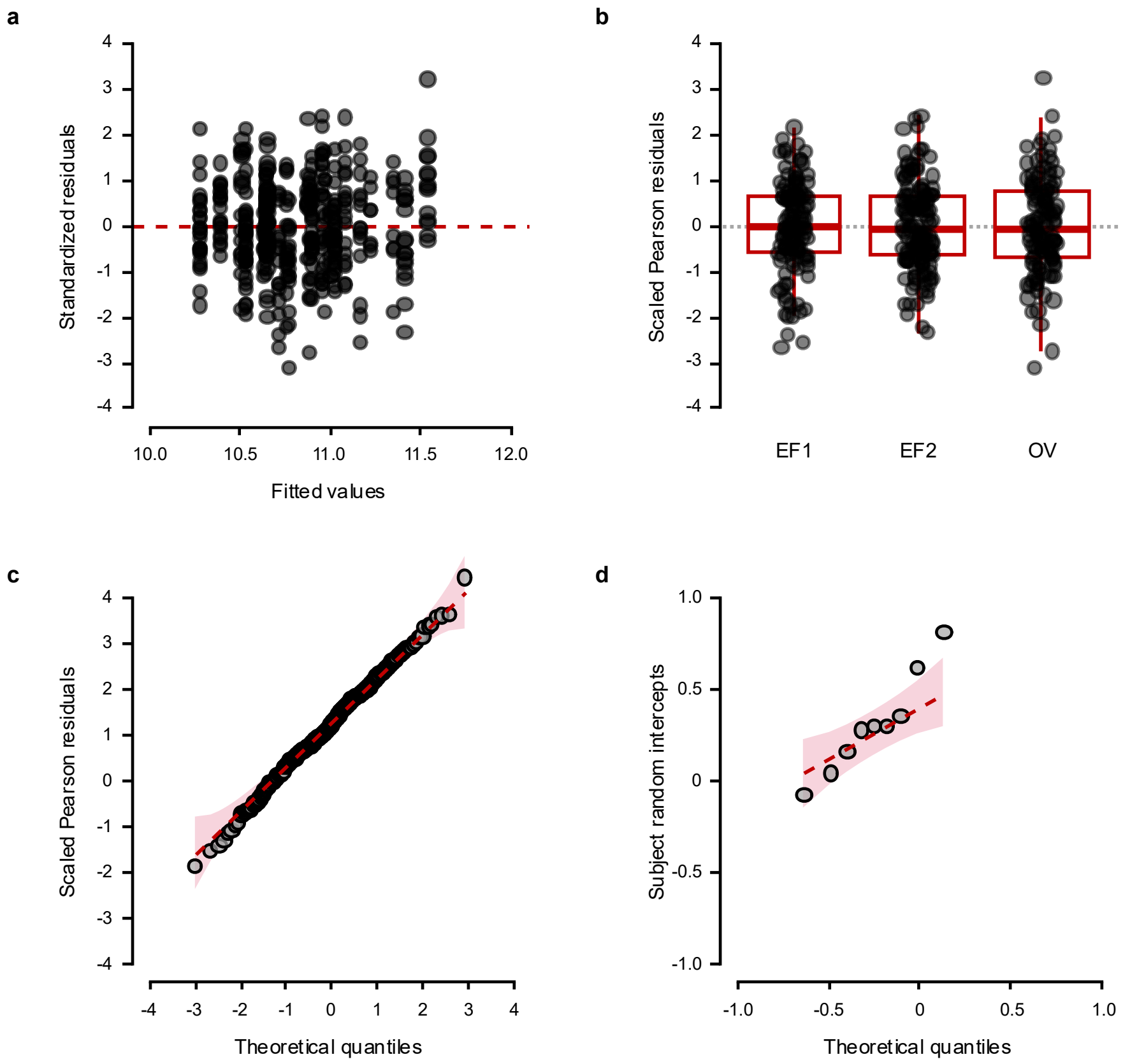

Supplementary Fig. 53: LMM of all identified MUs FR diagnostics plots.
a, Pearson residuals vs fitted values, **b**, residual by visit, **c**, Q-Q plot of scaled Pearson residuals, and **d**, Q-Q plot of random effects.

Supplementary Table 79: Type III Analysis of Variance with Satterthwaite's method of the LMM of all identified MUs FR.

	Sum Sq	Mean Sq	NumDF	DenDF	F value	Pr(>F)
Visit	9.081	4.540	2	384.29	2.494	0.084

Supplementary Table 80: EMM in response scale for each visit of the LMM of all identified MUs FR.

Visit	EMM	SE	95% CI
EF1	10.7	0.162	10.3 – 11.0
EF2	10.9	0.159	10.6 – 11.2
OV	11.0	0.165	10.7 – 11.4

Supplementary Table 81: Tukey-adjusted pairwise comparisons on the model scale of the LMM of all identified MUs FR.

contrast	estimate	SE	df	t ratio	p-value
EF1 – EF2	-0.247	0.164	384	-1.500	0.292
EF1 – OV	-0.373	0.171	384	-2.184	0.075
EF2 – OV	-0.127	0.167	385	-0.757	0.729

Linear mixed effect model for the MU recruitment threshold

A square root transformation was applied to the recruitment threshold because it improved the residual diagnostics (Supplementary Fig. 54). Standardized residuals were broadly centered around zero with similar spread across Visit. The Q-Q plot indicated generally reasonable normality, with mild upper-tail deviation. The model had a marginal R^2 of 0.020 and a conditional R^2 of 0.106.

Type III ANOVA with Satterthwaite's method revealed a significant overall Visit effect on RT (Supplementary Table 82) which was confirmed with the pairwise comparison (Supplementary Table 83). The EMM in response scale is provided in Supplementary Table 84.

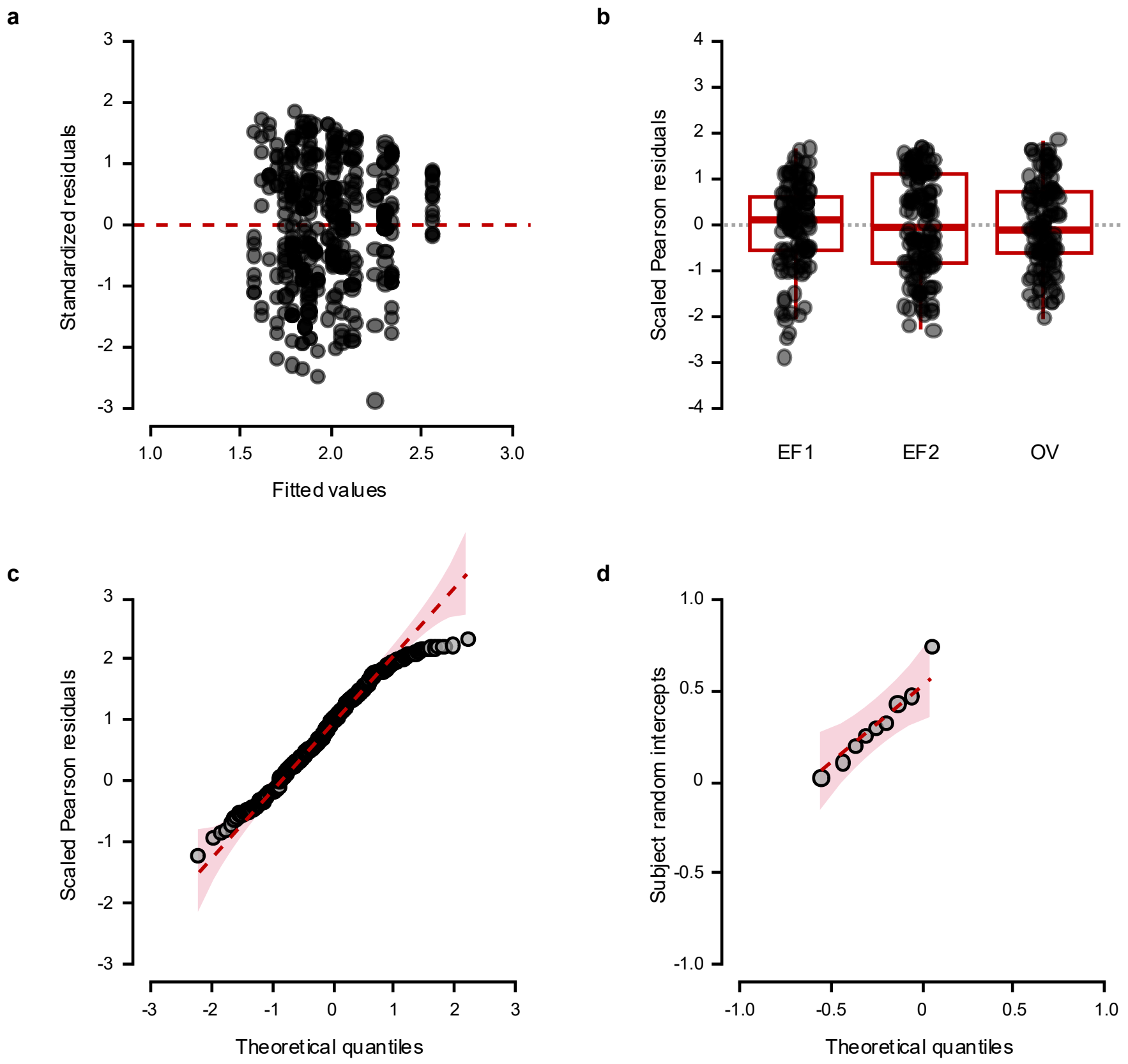

Supplementary Fig. 54: LMM of all identified MUs RT diagnostics plots.

a, Pearson residuals vs fitted values, **b**, residual by visit, **c**, Q-Q plot of scaled Pearson residuals, and **d**, Q-Q plot of random effects.

Supplementary Table 82: Type III Analysis of Variance with Satterthwaite's method of the LMM of all identified MUs RT.

	Sum Sq	Mean Sq	NumDF	DenDF	F value	Pr(>F)
Visit	5.256	2.628	2	383.28	4.367	0.013*

Supplementary Table 83: Tukey-adjusted pairwise comparisons on the model scale of the LMM of all identified MUs RT.

contrast	estimate	SE	df	t ratio	p-value
EF1 – EF2	0.228	0.095	383	2.413	0.043*
EF1 – OV	0.264	0.098	383	2.688	0.020*
EF2 – OV	0.036	0.096	384	0.374	0.926

Supplementary Table 84: EMM in response scale for each visit of the LMM of all identified MUs RT.

Visit	EMM	SE	95% CI
EF1	4.55	0.453	3.64 – 5.57
EF2	3.63	0.399	2.82 – 4.54
OV	3.49	0.401	2.69 – 4.40

Supplementary References

1. Holobar, A., Farina, D., Gazzoni, M., Merletti, R. & Zazula, D. Estimating motor unit discharge patterns from high-density surface electromyogram. *Clin. Neurophysiol.* **120**, 551–562 (2009).
2. Holobar, A. & Zazula, D. Multichannel blind source separation using convolution Kernel compensation. *IEEE Trans. Signal Process.* **55**, 4487–4496 (2007).
3. Del Vecchio, A. *et al.* Tutorial: Analysis of motor unit discharge characteristics from high-density surface EMG signals. *J. Electromyogr. Kinesiol.* **53**, 102426 (2020).
4. Holobar, A., Minetto, M. A. & Farina, D. Accurate identification of motor unit discharge patterns from high-density surface EMG and validation with a novel signal-based performance metric. *J. Neural Eng.* **11**, 016008 (2014).
5. Francic, A. & Holobar, A. On the Reuse of Motor Unit Filters in High Density Surface Electromyograms Recorded at Different Contraction Levels. *IEEE Access* **9**, 115227–115236 (2021).
6. Keogh, E. & Ratanamahatana, C. A. Exact indexing of dynamic time warping. *Knowl. Inf. Syst.* **7**, 358–386 (2005).
7. Afsharipour, B., Soedirdjo, S. & Merletti, R. Two-dimensional surface EMG: The effects of electrode size, interelectrode distance and image truncation. *Biomed. Signal Process. Control* **49**, 298–307 (2019).
8. Proakis, J. G. & Manolakis, D. K. *Digital Signal Processing (4th Ed.)*. (Prentice-Hall, Inc., 2006).
9. Blok, J. H., van Dijk, J. P., Drost, G., Zwarts, M. J. & Stegeman, D. F. A high-density multichannel surface electromyography system for the characterization of single motor units. *Rev. Sci. Instrum.* **73**, 1887 (2002).
10. Brooks, S. P. & Gelman, A. General methods for monitoring convergence of iterative

simulations)? *J. Comput. Graph. Stat.* **7**, 434–455 (1998).

11. Plummer, M., Best, N., Cowles, K. & Vines, K. {CODA}: Convergence Diagnosis and Output Analysis for {MCMC}. *R News* **6**, 7–11 (2006).

12. Kruschke, J. K. *Doing Bayesian data analysis: A tutorial with R, JAGS, and Stan*. (Academic Press, 2015).

13. Vehtari, A., Gelman, A., Simpson, D., Carpenter, B. & Bürkner, P.-C. Rank-Normalization, Folding, and Localization: An Improved Rˆ for Assessing Convergence of MCMC (with Discussion). *Bayesian Anal.* **16**, 667–718 (2021).

14. Vehtari, A., Gelman, A. & Gabry, J. Practical Bayesian model evaluation using leave-one-out cross-validation and WAIC. *Stat. Comput.* **27**, 1413–1432 (2017).
